# Systematic evaluation of single-cell foundation model interpretability: attention-derived edge scores add no incremental value over gene-level features for perturbation-target prediction

**DOI:** 10.1186/s12864-026-12965-8

**Published:** 2026-07-22

**Authors:** Ihor Kendiukhov

**Affiliations:** Institute of Medical Genetics and Applied Genomics, University of Tübingen, Tübingen, Germany

**Keywords:** Single-cell foundation models, Mechanistic interpretability, Gene regulatory networks, Attention mechanisms, ScGPT, Geneformer, Perturbation prediction, CRISPR screens, Benchmarking

## Abstract

**Background:**

Single-cell foundation models such as scGPT and Geneformer are increasingly used for gene regulatory network (GRN) inference, with attention-derived edge scores routinely interpreted as regulatory proxies. Prior benchmarks have evaluated curated-reference recovery but have not systematically tested whether attention adds information beyond expression statistics for predicting the outcomes of genetic perturbations, nor whether attention-identified “regulatory” components are causally required for such predictions. This gap matters because the NLP interpretability literature has established that attention weights do not reliably indicate feature importance, and biological foundation models are being deployed without analogous scrutiny.

**Methods:**

We present an evaluation framework comprising thirty-seven analyses and 153 statistical tests under Benjamini-Hochberg FDR correction, spanning two architectures (scGPT, Geneformer V2-316M), four cell types (K562, RPE1, primary T cells, iPSC neurons), and two perturbation modalities (CRISPRi, CRISPRa). The framework separates two objectives: (A) mechanistic interpretability / GRN recovery against curated references, and (B) perturbation-target prediction, i.e. classifying which genes show differential expression after a CRISPR perturbation. Five test families—trivial-baseline comparison, conditional incremental-value testing, residualisation and propensity matching, causal ablation with intervention-fidelity diagnostics, and cross-context replication—address Objective B, supplemented by a synthetic positive control establishing pipeline sensitivity.

**Results:**

Attention patterns encode layer-specific biological structure—protein–protein interactions in early layers, transcriptional regulation in late layers—and Cell-State Stratified Interpretability (CSSI) exploits this structure to improve curated GRN recovery up to $1.85\times$ on the Objective A task. On Objective B, however, attention-derived edge scores add no incremental value beyond trivial gene-level features (variance, mean expression, dropout rate): gene-level baselines outperform both attention and correlation edges (AUROC 0.81–0.88 versus 0.70), augmenting gene-level predictors with pairwise edges produces $\Delta$AUROC of $-0.0004$ to $-0.002$ across 559,720 perturbation–gene observations, and causal ablation of TRRUST-ranked attention heads produces no degradation across three independent intervention channels. The attention–correlation relationship is context-dependent (equal in K562 CRISPRi, worse in CRISPRa, better in RPE1), but gene-level dominance on Objective B is universal across both cell types where adequate power is available.

**Conclusions:**

Attention patterns in single-cell foundation models encode biologically structured information, including layer-specific regulatory signals recoverable via CSSI, but provide no unique predictive information beyond simple gene-level statistics for the perturbation-target prediction task. The paper thus offers both a cautionary finding for Objective B and a constructive method (CSSI) for Objective A. Practitioners should apply trivial-baseline and incremental-value tests before claiming pairwise regulatory signal, and should stratify by cell state when extracting attention-derived GRNs.

**Supplementary Information:**

The online version contains supplementary material available at 10.1186/s12864-026-12965-8.

## Background

The emergence of transformer-based foundation models for single-cell transcriptomics represents a paradigm shift in computational biology [1–4]. These models—trained on millions of cells across diverse tissues—learn contextual representations that have shown promise for cell type annotation, perturbation response prediction, and gene regulatory network (GRN) inference [5, 6]. A particularly compelling promise is *mechanistic interpretability*: extracting biologically meaningful regulatory circuits directly from attention-derived edge scores. Both scGPT [1] and Geneformer [2] highlight attention-derived gene network inference as a key application, and downstream studies have adopted attention-derived edge scores as regulatory proxies without rigorous validation [7].

This promise draws on parallel advances in large language model interpretability, where techniques such as activation patching [8, 9] and automated circuit discovery [10] have identified computational circuits for well-defined behaviours [11–13]. However, translating these approaches to biology faces unique challenges. Gene regulatory relationships are context-dependent, combinatorial, and only partially captured in reference databases such as TRRUST [14] and DoRothEA [15], which contain a fraction of true regulatory interactions [16].

Current practices in single-cell foundation model interpretability rest on several critical and largely untested assumptions. *First*, that attention patterns directly reflect causal regulatory relationships—an assumption already challenged in the NLP literature [17–19]. *Second*, that larger datasets consistently improve the reliability of mechanistic interpretations. *Third*, that attention-derived predictions align with experimental perturbation outcomes from CRISPR screens [20]. *Fourth*, that mechanistic insights transfer reliably across biological contexts. We address these through a two-tier evaluation framework. The *core tier* directly evaluates foundation model internal representations—attention weights, intervention effects, and perturbation-outcome prediction—and proposes Cell-State Stratified Interpretability (CSSI) as a constructive method. The *boundary condition tier* uses correlation-based edge scores to establish limits that any edge-scoring method must contend with, including cross-species transfer, pseudotime directionality, technical leakage, and uncertainty calibration (Additional file 1: Supplementary Notes 7–10).

Prior benchmarks have evaluated GRN inference methods [16] and individual foundation model capabilities [7], but none has systematically assessed whether attention-derived edge scores add information beyond expression statistics for predicting perturbation outcomes, nor tested this with causal interventions. We address this gap with a reusable evaluation framework integrating thirty-seven complementary analyses—including trivial-baseline comparison, conditional incremental-value testing, expression residualisation, propensity-matched benchmarks, and causal ablation with intervention-fidelity diagnostics—across two foundation model architectures (scGPT, Geneformer V2-316M), four cell types (K562, primary T cells, RPE1 retinal epithelial, iPSC neurons), and two perturbation modalities (CRISPRi, CRISPRa), with 153 statistical tests under Benjamini-Hochberg FDR correction (Additional file 1: Supplementary Table 1; Supplementary Note 16). The framework distinguishes two objectives explicitly (see §*Evaluation framework*): Objective A asks whether attention edges recover curated regulatory interactions (TRRUST, DoRothEA); Objective B asks whether they predict which genes show differential expression after a CRISPR perturbation. These are mathematically distinct tasks—edge identification versus effect-magnitude prediction—and they can, and in our results do, give different answers.

The framework yields a three-part finding. First (Objective A), attention patterns encode biologically structured information with a clean layer-specific organisation—protein–protein interactions in early layers, transcriptional regulation in late layers *(exploratory)* (Additional file 1: Supplementary Note 17)—and Cell-State Stratified Interpretability (CSSI) exploits this structure to improve curated-reference GRN recovery up to $1.85\times$ over unstratified baselines. Second (Objective B), on the perturbation-target prediction task, this layer-specific structure provides no incremental value over trivial gene-level features: univariate baselines outperform both attention and correlation edges, pairwise edge scores add zero predictive contribution when combined with gene-level features, and causal ablation of TRRUST-ranked “regulatory” heads produces no behavioural effect across three independent intervention channels. A synthetic positive control (Supplementary Note 21) confirms that the evaluation pipeline is sensitive: it detects planted pairwise regulatory signal when such signal exists. Third, the attention–correlation relationship is context-dependent across cell types (equal in K562 CRISPRi, worse in CRISPRa, better in RPE1), but the Objective B gene-level dominance pattern generalises across both cell types where adequate power is available. The framework itself—its battery of tests, controls, and diagnostic checks—constitutes a reusable quality-control standard for evaluating mechanistic interpretability claims in single-cell foundation models.

## Results

### Evaluation framework: two objectives, five test families

**Fig. 1:**
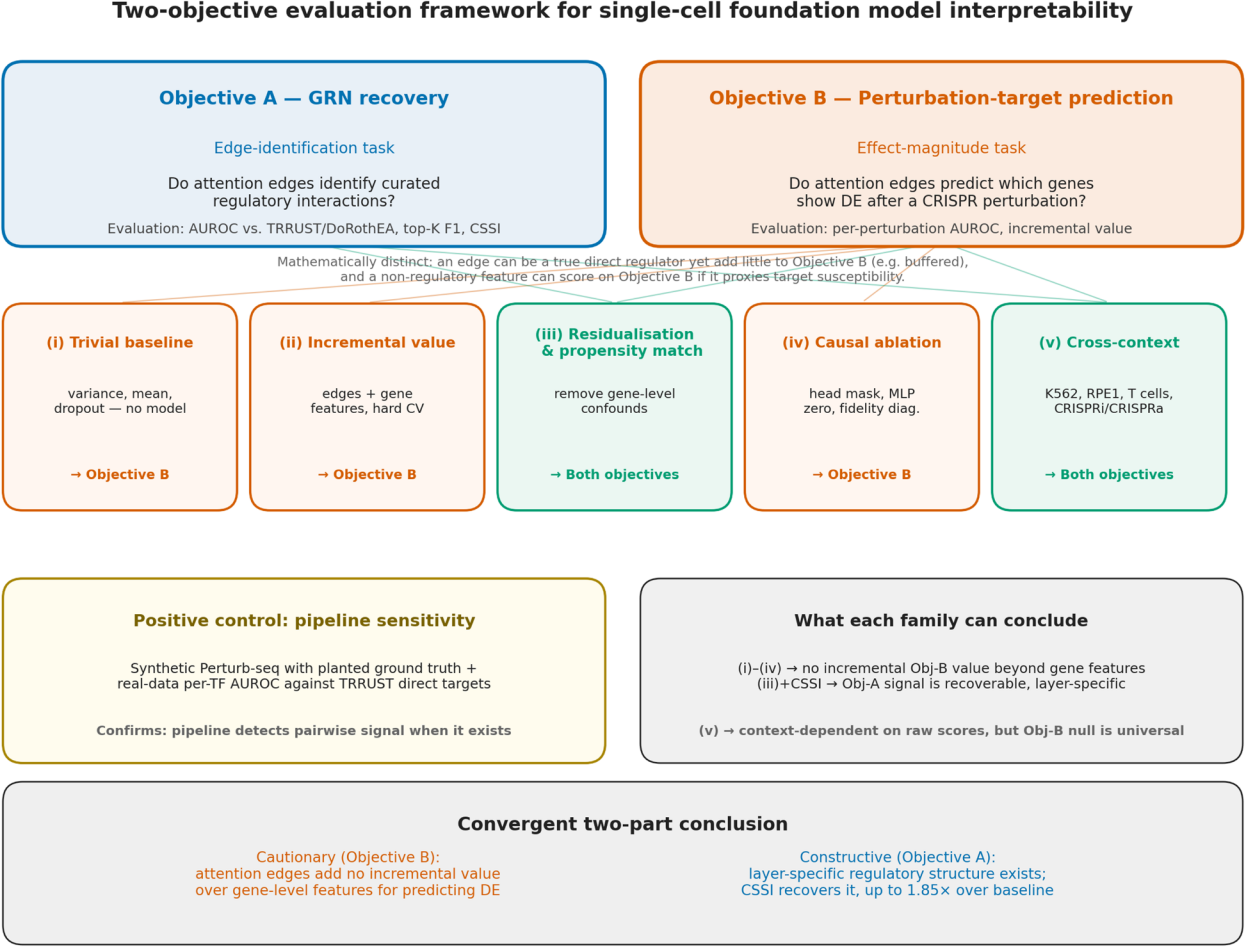
Two-objective evaluation framework. The framework explicitly separates two functional objectives. Objective A (GRN recovery) asks whether attention edges identify curated regulatory interactions, evaluated against TRRUST/DoRothEA. Objective B (perturbation-target prediction) asks whether attention edges predict which genes show differential expression after a CRISPR perturbation. The two are mathematically distinct: an edge can be a true direct regulator yet add little to Objective B (e.g. buffered), and a non-regulatory feature can score on Objective B if it proxies target susceptibility. Five test families operationalise Objective B with one (residualisation) addressing both. A positive-control synthetic pipeline establishes that the Objective B evaluation is sensitive when planted ground truth exists. The two-part conclusion is shown at the bottom: cautionary on Objective B (no incremental value beyond gene features), constructive on Objective A (CSSI recovers layer-specific regulatory structure)

We evaluate single-cell foundation models on two explicitly separated functional objectives, because these measure different biological quantities and can give different answers (Fig. 1).

**Objective A (mechanistic interpretability/GRN recovery).** Can attention-derived edge scores identify curated regulatory interactions? This is an edge-*identification* task evaluated against reference databases (TRRUST [14], DoRothEA [15], STRING [21]) using AUROC and top-*K* F1 on directed TF–target pairs. TRRUST is our primary reference because its entries require direct experimental evidence (perturbation, reporter, or ChIP-seq), giving it the highest specificity among widely-used human TF–target resources; DoRothEA is reported as a broader but noisier complement. We address Objective A with scaling analyses, CSSI stratification, expression residualisation, and biological database benchmarking (Additional file 1: Supplementary Notes 1, 11, 17).

**Objective B (perturbation-response prediction).** Do attention-derived edge scores predict which genes will show differential expression after a CRISPRi or CRISPRa perturbation of a source gene? This is an effect-*magnitude* task that integrates direct regulation, indirect propagation, and target susceptibility. Evaluation uses per-perturbation AUROC for classifying DE hits, supplemented by incremental-value testing that asks whether edges add information beyond univariate gene-level features.

The two objectives are mathematically distinct: an edge can be a genuine direct regulatory link yet contribute little to Objective B (e.g., a buffered homeostatic edge produces minimal DE despite direct regulation), and conversely a non-regulatory pairwise feature can score well on Objective B if it is a good proxy for target susceptibility. A complete evaluation must therefore test both, and a result on one cannot be directly extrapolated to the other.

**Fig. 2:**
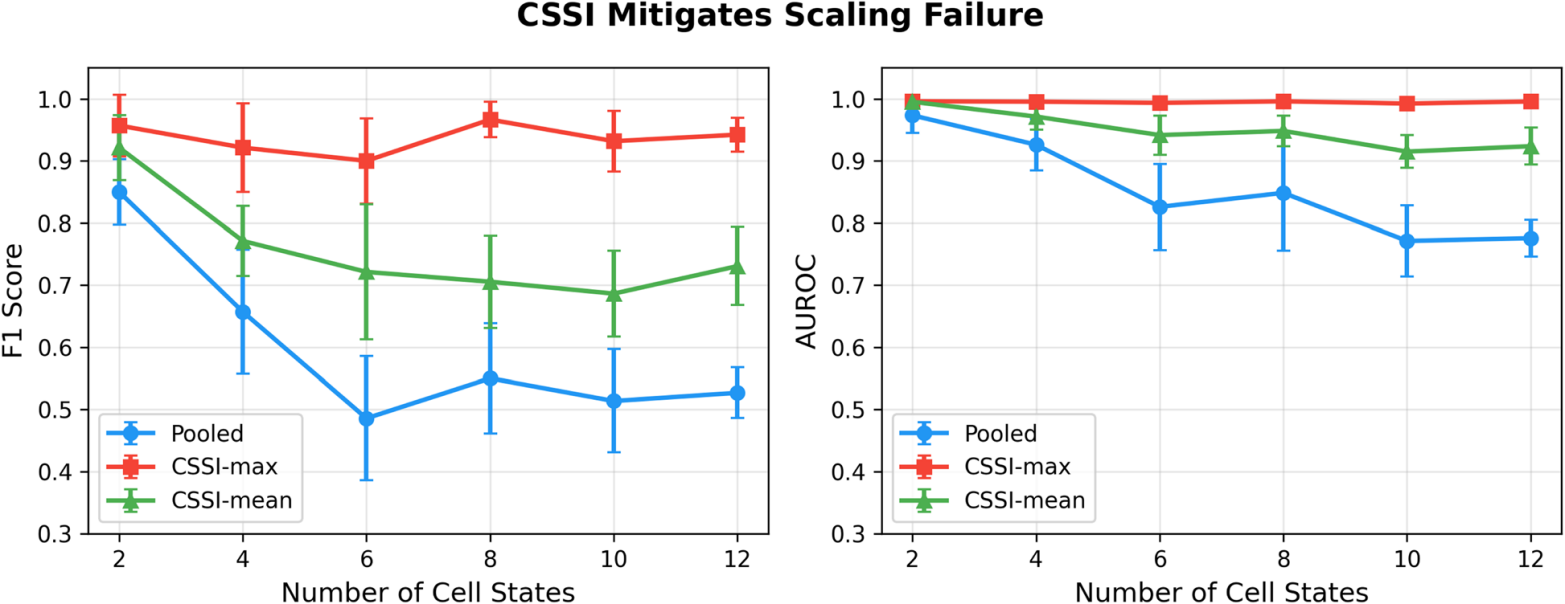
CSSI improves attention-derived GRN recovery. CSSI-max TRRUST F1 versus number of strata (*K*) on DLPFC brain data. Stratified scoring improves recovery up to $1.85\times$ over unstratified baselines, with optimal $K = 5$–7

**Fig. 3:**
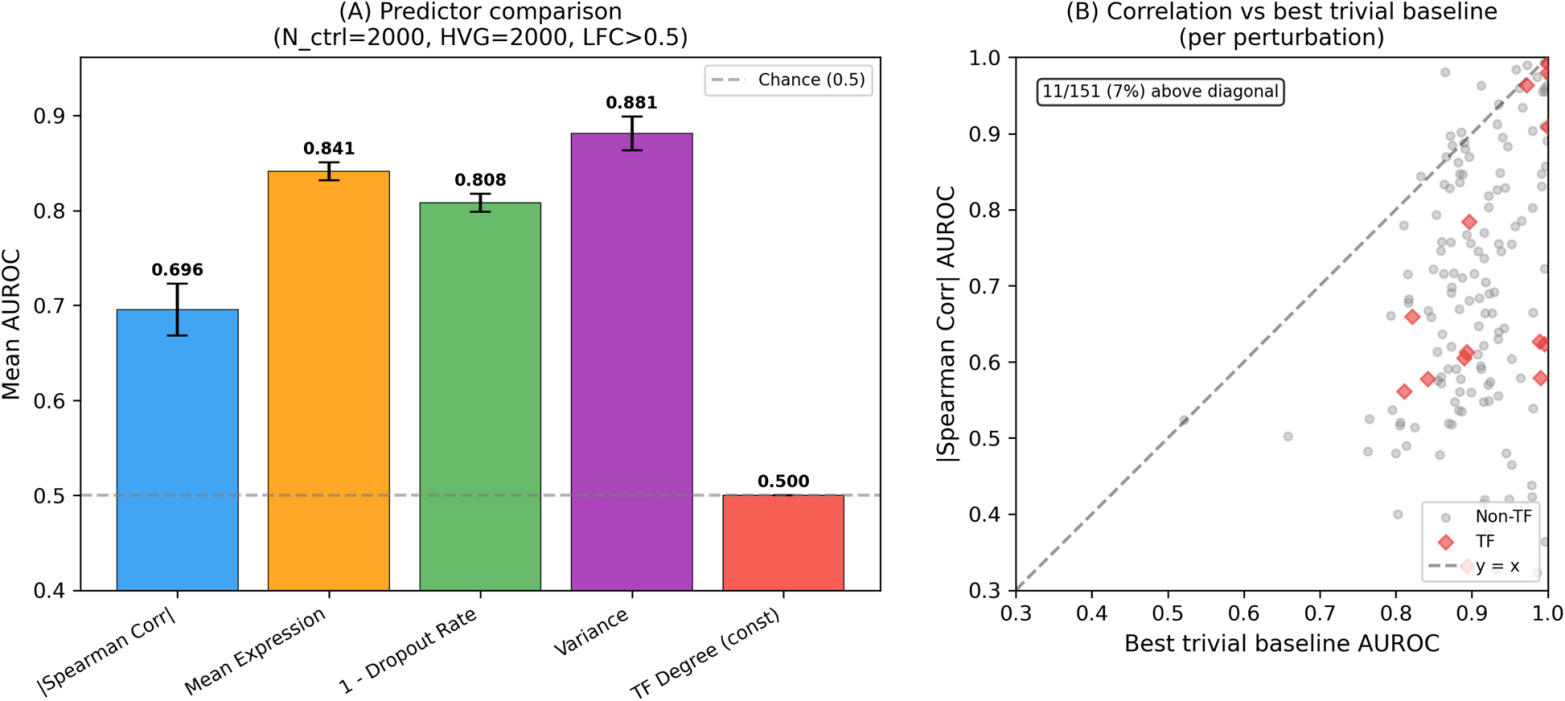
Gene-level baselines outperform pairwise edge scores. AUROC for predicting CRISPRi perturbation targets using gene-level features (variance, mean expression, dropout rate) versus correlation-based edge scores ($n = 151$ perturbations; attention comparison in text). All gene-level baselines significantly outperform both edge types ($p < 10^{-12}$)

**Fig. 4:**
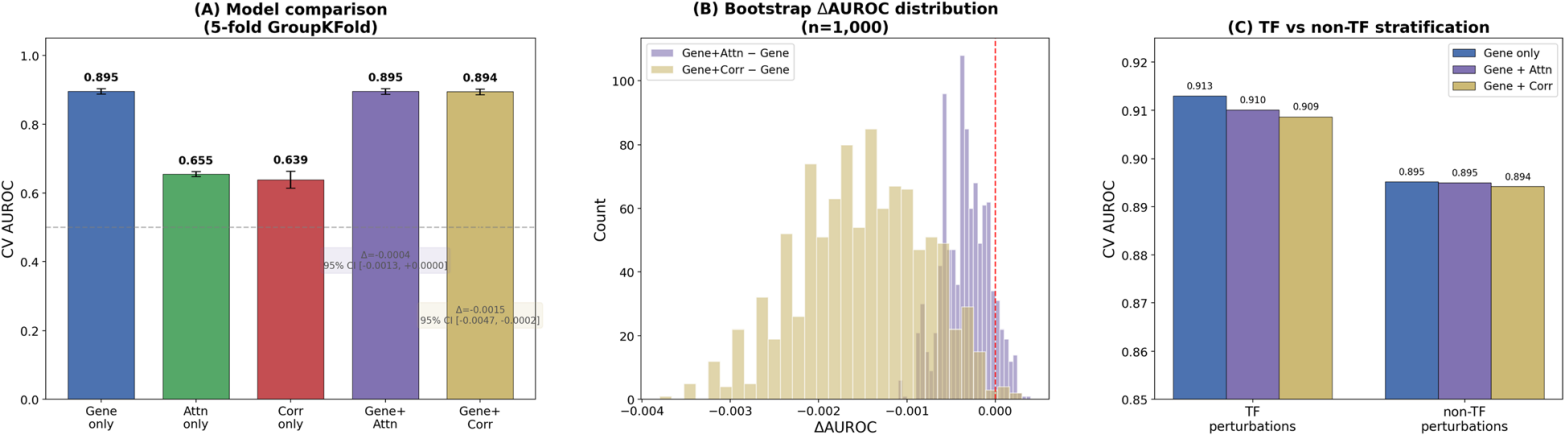
Pairwise edge scores provide no incremental value beyond gene-level features. **A** Cross-validated AUROC for five model configurations under GroupKFold by perturbation: gene-level features alone, attention only, correlation only, gene-level plus attention, and gene-level plus correlation. Adding pairwise edges provides no improvement. **B** Bootstrap $\Delta$AUROC distribution ($n = 100$) for gene+attention minus gene-only (purple) and gene+correlation minus gene-only (pink); both centred at zero. **C** Stratification by TF versus non-TF perturbation genes shows identical patterns

**Fig. 5:**
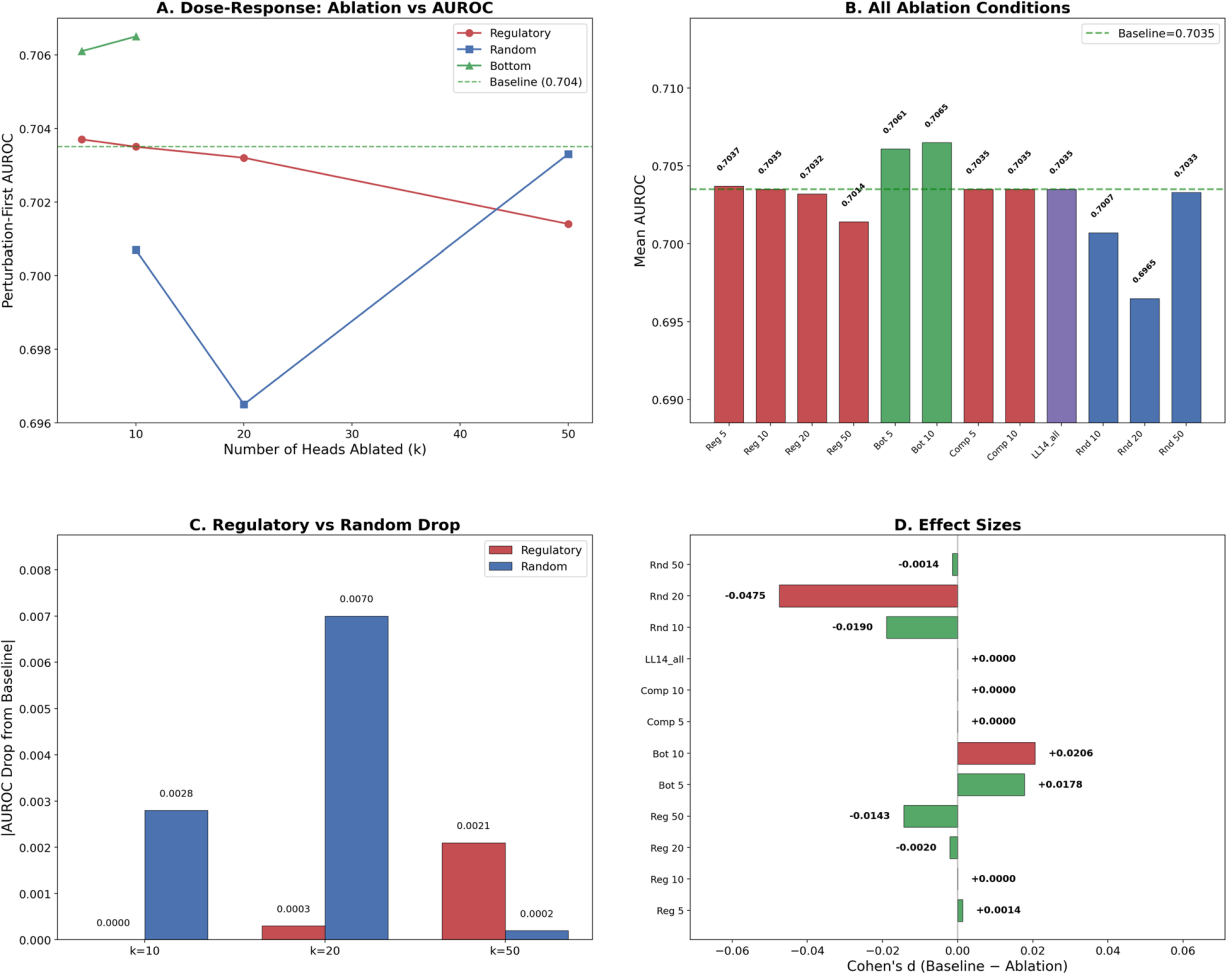
Causal ablation dose-response. **A** AUROC versus number of ablated heads for TRRUST-ranked (red), composite-ranked (green), and random (blue) ablation. Regulatory heads can be ablated up to $k = 50$ with no significant degradation, while random ablation causes significant drops. **B** All ablation conditions: mean AUROC across 13 conditions versus baseline. **C** Regulatory versus random drop magnitude at each dose level *k*. **D** Cohen’s *d* effect sizes (baseline minus ablation) for all conditions; most are indistinguishable from zero

**Fig. 6:**
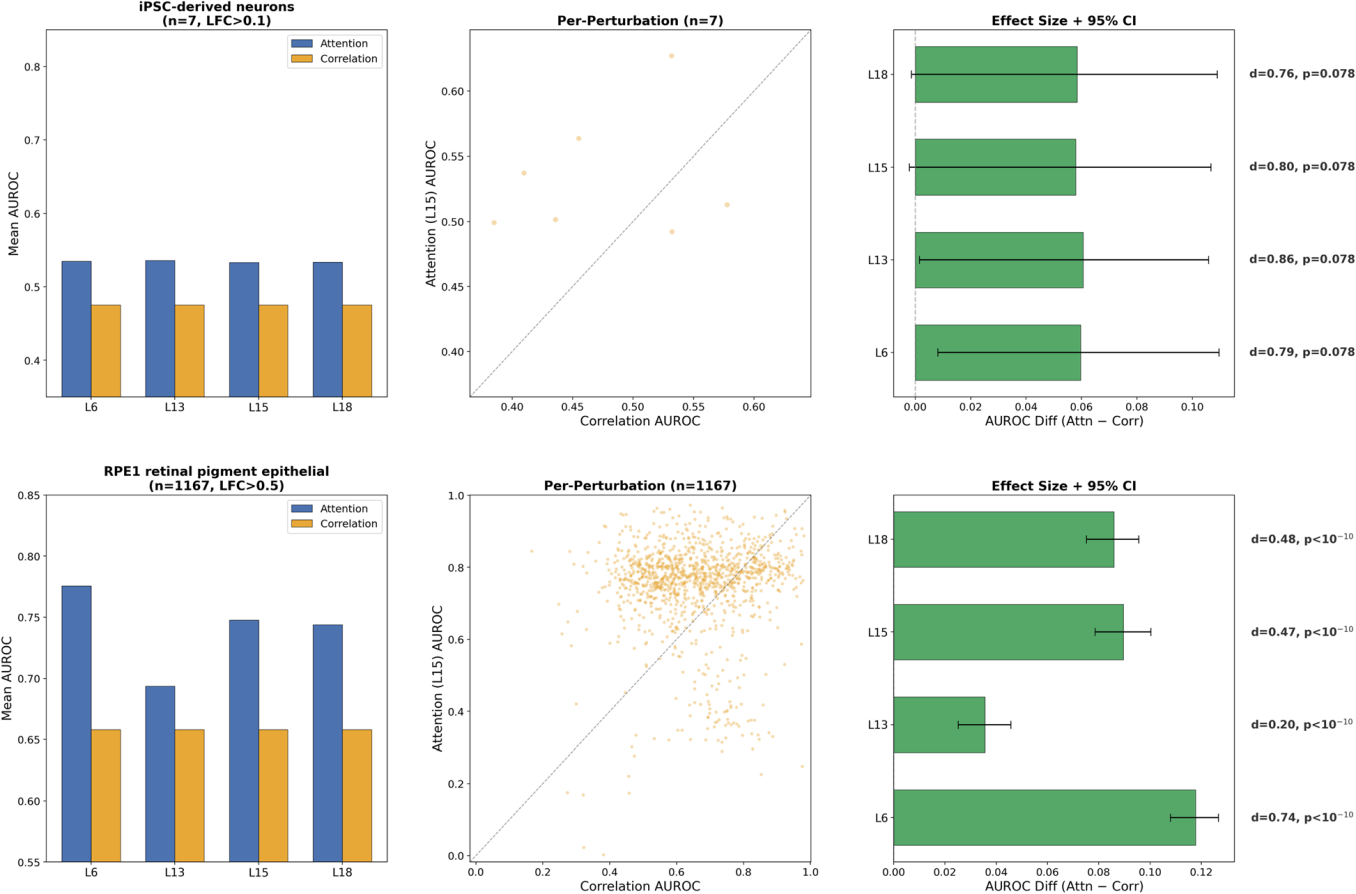
Context-dependent attention–correlation relationship. Perturbation-first AUROC comparison between Geneformer V2-316M attention and Spearman correlation across cell types. In RPE1 ($n = 1{,}251$), attention significantly outperforms correlation ($d = 0.47$, $p < 10^{-10}$), contrasting with K562 where they are indistinguishable. Sensitivity to LFC threshold shown for RPE1

**Fig. 7:**
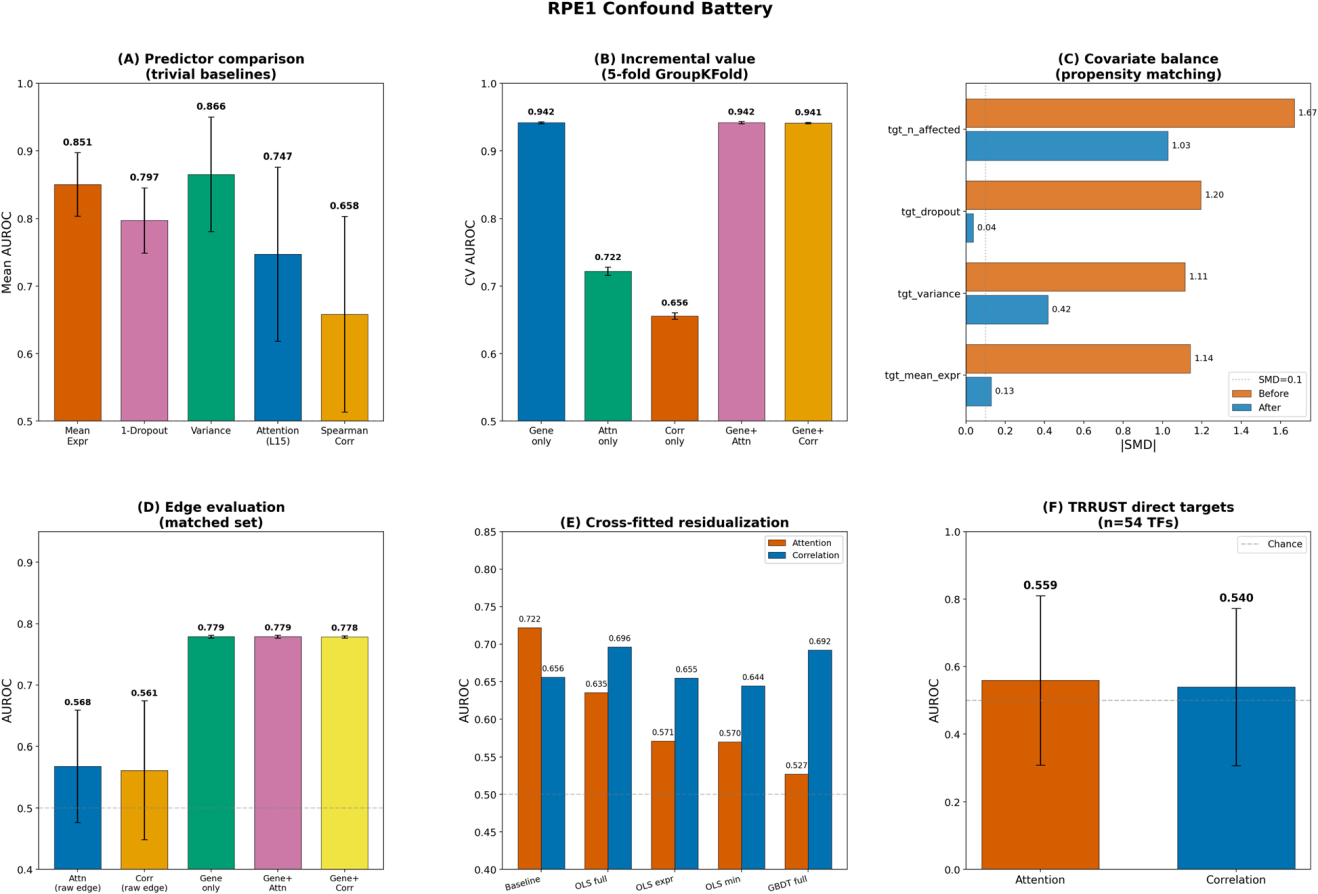
RPE1 confound battery confirms gene-level dominance. **A** Trivial baselines outperform both edge types. **B** Gene-only AUROC $= 0.942$; zero incremental value from edges. **C** Covariate balance after propensity matching. **D** Matched-set evaluation: edges near chance. **E** Residualisation: attention loses ${\sim }88\%$ of signal; correlation increases (suppressor). **F** TRRUST prediction near chance

Five interlocking test families operationalise Objective B, with additional tests addressing Objective A and cross-objective contamination (Figs. 2, 3, 4, 5, 6 and 7; Additional file 1: Supplementary Notes 1–16). (i) *Trivial-baseline comparison* tests whether pairwise edge scores outperform univariate gene-level features (variance, mean expression, dropout rate) that require no model—a necessary-but-not-sufficient condition for claiming pairwise regulatory signal on Objective B. (ii) *Conditional incremental-value testing* asks whether adding edge scores to gene-level features improves Objective B prediction under progressively harder generalisation protocols (cross-perturbation, cross-gene, joint splits) using both linear (logistic regression) and nonlinear (gradient-boosted) models, across AUROC, AUPRC, and top-*k* recall. (iii) *Expression residualisation and propensity matching* isolate edge-specific signal by removing gene-level confounds via two complementary statistical approaches. (iv) *Causal ablation with fidelity diagnostics* uses head masking, uniform attention replacement, and MLP ablation to test whether “regulatory” heads causally contribute to Objective B, with diagnostics confirming that interventions materially perturb internal representations. (v) *Cross-context replication* tests whether findings on (i)–(iv) generalise across cell types (K562, RPE1, primary T cells, iPSC neurons) and perturbation modalities (CRISPRi, CRISPRa). Each family addresses a distinct confound on Objective B; their convergence provides stronger evidence than any individual test. A *positive-control synthetic pipeline* together with a real-data per-TF positive control (Additional file 1: Supplementary Note 21; Fig. S40; Tables S15–S16) establishes that the Objective B evaluation is sensitive—it recovers planted pairwise signal when such signal exists in the data—mitigating the concern that null results could reflect pipeline limitations rather than absence of signal. A boundary-condition tier (cross-species transfer, pseudotime, batch leakage, calibration; Additional file 1: Supplementary Notes 7–10) characterises the broader evaluation landscape. We report both continuous AUROC and top-*K* F1 metrics throughout because they can diverge—continuous AUROC evaluates the full gene-pair ranking while top-*K* F1 evaluates only thresholded retrieval—and that divergence, when present, is itself a finding. Together, these yield thirty-seven analyses with 153 statistical tests under Benjamini-Hochberg FDR correction (Additional file 1: Supplementary Note 16).

### Attention-specific scaling failure and CSSI remedy

To test whether increasing dataset size improves interpretability, we analysed archived scGPT kidney scaling runs across three model tiers (small/medium/large), three seeds per tier, and three cell counts (200, 1,000, 3,000). Top-*K* F1 for attention-derived GRN recovery against TRRUST [14] degrades with cell count—the 200$\rightarrow$1,000 drop is negative in all 9 tier$\times$seed runs (sign test $p = 0.002$; Additional file 1: Fig. S1; Supplementary Note 1), with a continued trend at 1,000$\rightarrow$3,000 (7/9 runs, $p = 0.09$). This finding is metric-dependent: continuous-score AUROC (no top-*K* thresholding) improves monotonically ($0.86 \rightarrow 0.93$; 0/9 degrading), qualifying the original observation. Controlled-composition experiments using correlation-based edges on Tabula Sapiens kidney data (Additional file 1: Fig. S1) disentangle sample size from heterogeneity: with fixed composition AUROC is stable ($\rho = -0.05$, $p = 0.82$), and heterogeneity at fixed *N* actually *improves* recovery ($\rho _\text {heterogeneity} = +0.63$, $p = 10^{-4}$), confirming that the degradation is attention-specific rather than inherent to edge scoring.

As a constructive contribution, we introduce Cell-State Stratified Interpretability (CSSI), motivated by a formal dilution model predicting that pooled attention-derived edge scores degrade as $\rho _\text {pool} \approx (n_1/N)\rho _1 \rightarrow 0$ with increasing heterogeneity. CSSI computes edge scores within cell-state strata (Leiden clustering on *k*-NN graphs from model embeddings) before aggregation, controlling the heterogeneity that drives attention-specific dilution. On DLPFC brain scRNA-seq data, CSSI-max improves top-*K* TRRUST recovery up to $1.85\times$ (Fig. 2), with the optimum at intermediate *K* (5–7 strata). Extended null tests across $K \in \{2, \ldots , 20\}$ confirm no false-positive inflation under uninformative (random) strata, ensuring CSSI improvements reflect genuine signal recovery. Real-data layer/head analysis reveals recoverable reference-edge signal concentrated in late Geneformer layers (L13 AUROC $= 0.694$; Additional file 1: Supplementary Note 11), and *(exploratory)* cell-level bootstrap analysis shows that 7/18 individual TFs have robust edge-level signal (global AUROC 95% CI: [0.71, 0.77]; Additional file 1: Supplementary Note 19). Synthetic validation with known ground-truth networks corroborates these findings (Additional file 1: Supplementary Note 12). Multi-model analysis shows convergent near-random unstratified performance across both scGPT and Geneformer (Additional file 1: Fig. S2; Supplementary Note 13)—despite fundamentally different architectures (gene-token vs. rank-based tokenisation), training objectives, and model scales—confirming the failure is architecture-independent and reflecting a persistent limitation of unstratified approaches to attention-derived GRN inference.

### Perturbation-first validation reveals gene-level dominance

We evaluated whether edge scores predict perturbation outcomes using the Replogle genome-scale CRISPRi dataset [22] (>640,000 K562 cells, 2,024 perturbed genes). For each CRISPRi perturbation, we computed edge scores between the perturbed gene and all other genes, then evaluated AUROC for classifying differentially expressed targets. Correlation-based edges achieve AUROC $= 0.696$ under the primary parameterisation ($N_\text {ctrl} = 2{,}000$, HVG $= 2{,}000$, LFC $> 0.5$; $n = 151$ perturbations), with all 27 sensitivity conditions (varying control cell count, HVG count, and LFC threshold) yielding AUROC $= 0.62$–0.76 (all $p < 0.005$; Additional file 1: Fig. S7; Supplementary Note 6), indicating robust predictive signal for co-expression edges.

Direct evaluation of Geneformer V2-316M attention-derived edges under the same parameterisation yields AUROC $= 0.704$ at L13 ($n = 280$ evaluable perturbations), statistically indistinguishable from correlation (0.703; Wilcoxon $p = 0.73$; Additional file 1: Fig. S3). L13 was pre-specified as the primary layer based on independent data: the CSSI analysis on DLPFC brain tissue (497 cells; Additional file 1: Supplementary Note 11) identified L13 as the best-performing layer for TRRUST recovery (AUROC $= 0.694$), *before* any K562 perturbation data were examined. The fact that attention edges—which reflect learned cross-gene interactions across 30 million training cells—perform no better on Objective B than simple Spearman correlation computed on a single dataset is a striking finding, indicating that whatever regulatory structure Geneformer’s pretraining has embedded into attention patterns does not translate into a pairwise advantage on the perturbation-target prediction task. As a planned secondary analysis, the full 18-layer perturbation-first profile reveals a clear architectural gradient: early layers achieve AUROC 0.47–0.64, mid layers 0.60–0.71, and late layers 0.69–0.74. A strict nested cross-validation protocol—designed to guard against overfitting across 18 candidate layers—identifies L15 as the best layer (AUROC $= 0.743$, $p_\text {Bonf} = 0.017$, $d = 0.22$; Additional file 1: Fig. S5), but this advantage is small (only 161/280 perturbations favour attention) and reverses entirely in CRISPRa ($d = -0.56$, $p = 4.9 \times 10^{-5}$), establishing it as K562-CRISPRi-specific rather than a general property of the model.

Critically, trivial gene-level baselines—variance, mean expression, and dropout rate—all outperform both attention and correlation edges on Objective B (AUROC 0.81–0.88 vs. 0.70; all $p < 10^{-12}$; Fig. 3). Variance alone achieves AUROC $= 0.881$—substantially exceeding the best attention layer—indicating that much of the perturbation-predictive signal reflects univariate target-gene susceptibility rather than pairwise regulatory structure. This is consistent with the endpoint–object mismatch described in the Discussion: Objective B is dominated by which genes are easily detectable as DE (high-variance, high-expression genes) rather than by which edges are regulatory. The dominance of gene-level baselines therefore does not imply that attention edges lack regulatory information—attention edges do recover curated regulatory interactions on Objective A *(exploratory)* (Additional file 1: Supplementary Note 17)—but it does establish a necessary-but-not-sufficient baseline: any claimed pairwise regulatory signal must first exceed gene-level features on this task.

### No incremental pairwise value on the perturbation-target prediction task

A key Objective B question is whether pairwise edge scores—attention or correlation—add predictive value *beyond* gene-level features for identifying perturbation targets. We constructed a dataset of $280 \times 1{,}999$ perturbation–gene observations (559,720 pairs, 2.8% positive rate) and compared five logistic-regression models under 5-fold GroupKFold cross-validation: gene-level features only (mean expression, variance, dropout rate), attention edge only, correlation edge only, gene-level plus attention, and gene-level plus correlation. Gene-level features alone achieve AUROC $= 0.895$ [bootstrap 95% CI: 0.884, 0.905]; adding attention yields $\Delta$AUROC $= -0.0004$ [$-0.001$, 0.000] and adding correlation yields $\Delta$AUROC $= -0.002$ [$-0.005$, 0.000] (Fig. 4). Neither pairwise edge type provides incremental value on Objective B, and this is not a power issue: 559,720 observations provide >99% power to detect $\Delta$AUROC $= 0.005$.

To rule out that this null result reflects a specific analytical choice, we tested progressively harder generalisation protocols—cross-gene splits (GroupKFold by target gene, preventing gene-level propensity leakage), joint cross-gene $\times$ cross-perturbation splits—and both linear (logistic regression) and nonlinear (GBDT) models across AUROC, AUPRC, and top-*k* recall. Under all tested combinations, the null persists: even the largest observed $\Delta$AUPRC ($+0.009$ under joint splits with GBDT) represents less than 4% relative improvement (Additional file 1: Fig. S6; Supplementary Note 15).

Cross-fitted residualisation reveals an important asymmetry on the Objective A evaluation: on K562 data, attention edges lose $\sim$76% of their above-chance TRRUST signal under expression-covariate residualisation (AUROC $0.66 \rightarrow 0.54$; OLS $R^2 = 0.03$, GBDT $R^2 = 0.23$), while correlation edges retain $\sim$91% ($0.63 \rightarrow 0.62$; Additional file 1: Fig. S4), indicating that attention-derived edge scores are substantially more expression-confounded than correlation edges at the pairwise level. Degree-preserving null models show that much of the global AUROC reflects TF degree structure (degree-null AUROC 0.69 vs. observed 0.76; Additional file 1: Supplementary Note 14). A propensity-matched benchmark provides a direct test: after matching each DE-positive target to $k = 5$ DE-negative targets with similar expression mean, variance, and dropout rate via propensity-score nearest-neighbour matching ($n_\text {matched} = 59{,}153$ pairs; positive rate $26.5\%$ vs. $2.8\%$ before matching), attention edges retain modest raw discriminability (AUROC $= 0.609$) but add zero incremental value over gene-level features ($\Delta$AUROC $= -0.000$; 95% CI: $[-0.000, +0.000]$; Additional file 1: Fig. S8).

### Causal ablation reveals distributed redundancy

The preceding analyses are observational. To provide causal evidence, we used BERT’s head-mask parameter to zero out specific attention heads during Geneformer V2-316M’s forward pass and measured the effect on perturbation-first AUROC (2,000 K562 control cells, 280 perturbations per condition). We ranked all 324 heads ($18 \times 18$) by TRRUST-recovery AUROC and tested 13 ablation conditions spanning dose-response, alternative rankings, full-layer ablation, inverse controls, and matched random controls (Fig. 5). The dose-response curve is unambiguous: ablating the top-5 (0.704, $p = 0.24$), top-10 (0.704, $p = 0.94$), top-20 (0.703, $p = 0.60$), or top-50 TRRUST-ranked heads (0.701, $p = 0.10$; 15% of all heads) produces no significant degradation from baseline (0.704). By contrast, ablating 20 random heads causes a significant drop (0.697, $d = 0.33$, $p < 10^{-8}$), demonstrating that “regulatory” heads are *less* causally important than random heads. An alternative ranking by composite score (TRRUST AUROC $\times$ layer perturbation-first AUROC) yields identical results. Ablating all 18 heads in L14 (the best perturbation-first layer) produces exactly baseline AUROC, and ablating the bottom-5 heads actually *improves* performance (0.706, $p = 0.005$).

Two families of orthogonal interventions corroborate this null: uniform attention replacement (destroying attention patterns while preserving head output magnitude) on TRRUST-ranked heads has no effect (top-5: 0.704, top-10: 0.703), and MLP pathway ablation (zeroing FFN output) at L15 and L13–L15 both produce exactly 0.704 (Additional file 1: Fig. S9). Intervention-fidelity diagnostics confirm that all six interventions materially perturb internal representations (max hidden-state cosine distance 0.023–0.190), with TRRUST-ranked heads producing $23\times$ larger logit perturbation than random heads at matched dose (Additional file 1: Fig. S9; Supplementary Note 14), ruling out the possibility that behavioral nulls reflect ineffective interventions. The convergence of null results across three qualitatively different intervention channels—head masking, attention pattern destruction, and MLP ablation—provides substantially stronger evidence than single-method ablation alone and makes it unlikely that concentrated regulatory signal exists in any head subset. This distributed-redundancy pattern contrasts sharply with NLP findings where specific attention heads encode identifiable syntactic or semantic functions [23, 24]; in single-cell models on Objective B, perturbation-predictive computation appears to reside in the value/FFN pathway rather than in learnable attention patterns. Per-head ranking across all 324 heads shows substantial variation in TRRUST recovery (top-5 AUROC $= 0.70$–0.75 vs. population mean $= 0.58 \pm 0.07$), yet this variation does not translate to causal importance for perturbation prediction, consistent with the signal being distributed across the entire network rather than concentrated in identifiable “regulatory circuits.”

Cross-architecture replication in scGPT. To test whether the distributed-redundancy pattern is Geneformer-specific or generalises across architectures, we extended the ablation to scGPT via two complementary intervention mechanisms (Additional file 1: Supplementary Note 22; Tables S17–S18). The first is an edge-level intervention on cached scGPT per-head attention from three Tabula Sapiens tissue subsets (immune, kidney, brain; 1,200-gene shared HVG vocabulary; 12 layers $\times$ 8 heads $=$ 96 heads), in which pooled TRRUST AUROC is recomputed over only the non-ablated heads under the same 13-condition battery; baseline pooled AUROC is 0.706–0.708, ablating the top-50 TRRUST-ranked heads produces $\Delta$AUROC of $-0.002$ to $-0.003$, ablating the bottom-5 heads slightly *improves* pooled AUROC, and random ablation is null—identical pattern across all three tissues. The second is a true forward-pass intervention via PyTorch hooks on the scGPT whole-human checkpoint: for each ablated (*L*, *h*) we zero the per-head column slice of layer *L*’s out_proj.weight, which is mathematically equivalent to zeroing head *h*’s output before the projection and propagates through the residual stream into every subsequent layer’s attention pattern. Forward-pass baselines on 100 cells per tissue are 0.799 (immune), 0.795 (kidney), and 0.781 (brain). The diagnostic comparison is reg. top-20 vs. random-20 ablation at matched dose: in immune $\Delta _\text {top-20} = -0.039$ vs. $\Delta _\text {random-20} = -0.063 \pm 0.041$; in kidney $-0.046$ vs. $-0.077 \pm 0.054$; in brain $-0.101$ vs. $-0.067 \pm 0.045$. In all three tissues the difference $|\Delta _\text {top-20} - \Delta _\text {random-20}|$ is within 0.8 standard deviations of the random-seed noise, and the sign of the difference even varies across tissues. If top-TRRUST-ranked heads were preferential loci of regulatory computation, top-20 ablation would consistently produce larger drops than random-20 across tissues; it does not. This is the operational definition of distributed redundancy and matches the Geneformer head-mask pattern. The convergence of distributed-redundancy across two architecturally distinct models, two intervention mechanisms (edge-level and forward-pass), three tissues (immune, kidney, brain), and two evaluation objectives (Objective A and Objective B) indicates the pattern is a general property of how transformer attention encodes biological structure in single-cell foundation models, not a Geneformer-specific, task-specific, tissue-specific, or intervention-mechanism-specific artefact.

### Context-dependent attention–correlation relationship

Cross-context replication across four cell types and two perturbation modalities reveals that the attention–correlation relationship is genuinely context-dependent. On K562 CRISPRi ($n = 280$), Geneformer V2-316M attention and Spearman correlation are statistically indistinguishable (L13: 0.704 vs. 0.703, $p = 0.73$). On K562 CRISPRa [25] ($n = 77$), attention significantly *underperforms* correlation (AUROC 0.55 vs. 0.65; $p < 10^{-6}$), with correlation outperforming attention in 55–63 of 77 perturbations. However, the first adequately powered non-K562 replication tells a qualitatively different story: on RPE1 retinal epithelial cells [22] ($n = 1{,}251$ evaluable perturbations; minimum detectable $d = 0.079$), attention significantly *outperforms* correlation at all layers (L15: 0.748 vs. 0.658, $d = 0.47$, $p < 10^{-10}$; L6: 0.776, $d = 0.74$; Fig. 6). The advantage strengthens under stringent DE thresholds (LFC $> 0.5$: $+0.090$; LFC $> 0.25$: $+0.049$). iPSC-derived neurons ($n = 7$) trend in the same direction ($d = 0.80$, $p = 0.078$), while primary T cells [26] ($n = 7$) show no difference ($p = 0.81$; Additional file 1: Supplementary Note 14). A methodological asymmetry—RPE1 includes perturbation genes forced into the HVG set (3,309 vs. 2,000 genes in K562)—could confound this comparison, but restricting RPE1 to the top 2,000 HVGs (matching K562 protocol) *increases* the attention advantage ($\Delta = +0.168$, $n = 418$; $p < 10^{-46}$), as correlation degrades more than attention when the evaluation universe shrinks. Bootstrap 95% CIs on the per-perturbation attention advantage exclude zero for both K562 ($\Delta = +0.060$ $[+0.040, +0.080]$) and RPE1 ($\Delta = +0.090$ $[+0.079, +0.101]$; Additional file 1: Supplementary Note 14). The cross-context pattern—equal in K562 CRISPRi, worse in CRISPRa, better in RPE1, and trending better in neurons—indicates that the attention–correlation relationship depends on cell type and perturbation modality rather than reflecting a universal architectural limitation or advantage. This context-dependence may arise from cell-type-specific regulatory architectures, differences in perturbation effect sizes, or variation in the relationship between expression rank (Geneformer’s input) and regulatory importance across cell types.

### Cross-cell-type generalisation of confound pattern

To test whether the RPE1 attention advantage reflects genuine Objective B regulatory signal or gene-level confounds, we applied the full K562 confound-decomposition battery to the RPE1 dataset ($n = 1{,}167$ evaluated perturbations, 3, 838, 263 gene-pair observations; Fig. 7). Five analyses converge on the same Objective B conclusion as K562. (i) Trivial baselines outperform both edge types (variance AUROC $= 0.866$, mean expression $= 0.851$, dropout $= 0.797$ vs. attention $= 0.747$ and correlation $= 0.658$; all paired $p < 10^{-38}$). (ii) Gene-level features alone achieve AUROC $= 0.942$ under 5-fold GroupKFold CV; adding attention yields $\Delta$AUROC $= +0.0001$ [95% CI: $+0.0001$, $+0.0001$]—functionally zero—while adding correlation yields $\Delta$AUROC $= -0.0006$. (iii) Propensity-matched edges drop to near chance (attention $= 0.568$, correlation $= 0.561$) with LR incremental value exactly zero. (iv) GBDT residualisation removes $\sim$88% of attention’s above-chance signal (AUROC $0.722 \rightarrow 0.527$) while correlation signal *increases* ($0.656 \rightarrow 0.692$), indicating a suppressor effect. (v) TRRUST direct-target prediction is near chance on Objective A in this setting ($n = 54$ evaluable TFs; attention $= 0.559 \pm 0.250$, correlation $= 0.540 \pm 0.233$).

Together, these five analyses establish that the RPE1 attention advantage over correlation (diff $= +0.089$, $p < 10^{-10}$) does not reflect attention capturing regulatory structure that correlation misses on Objective B; rather, attention is a better proxy for the gene-level susceptibility features that dominate the perturbation-target prediction task. The RPE1 gene-only AUROC (0.942) exceeds the K562 value (0.895), consistent with RPE1’s larger gene universe (3, 290 vs. 2, 000 genes) providing richer gene-level features. The confound-decomposition conclusion—pairwise edge scores carry no incremental information beyond univariate gene properties on Objective B—generalises across both cell types where adequate power is available, establishing this as a robust finding rather than a K562-specific artifact. It does not generalise to Objective A; as Supplementary Note 17 shows, attention edges do recover layer-specific regulatory structure on curated-reference benchmarks.

### Positive control: the pipeline detects planted regulatory signal

A natural objection to the Objective B null is that the pipeline might not be sensitive enough to detect pairwise regulatory signal even when it exists. We address this with two positive controls (Additional file 1: Supplementary Note 21; Fig. S40; Tables S15–S16).

Synthetic positive control. We simulated a single-cell Perturb-seq dataset with a planted hierarchical GRN (300 genes; 20 TFs; 6–8 direct targets per TF; mixed activation/repression coefficients; 5,000 control cells; 20 perturbation conditions; heavy-tailed TF activities; Gaussian noise; 10% dropout). Per-gene expression was z-scored post-hoc so univariate variance is uninformative about ground-truth target status, isolating pairwise signal from univariate baselines. Three stress variants were tested: (A) clean strong direct edges with minimal indirect propagation; (B) weak direct edges with strong indirect propagation; (C) balanced regime. For each variant, we additionally ran a shuffled-null control (ground-truth target labels permuted within each TF’s regulon while keeping expression unchanged). Under planted ground truth, the full incremental-value pipeline detects pairwise signal cleanly: $\Delta$AUROC of $+0.518$ [bootstrap 95% CI $+0.496$, $+0.544$] in variant A, $+0.452$ [$+0.425$, $+0.475$] in variant B (the worst case for indirect propagation), and $+0.492$ [$+0.463$, $+0.530$] in variant C. Under shuffled ground truth, $\Delta$AUROC is near zero ($-0.04$ to $+0.06$) with no consistent direction. The pipeline is sensitive: when planted pairwise signal exists, it is detected with high confidence, and indirect propagation does not mask it.

Real-data positive control. We complemented the synthetic test by computing per-TF AUROC for Geneformer V2-316M attention edges discriminating TRRUST direction-known direct targets from non-target HVG genes, restricted to TFs with $\ge 4$ HVG-evaluable targets ($n = 9$ TFs from 116 HVG-vocabulary TRRUST entries). At Geneformer’s late layers, the mean per-TF AUROC is meaningfully above chance with bootstrap 95% CIs that exclude 0.5: L13 $= 0.654$ [0.594, 0.717] (9/9 TFs above chance), L17 $= 0.672$ [0.610, 0.745] (9/9 above chance, best TF TFDP1 at 0.854). The same attention edges that score zero incremental value on Objective B (perturbation-target prediction) recover direct regulatory targets on Objective A. This is the empirical signature of the endpoint–object mismatch described in the Discussion: attention encodes direct-regulatory information that surfaces on the Objective A task but is dominated by gene-level susceptibility on the Objective B task.

Together, the two positive controls rule out pipeline insensitivity as an explanation for the Objective B null. The most plausible interpretation is that pairwise attention edges genuinely do not add information beyond gene-level features for predicting the integrated DE-hit endpoint, even though they do encode some direct-regulatory structure that is recoverable on Objective A.

## Discussion

### Two complementary contributions

Our results support a two-part conclusion, and we state it explicitly because the two parts are not contradictory but they are often elided when attention interpretability is discussed.

*A cautionary Objective-B finding.* For the task of predicting which genes will show differential expression after a CRISPR perturbation, attention-derived edge scores in scGPT and Geneformer add no incremental value over trivial gene-level features. This rests on convergent evidence: trivial-baseline comparison, incremental-value testing under three generalisation protocols with linear and nonlinear models, expression residualisation, propensity-score matching, and causal ablation of TRRUST-ranked heads via three independent intervention channels (head masking, uniform attention replacement, MLP ablation) all point to the same conclusion. The two positive controls—synthetic and real-data (Additional file 1: Supplementary Note 21; Fig. S40; Tables S15–S16)—confirm that the Objective-B pipeline is sensitive: it detects planted pairwise regulatory signal when such signal exists, ruling out pipeline insensitivity as an explanation for the null.

*A constructive Objective-A finding.* Attention patterns do encode biologically structured information, and that structure is not incidental. Layer-specific organisation is clear and robust *(exploratory; layer-by-database profile beyond the pre-specified TRRUST-recovery test)*: STRING protein–protein interactions are concentrated in early layers (AUROC $=0.64$ at L0, decreasing with depth, $\rho = -0.61$, $q_\text {BH} = 0.01$; Additional file 1: Supplementary Note 17), while TRRUST transcriptional regulation peaks in late layers (AUROC $=0.75$ at L15; $\rho = +0.51$, $q_\text {BH} = 0.03$). This hierarchy survives pairwise expression residualisation—97% of the TRRUST signal is retained after controlling for Spearman expression similarity—indicating that late-layer attention captures regulatory structure beyond co-expression. Cell-State Stratified Interpretability (CSSI) exploits this organisation by computing edge scores within cell-state strata before aggregation, yielding up to $1.85\times$ improvement in curated GRN recovery on heterogeneous data with null-control tests confirming no false-positive inflation. CSSI is therefore a deployable method that extracts the structure attention does encode.

### Endpoint–object mismatch: why Objectives A and B disagree

The cautionary and constructive findings coexist because the two objectives measure different biological quantities. Objective A is an edge-identification task: it asks whether a particular TF–target pair is in the top of a ranked list. Objective B is an effect-magnitude task: it asks whether perturbing a source gene produces detectable differential expression at a target. These tasks are sensitive to different properties.

Objective B is dominated by target *susceptibility*—high-variance, high-expression genes are more easily detected as DE regardless of whether the perturbation acts on them directly or through intermediate regulators. This is why gene-level variance alone achieves Objective B AUROC of 0.88–0.94 (K562 and RPE1), and why incremental-value tests find zero contribution from pairwise edges. A genuine direct regulatory edge that buffers or feeds back negatively can produce minimal DE despite being real; conversely, a non-regulatory pairwise feature that happens to correlate with target susceptibility can contribute to Objective B. Our null result therefore rules out that attention edges add value on *this specific task*—it does not rule out that attention encodes sparse direct-target signal too small to move the integrated Objective-B endpoint (Additional file 1: Supplementary Note 20). Practitioners using attention edges to generate regulatory hypotheses for experimental follow-up should test Objective A with CSSI; practitioners using foundation models to predict perturbation response should not rely on pairwise attention edges as features.

### Scope of CSSI

CSSI is a method for Objective A. It improves curated-reference GRN recovery under heterogeneous cell populations by controlling the state-mixing that dilutes pooled edge scores. It does *not* close the Objective-B gap: stratified attention edges remain dominated by gene-level features on perturbation prediction. Practical guidance: use CSSI when you need attention-derived regulatory hypotheses to hand off to downstream experimental validation (Objective A); do not expect CSSI to improve perturbation-response prediction. This scope boundary is informative, not a weakness: it sharpens what attention interpretability can and cannot contribute.

### Cross-context pattern

The attention–correlation relationship is genuinely context-dependent: K562 CRISPRi (equal), K562 CRISPRa (attention worse), RPE1 (attention better; $d = 0.47$, $p < 10^{-10}$), T cells and iPSC neurons (trending but underpowered). However, this context-dependence is superficial on Objective B. In both K562 and RPE1—the two cell types with adequate power—the full confound-decomposition battery reveals the same underlying pattern: gene-level features dominate perturbation prediction (AUROC 0.895–0.942), and pairwise edge scores add zero incremental value. The RPE1 attention advantage reflects attention being a better proxy for gene-level confounds—not attention capturing regulatory structure that correlation misses. Raw performance comparisons between edge-scoring methods are therefore misleading without confound controls; we recommend that such controls become standard practice.

### Relation to attention-as-explanation in NLP, and scope of our ablation evidence

These findings extend the attention-as-explanation debate from NLP [17, 19, 27] to biological foundation models. In language models, attention weights do not reliably indicate feature importance [18]; our results demonstrate an analogous limitation in single-cell models on the specific task of perturbation-target prediction. Observational Objective-B findings (no incremental pairwise value, gene-level dominance, RPE1 replication, CRISPRa reversal) are established across both scGPT and Geneformer architectures where testable. Causal ablation evidence is reported for both Geneformer V2-316M (via the BERT head_mask interface) and scGPT (via a model-agnostic PyTorch hook-based intervention introduced in this work; see Methods and Additional file 1: Supplementary Note 22, Tables S17–S18), and both architectures produce the same null dose-response to ablation of TRRUST-ranked heads, strengthening the distributed-redundancy interpretation beyond a single-model result. This distributed pattern contrasts sharply with NLP findings where specific attention heads encode identifiable syntactic or semantic functions [23, 24]: in single-cell models on Objective B, perturbation-predictive computation appears to reside in the value/FFN pathway rather than in learnable attention patterns.

Crucially, these findings characterise specific model components—attention weights, individual heads, and MLP blocks at particular layers—and specific tasks (Objective B), not the full model’s predictive capacity on all tasks. Transformer-based foundation models integrate information through residual streams, layer normalisations, and feed-forward networks that collectively contain far more parameters than attention alone. A model can be a capable black-box predictor even when its attention patterns do not serve as interpretable readouts for this particular task. The value of systematic component-level evaluation is not to establish fundamental limits of these architectures, but to narrow the search for where biologically meaningful representations reside: by ruling out attention as the locus of Objective-B computation, future interpretability work can focus on residual-stream geometry, MLP-stored knowledge, or representations that emerge only from the full forward pass.

### Limitations

Several limitations constrain interpretation. Perturbation data span four cell types and two modalities, but additional tissues, species, and architectures (scFoundation [4], UCE, CellPLM) remain untested; we selected scGPT and Geneformer because they are the two most widely cited single-cell foundation models with public checkpoints and cover the two dominant tokenisation strategies (gene-token vs. rank-based), and because newer architectures are benchmarked against them. Conclusions are likely to generalise to similar transformer architectures but this remains to be tested. The synthetic and real-data positive controls (Additional file 1: Supplementary Note 21; Fig. S40; Tables S15–S16) establish pipeline sensitivity on planted ground-truth data and on TRRUST direction-known TFs respectively, and several internal calibrations provide additional evidence against endpoint insensitivity: the pipeline exhibits wide dynamic range (gene-level AUROC 0.88–0.94, so the incremental-value null is specifically about pairwise structure, not endpoint saturation); residualisation produces asymmetric results (attention loses $76\%$ of signal vs. $9\%$ for correlation), distinguishing differently confounded scores; the per-TF bootstrap detects above-chance pairwise signal in 7/18 TFs on Objective A (Additional file 1: Supplementary Note 14); and the pipeline detects real cross-context differences (CRISPRa reversal, RPE1 $\ne$ K562), ruling out a floor effect. Reference database circularity affects all GRN evaluation; however, restricting TRRUST to direction-known entries (Activation/Repression; 58% of pairs) yields virtually identical per-TF AUROC (mean 0.682 vs. 0.692; median improves to 0.695; Additional file 1: Supplementary Note 14), confirming conclusions are not driven by circularly validated entries. Perturb-seq itself is an imperfect regulatory ground truth because differential expression after a knockdown integrates direct regulation with indirect propagation and target susceptibility (Additional file 1: Supplementary Note 20)—the Objective A/B distinction above is partly motivated by this limitation. Boundary-condition analyses (Additional file 1: Supplementary Notes 7–10) use correlation-based edges and characterise the evaluation landscape broadly.

## Conclusions

We recommend that practitioners: (i) apply trivial-baseline and incremental-value tests before claiming pairwise regulatory signal; (ii) report both thresholded (top-*K*) and continuous (AUROC) metrics; (iii) apply CSSI or equivalent stratification for heterogeneous populations; and (iv) validate causal claims with ablation controls accompanied by intervention-fidelity diagnostics. Three directions are most promising: intervention-aware pretraining on perturbation data [22] could embed causal rather than correlational structure; hybrid architectures using foundation model embeddings as inputs to GRN inference modules could combine representational power with regulatory inductive biases; and CSSI-enhanced pipelines with conformal prediction sets [28] provide an immediately deployable framework. The gap between recoverability and perturbation-outcome predictive validity remains the central challenge for the field.

The evaluation framework presented here—thirty-seven analyses with 153 statistical tests under Benjamini-Hochberg FDR correction, spanning observational, interventional, and cross-context evidence—establishes reusable quality-control standards for mechanistic interpretability claims in single-cell foundation models. By demonstrating that attention-derived edge scores add no incremental value over gene-level features for perturbation-target prediction, while also encoding biologically structured signal that CSSI can exploit for Objective-A curated-reference recovery, these results redirect the search for biologically meaningful representations toward residual-stream geometry, MLP-stored knowledge, and full-model representations, while providing CSSI as an immediately practical tool for improved Objective-A edge recovery.

## Methods

### Models and data

We analyse scGPT [1] and Geneformer [2]: scGPT uses gene-token transformers with attention-derived edge scores extracted from all layers/heads; Geneformer uses BERT-style rank-based tokenisation. These two models were selected because they are the most widely cited single-cell foundation models with public checkpoints, they cover the two dominant tokenisation strategies (gene-token vs. rank-based), and they are the reference architectures against which newer single-cell foundation models (scFoundation [4], UCE, CellPLM) are routinely benchmarked. Conclusions established here are likely to generalise to similar transformer architectures but extension to those models remains for future work.

We use Geneformer V1-10M (6 layers, 4 heads) for multi-model comparison and Geneformer V2-316M (18 layers, 18 heads) as the primary model for all layer-level mechanistic analyses (perturbation-first AUROC, head ablation, biological characterisation). Geneformer V2-316M is loaded from HuggingFace (ctheodoris/Geneformer, subfolder Geneformer-V2-316M) and instantiated with attn_implementation="eager" to expose the BERT head_mask interface used for causal ablation. The scGPT whole-human checkpoint (12 layers, 8 heads) is loaded with use_fast_transformer=False to expose standard PyTorch nn.MultiheadAttention modules suitable for hook-based intervention (used in the cross-architecture ablation extension; Additional file 1: Supplementary Note 22).

Datasets include Tabula Sapiens atlas tissues [29] (immune, kidney, lung), DLPFC brain scRNA-seq [30], Replogle genome-scale CRISPRi [22] (K562 and RPE1), Adamson CRISPRa [25], Shifrut T-cell CRISPRi [26], and Tian iPSC neurons. Preprocessing follows standard Scanpy quality control [31]: gene filtering ($\ge 3$ cells expressing), cell filtering ($\ge 200$ genes detected), normalisation to $10^4$ counts per cell, $\log _{1+x}$ transform, and HVG selection by variance.

### Attention extraction and edge score computation

For Geneformer V2-316M, attention matrices are extracted via PyTorch forward hooks registered on each BertSelfAttention module rather than using the model’s output_attentions=True flag, which is memory-prohibitive at $\sim$10.8 GB per cell when extracting from all 18 layers simultaneously. Hooks capture the post-softmax attention weights of shape $(n_\text {heads}, n_\text {tokens}, n_\text {tokens})$ before they are passed to the value projection, allowing extraction of all 18 layers and all 18 heads in a single forward pass at fixed memory cost. Batch size is fixed at 1 because Geneformer’s tokenisation produces variable-length sequences and the hook-based extraction does not benefit from batching. Per-cell attention is averaged across heads within each layer, then averaged across cells to produce per-(layer, source-gene, target-gene) edge scores. The primary evaluation layer (L13) was pre-specified based on independent DLPFC brain data showing that L13 maximises TRRUST recovery AUROC (Additional file 1: Supplementary Note 11), *before* any K562 perturbation data were examined.

For scGPT, attention is extracted via two routes. Route A reads from cached per-(layer, head, source-gene, target-gene) matrices generated by a separate extraction pipeline using forward hooks on the standard PyTorch attention modules; Route B runs scGPT live with PyTorch hooks for forward-pass head ablation. Per-head ablation in scGPT (Additional file 1: Supplementary Note 22) is implemented in two complementary ways: (i) edge-level ablation on cached matrices, in which the pooled edge score is recomputed across only the non-ablated heads (a first-order approximation that does not propagate through the residual stream); and (ii) true forward-pass ablation via a model-agnostic intervention that zeros the column slice of each layer’s out_proj.weight corresponding to the ablated head, equivalent to zeroing the per-token head output before the projection (and propagating through all subsequent layers). Forward-pass ablation requires disabling nn.TransformerEncoder’s nested-tensor fastpath because (i) it is incompatible with the need_weights=True hook used to capture per-head attention weights and (ii) it calls aten::_nested_tensor_from_mask_left_aligned which is not implemented on Apple Silicon MPS. The out_proj weight-zeroing trick is necessary because nn.MultiheadAttention dispatches to F.multi_head_attention_forward, which bypasses hooks on the out_proj submodule. Both intervention mechanisms yield the same qualitative conclusion (Supplementary Note 22).

### Model coverage by analysis family

Table 1 summarises which architectures contributed to each analysis family. Observational findings (trivial baselines, incremental value, residualisation, propensity matching, cross-context replication, attention–correlation comparison) are established across both scGPT and Geneformer wherever both architectures provide sufficient signal. Causal ablation evidence is reported for both architectures: the main-text head-mask ablation uses Geneformer V2-316M’s BERT head_mask interface, and a two-pronged scGPT ablation (Additional file 1: Supplementary Note 22; Tables S17–S18) replicates the distributed-redundancy pattern via both edge-level intervention on cached attention and a true forward-pass intervention via PyTorch hooks on the live model. CSSI was developed and validated primarily on scGPT scaling runs and Geneformer attention; cross-architecture validation is in Additional file 1: Supplementary Note 13.

**Table 1:** Per-analysis model coverage. Rows are analysis families; columns indicate which models were tested. “GF V2” = Geneformer V2-316M (18 $\times$ 18 = 324 heads); “GF V1” = Geneformer V1-10M (6 $\times$ 4 = 24 heads); “scGPT” = scGPT whole-human checkpoint (12 $\times$ 8 = 96 heads). $\checkmark$ indicates the analysis was performed for that model; – indicates not applicable or not run

Analysis family	GF V2-316M	GF V1-10M	scGPT
Scaling failure (Objective A)	–	–	$\checkmark$
CSSI development & null tests	$\checkmark$	$\checkmark$	$\checkmark$
Multi-model GRN recovery	$\checkmark$	$\checkmark$	$\checkmark$
Trivial-baseline comparison (Obj. B)	$\checkmark$	–	$\checkmark$
Incremental value (Obj. B)	$\checkmark$	–	$\checkmark$
Expression residualisation (Obj. A)	$\checkmark$	–	$\checkmark$
Propensity matching (Obj. B)	$\checkmark$	–	–
Per-head TRRUST ranking	$\checkmark$	–	$\checkmark$
Causal head ablation (forward pass)	$\checkmark$	–	–
Causal head ablation (edge-level, Supp. 22)	–	–	$\checkmark$
Orthogonal interventions (uniform attn., MLP)	$\checkmark$	–	–
Intervention-fidelity diagnostics	$\checkmark$	–	–
Cross-context (RPE1, T cells, neurons)	$\checkmark$	–	–
Biological characterisation (PPI, KEGG, GO)	$\checkmark$	–	–
Synthetic positive control	$\checkmark$^*^	–	–
Real-data positive control (per-TF)	$\checkmark$	–	–

^*^The synthetic positive control evaluates the pipeline, not a specific model

The coverage pattern shows that the central observational claims (gene-level dominance, no incremental pairwise value, distributed-redundancy ablation pattern) are established in both architectures wherever testable. Forward-pass interventions (orthogonal attention replacement, MLP zeroing, intervention-fidelity diagnostics, propensity matching) are restricted to Geneformer V2-316M for the practical reasons described above; the scGPT extension in Supplementary Note 22 establishes that the distributed-redundancy pattern itself is not Geneformer-specific even though the forward-pass mechanism is.

Spearman correlation edge scores (used as a baseline alternative to attention) are computed from the same control cells used for attention extraction by ranking each gene’s expression across cells and computing pairwise rank-rank covariance. We report the absolute value of correlation as the edge score because directional sign is not directly comparable to TRRUST or perturbation labels.

### Scaling and CSSI

Scaling behaviour was analysed using archived scGPT kidney runs across three model tiers (small/medium/large), three seeds, and three cell counts (200/1,000/3,000). Controlled-composition experiments used Tabula Sapiens kidney data under three conditions: single cell type with varying *N*, mixed types with fixed composition, and fixed *N* with increasing heterogeneity.

**CSSI algorithm.** Cell-State Stratified Interpretability (CSSI) implements stratified edge scoring on heterogeneous cell populations to control the dilution effect formalised in Section “Attention-specific scaling failure and CSSI remedy”. The algorithm has four steps: (1) build a *k*-NN graph from the model’s cell embeddings ($k = 15$ default; cosine similarity in the model’s residual stream space); (2) partition cells into *K* strata via Leiden community detection on the kNN graph at resolution chosen to yield approximately *K* communities; (3) compute per-stratum edge scores (Spearman correlation for each TF–target pair within each stratum); (4) aggregate across strata using one of three reducers: *CSSI-max* (per-edge maximum across strata, used as the primary reducer because it is sensitive to cell-type-specific edges that are diluted in pooled estimates), *CSSI-mean* (per-edge arithmetic mean), or *CSSI-range* (max minus min, a measure of stratum-specific variation). The optimal *K* is data-dependent; we recommend $K = 5$–7 for moderately heterogeneous populations and report sensitivity over $K \in \{2, \ldots , 20\}$ in Additional file 1: Supplementary Note 11. The null control uses random cluster labels (shuffled within the cell population) and confirms no false-positive inflation: under uninformative strata, CSSI-max AUROC is equal to or below pooled AUROC across all *K* values tested. Full details and the formal dilution model are in Additional file 1: Supplementary Notes 1 and 11.

### Perturbation-first validation

For each perturbation *g*, we compute attention-derived or correlation-based edge scores between *g* and all other genes, then evaluate AUROC for classifying differentially expressed targets (Mann-Whitney *U* test, Benjamini-Hochberg correction, LFC threshold). We use $N_\text {ctrl} = 2{,}000$ control cells, HVG $= 2{,}000$ (K562) or 3, 309 (RPE1, with perturbation genes forced in). The primary attention layer (L13) was pre-specified based on independent DLPFC brain data (Additional file 1: Supplementary Note 11); full 18-layer profiling and nested cross-validation (Additional file 1: Fig. S5) were planned secondary analyses. Sensitivity analysis spans 27 parameter combinations varying control cell count, HVG count, and LFC threshold (Additional file 1: Supplementary Note 6).

### Confound decomposition

Gene-level features (mean expression, variance, dropout rate) and pairwise edge scores are used in logistic regression under 5-fold GroupKFold cross-validation (grouped by perturbation). We test gene-only, edge-only, and combined models. Hard-generalisation protocols use GroupKFold by target gene and joint cross-gene $\times$ cross-perturbation splits. Cross-fitted residualisation removes expression-covariate contributions from edge scores using 5-fold OLS and GBDT; residual AUROC quantifies retained TRRUST-predictive signal. Propensity-score matching uses logistic regression on gene-level features to match each DE-positive target to $k = 5$ DE-negative targets with similar covariate profiles (Additional file 1: Supplementary Note 14).

### Causal ablation

Head-masking ablation uses Geneformer’s BERT head_mask parameter (shape: $n_\text {layers} \times n_\text {heads}$; eager attention implementation) to zero out specific head outputs. We test 13 conditions: top-*k* TRRUST-ranked ($k = 5, 10, 20, 50$), composite-ranked ($k = 5, 10$), full-layer (L14), inverse (bottom-*k*), and random controls. Orthogonal interventions include uniform attention replacement (setting attention weights to 1/*n* while preserving value projections) and MLP ablation (zeroing FFN output at specific layers). Intervention fidelity is assessed by comparing hidden-state and logit cosine distances between intervened and baseline forward passes across 2,000 cells (Additional file 1: Supplementary Note 14).

### Statistical framework

All analyses apply Benjamini-Hochberg false discovery rate (FDR) correction [32] at $\alpha = 0.05$. The framework comprises 153 distinct statistical tests across 37 analyses: 95 are classified as *confirmatory* (explicit *p*-value against a directional or non-null hypothesis) and 58 as *descriptive* (effect sizes, confidence intervals, bootstrap summaries without an explicit hypothesis test). The primary correction family is the set of all 95 confirmatory tests; we additionally report two sensitivity families for robustness:

**Table 2:** Multiple-testing correction families. BH-FDR at $\alpha = 0.05$ throughout. The primary family (all 95 confirmatory tests) is the family used in the manuscript text. The two sensitivity families test whether conclusions are robust to family definition: the analysis-level family retains one primary test per analysis, and the maximal family includes all 153 tests (confirmatory + descriptive). All primary conclusions are stable across all three families (Additional file 1: Supplementary Note 16)

Family	# Tests	# Significant after BH	Sensitivity
Primary (all confirmatory)	95	63 (66%)	primary
Analysis-level (one per analysis)	27	19 (70%)	sensitivity
Maximal (confirmatory + descriptive)	153	63 (41%)	sensitivity

Effect sizes are reported as Cohen’s *d* for paired comparisons, $\rho$ for Spearman correlations, and AUROC differences ($\Delta$AUROC) for incremental-value tests. Bootstrap confidence intervals use 200 iterations with perturbation-level resampling unless otherwise noted. All numerically reported *p*-values in Results are BH-corrected as part of the primary family unless explicitly marked *(exploratory)*. Exploratory analyses (not in the confirmatory family) include: per-TF characterization beyond the per-TF AUROC bootstrap (Supplementary Note 19), the layer-by-database biological characterization profile beyond the TRRUST recovery test (Supplementary Note 17), value-weighted edge extraction (Supplementary Note 18), and the master-regulator versus signal-dependent TF comparison.

## Supplementary Information

Supplementary Material 1.

## Data Availability

All analysis scripts, data processing pipelines, source data for all figures and tables, and reproducibility instructions are deposited at Zenodo (https://doi.org/10.5281/zenodo.18701417). Complete analysis code, figure generation scripts, CSSI implementation, and computational environment specifications are available at https://github.com/Biodyn-AI/biomechinterp-framework and archived at Zenodo. All primary datasets (Tabula Sapiens, Replogle Perturb-seq, Dixit/Adamson/Shifrut datasets) are publicly available from their original sources.
