# Supplementary material for: Systematic evaluation of single-cell foundation model interpretability: attention-derived edge scores add no incremental value over gene-level features for perturbation-target prediction
Source: BMC Genomics. 2026 Jul 22;27:634. doi: 10.1186/s12864-026-12965-8 (PMC13390336; doi:10.1186/s12864-026-12965-8)
Supplement: Supplementary file 1 — Supplementary Material 1. [file 12864_2026_12965_MOESM1_ESM.pdf]

Additional file 1: Supplementary Information

Systematic evaluation of single-cell foundation model interpretability: attention-derived edge scores add no incremental value over gene-level features for perturbation-target prediction

Ihor Kendiukhov

Contents

Supplementary Table 1: Analysis and Dataset Overview **3**

1 Supplementary Note 1: Metric-Dependent Scaling Behavior **6**

2 Supplementary Note 2: Baseline Comparison **10**

3 Supplementary Note 3: Systematic Bias in Single-Component Mediation Analysis **11**

4 Supplementary Note 4: Detectability Phase Diagrams **14**

5 Supplementary Note 5: Cross-Tissue Consistency **16**

6 Supplementary Note 6: Perturbation Validation Details **17**
- 6.1 Condition-specific perturbation validation (scGPT mediation) . . . 17
- 6.2 Perturbation-first validation on Replogle CRISPRi K562 . . . 17
- 6.3 Sensitivity analysis (27 parameter combinations) . . . 17
- 6.4 Attention perturbation-first evaluation . . . 18
- 6.5 Reconciling perturbation counts . . . 18

7 Supplementary Note 7: Cross-Species Ortholog Transfer **19**

8 Supplementary Note 8: Pseudotime Directionality Audit **21**

9 Supplementary Note 9: Batch and Donor Leakage Audit **22**

10 Supplementary Note 10: Uncertainty Calibration **23**

11 Supplementary Note 11: CSSI Detailed Results **24**
- 11.1 Synthetic validation . . . 24
- 11.2 Null stress tests . . . 24
- 11.3 Real-data-structured validation . . . 25
- 11.4 Real attention matrix validation . . . 25

12 Supplementary Note 12: Synthetic Ground-Truth Validation **27**

13 Supplementary Note 13: Multi-Model Validation **28**
13.1 Geneformer V1-10M GRN recovery 28
13.2 Attention–correlation mapping 28
13.3 Residualization on expression covariates 28
13.4 Degree-preserving null models 29
13.5 TRRUST circularity sensitivity analysis 32

14 Supplementary Note 14: Mechanistic Localization Details **33**
14.1 Full 18-layer perturbation-first profile 33
14.2 Attention-specific confound decomposition 33
14.3 Original 6-condition ablation 35
14.4 Orthogonal causal interventions 35
14.5 Cross-context CRISPRa replication 36
14.6 Cross-context T-cell CRISPRi replication 37
14.7 Intervention-fidelity diagnostics 37
14.8 Propensity-matched perturbation benchmark 38
14.9 HVG protocol confound test 39

15 Supplementary Note 15: Metric-Robust Incremental-Value Analysis **41**

16 Supplementary Note 16: Statistical Test Registry and Multiple Testing Correction **42**
16.1 Framework-level statistical correction 42
16.2 Statistical test registry 42
16.3 Claim-to-evidence mapping 51
16.4 Summary statistics 52

Supplementary Methods **54**

17 Supplementary Note 17: Biological Characterization of Attention Patterns **56**

18 Supplementary Note 18: Value-Weighted Edge Extraction **58**

19 Supplementary Note 19: Per-TF Characterization (Exploratory) **59**

20 Supplementary Note 20: Limitations of Perturb-seq as a Regulatory Ground Truth **60**
20.1 Edge identification vs. effect-magnitude prediction 60
20.2 Worked example: a buffered direct edge 60
20.3 Worked example: an indirect edge that scores on Objective B 60
20.4 Susceptibility dominance 61
20.5 Implications for the paper's null result 61
20.6 What a decisive Objective-A test would require 61
20.7 Recommendation 61

21 Supplementary Note 21: Positive Control — Pipeline Sensitivity to Pairwise Signal **62**
21.1 Synthetic positive control: planted ground truth 62
21.2 Real-data positive control: per-TF AUROC against TRRUST direct targets 63
21.3 Conclusion 63

22 Supplementary Note 22: scGPT Cross-Architecture Ablation Replication **65**
22.1 Approach 65
22.2 Conditions 65
22.3 Results 66
22.4 Forward-pass head ablation via PyTorch hooks 66
22.5 Caveats and scope 68
22.6 Conclusion 68

Supplementary Table 1: Analysis and Dataset Overview

Table 1: **Master analysis overview: 37 analyses by objective, dataset, model, edge type, and key result.** Objective A = curated GRN recovery (TRRUST/DoRothEA); Objective B = perturbation-target prediction. "GF V2" = Geneformer V2-316M; "GF V1" = Geneformer V1-10M; "Corr." = Spearman correlation edges from control cells; "Attn." = attention-derived edges; "Gene" = univariate gene-level features (variance, mean, dropout). C = confirmatory (BH-corrected); D = descriptive. ✓= BH-significant under primary family; – = descriptive (no test); ns = not significant.

Analysis	Obj.	Dataset	Model	Edge / metric	C/D
**Scaling and CSSI (Notes 1, 11)**					
1. Scaling top-K F1 degradation	A	scGPT kidney scale runs	scGPT	Attn., F1 sign test ✓	C
2. Continuous AUROC reverse	A	scGPT kidney scale runs	scGPT	Attn., AUROC monotone	D
3. Controlled composition	A	TS kidney	–	Corr., heterogeneity ρ ✓	C
4. CSSI synthetic mitigation	A	Synthetic GRN	–	CSSI-max F1 ✓	C
5. CSSI null inflation	A	PBMC + null	GF V2	CSSI-max vs. shuffle	D
6. CSSI biological data (15 cell types)	A	TS immune	–	CSSI-max Wilcoxon ✓	C
7. CSSI on real attention (per-layer)	A	DLPFC brain	GF V2	CSSI per-layer AUROC	D
**Multi-model / cross-architecture (Notes 13, 22)**					
8. GF V1 GRN recovery	A	DLPFC brain	GF V1	Attn., AUROC near chance (ns)	C
9. scGPT GRN recovery	A	DLPFC + tissue	scGPT	Attn., AUROC near chance (ns)	C
10. Attention vs. co-expression mapping	A	TS multi-tissue	GF, scGPT	Spearman ✓	C
11. scGPT cross-arch ablation (Note 22)	A	TS immune/kid./brain	scGPT	Edge-level top-50 ablation $\Delta \leq 0.003$	D
**Perturbation-first validation (Notes 6, 14)**					
12. Replogle K562 corr. AUROC	B	Replogle K562 CRISPRi	–	Corr., AUROC = 0.696 ✓	C
13. Sensitivity (27 conditions)	B	Replogle K562	–	Corr., AUROC 0.62–0.76 ✓	C
14. GF V2 attn. L13 vs. corr.	B	Replogle K562	GF V2	Wilcoxon $p = 0.73$ (null)	C
15. Full 18-layer attn. profile	B	Replogle K562	GF V2	AUROC 0.47–0.74 (per-layer)	D
16. Nested-CV best layer (L15)	B	Replogle K562	GF V2	$\Delta = +0.040$ ✓	C
**Trivial baselines and incremental value (Note 15)**					
17. K562 trivial baselines	B	Replogle K562	–	Variance AUROC 0.881 ✓	C
18. K562 incremental value	B	Replogle K562	GF V2	ΔAUROC $\leq$ 0.002 (null)	C
19. Hard generalisation (3 splits)	B	Replogle K562	GF V2	ΔAUROC $\leq$ 0.005 (null)	C

Continued on next page

Table continued

Analysis	Obj.	Dataset	Model	Edge / metric	C/D
20. K562 expression residualisation	A	TS immune (DLPFC)	GF V2	Attn loses 76% ✓	C
21. Degree-preserving null	A	TS immune	GF V2	$z = 3.63$ ✓	C
22. Propensity-matched K562	B	Replogle K562	GF V2	ΔAUROC ≈ 0 (null)	C
23. Per-TF bootstrap	A	TS immune	GF V2	7/18 above chance ✓	C
**Causal ablation (Note 14)**					
24. Head ablation (top-K TRRUST-ranked)	B	Replogle K562	GF V2	13 conditions, all null	C
25. Random head ablation control	B	Replogle K562	GF V2	Random-20 $d = 0.33$ ✓	C
26. Uniform attention replacement	B	Replogle K562	GF V2	$\Delta = 0$ (null)	C
27. MLP zero ablation	B	Replogle K562	GF V2	$\Delta = 0$ (null)	C
28. Intervention-fidelity diagnostics	B	Replogle K562	GF V2	Hidden cos. 0.02–0.19	D
**Cross-context replication (Note 14)**					
29. K562 CRISPRa (Adamson)	B	Adamson K562 CRISPRa	GF V2	Attn < Corr ($p < 10^{-6}$) ✓	C
30. RPE1 CRISPRi (Replogle)	B	Replogle RPE1	GF V2	Attn > Corr ($d = 0.47$) ✓	C
31. RPE1 confound battery	B	Replogle RPE1	GF V2	Gene-only AUROC 0.942 (null Δ)	C
32. Shifrut T-cell CRISPRi	B	Shifrut T cells	GF V2	Attn vs Corr $p = 0.81$ (ns)	C
33. iPSC neurons (Tian)	B	Tian neurons	GF V2	$d = 0.80$ ($p = 0.078$, ns)	C
**Biological characterisation and positive controls (Notes 17, 21)**					
34. Layer-by-database profile	A	TS immune K562	GF V2	6/6 trends BH-significant ✓	C
35. Synthetic positive control (3 var.)	B	Synthetic Perturb-seq	–	$\Delta = +0.45$–$+0.52$ planted	D
36. Real-data positive control (per-TF)	A	TS immune attention	GF V2	9/9 TFs above chance @ L17	D
**Boundary-condition tier (Notes 7–10)**					
37. Cross-species ortholog ($\rho = 0.74$)	A	TS lung + mouse lung	–	Spearman ✓	C

Table 2: **Perturbation sample counts across datasets and configurations.**

Dataset	Modality / Config	DE Thresh.	n	Notes
Replogle K562	CRISPRi (baseline)	LFC>0.1	44	Mann-Whitney, HVG=1000, N_{ctrl}=500
Replogle K562	CRISPRi (primary)	LFC>0.5	151	Welch t, HVG=2000, N_{ctrl}=2000
Replogle K562	CRISPRi (attention)	LFC>0.5	280	Welch t, HVG=2000; more genes evaluable with attention tokenization
Adamson K562	CRISPRa	LFC>0.5	77	K562, activation modality
Shifrut T cells	CRISPRi	LFC>0.1	7	Primary human T cells; weak DE effects require lenient threshold
Replogle RPE1	CRISPRi	LFC>0.5	1,251	hTERT-RPE1; perturbation genes forced into HVG
Tian iPSC neurons	CRISPRi	LFC>0.1	7	iPSC-derived glutamatergic neurons

1 Supplementary Note 1: Metric-Dependent Scaling Behavior

To test whether increasing dataset size improves interpretability, we analyzed archived scGPT kidney scaling runs across three model tiers (small/medium/large), three seeds per tier, and three cell counts (200, 1,000, 3,000). Each run yields an attention-derived score for each directed gene pair; we construct a sparse GRN by retaining the top-100 targets per source gene and evaluate recovery against TRRUST and DoRothEA reference edges restricted to the run-specific gene universe.

Evidence: scaling degrades recovery. TRRUST F1 decreases with cell count across model tiers (Supplementary Fig. 1); the 200→1,000 change is negative in all 9 tier×seed pairs (exact one-sided sign test $p = 0.00195$). The same pattern holds for DoRothEA ($p = 0.00195$). The 1,000→3,000 step shows continued degradation in 7/9 pairs (sign test $p = 0.09$), weaker but directionally consistent.

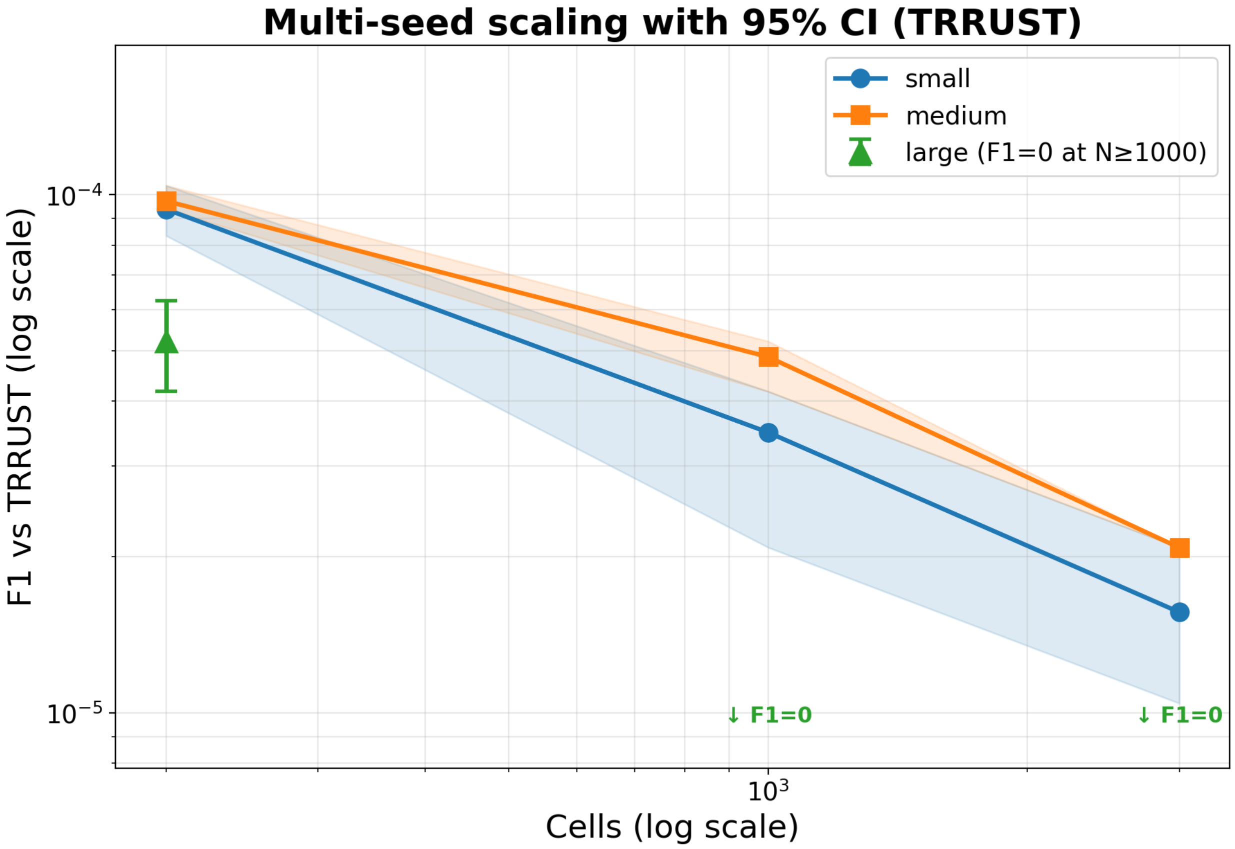

Figure 1: **Metric-dependent scaling behavior in scGPT attention-derived GRN recovery (kidney).** TRRUST F1 with 95% confidence intervals across three model tiers (small/medium/large) and cell counts (200/1,000/3,000).

Retrieval collapse. The number of recovered true positives decreases toward (and sometimes below) random expectation as N increases (Supplementary Fig. 2), indicating that scaling can reduce enrichment rather than merely saturate.

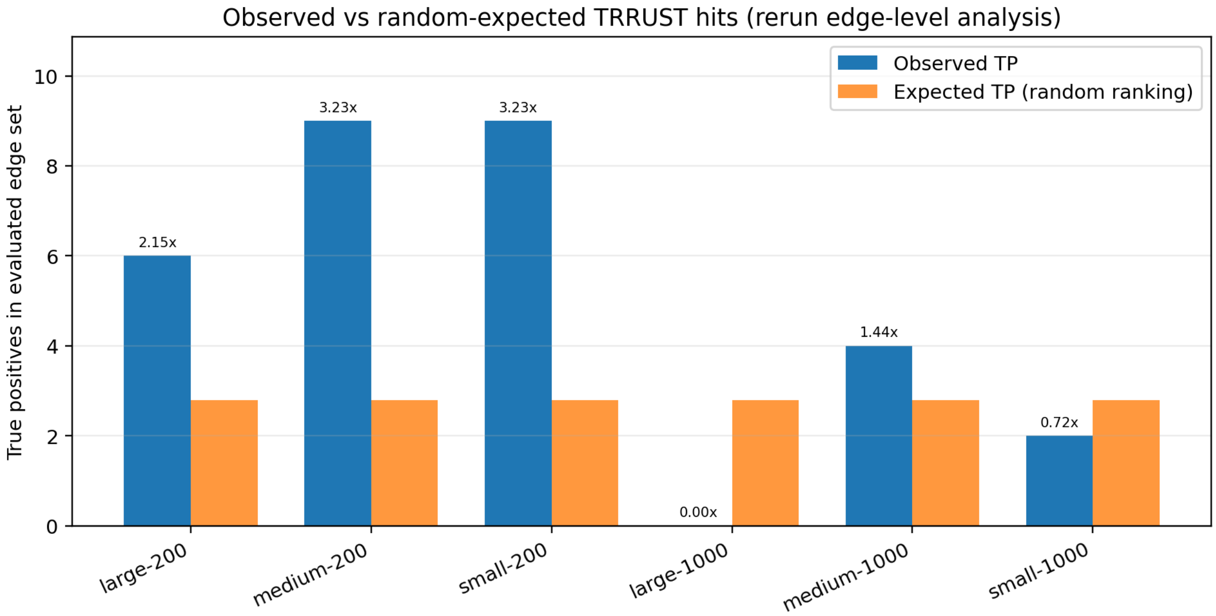

Figure 2: **Retrieval collapse with scaling (scGPT kidney).** Observed true positives versus random-expected true positives for attention-derived GRNs.

Robustness also degrades with scaling. Between-seed stability decreases substantially from 200 to 1,000 cells (Supplementary Table 3): edge-set Jaccard overlaps drop by 46.6–47.9% depending on tier (one-sided Mann–Whitney $p = 2.1 \times 10^{-4}$ across all tiers/seeds).

Table 3: **Between-seed robustness of inferred edge sets (scGPT kidney).**

Tier	Jaccard (200)	Jaccard (1,000)	Spearman (200)	Spearman (1,000)
Small	0.572	0.305	0.211	-0.065
Medium	0.562	0.293	0.183	-0.110
Large	0.536	0.285	0.083	-0.130

Heterogeneity proxy (cell-type richness). Reconstructing the exact subsampling used during attention extraction (manifest-specified random seeds), the number of observed kidney cell types increases with N and is strongly anti-correlated with TRRUST F1 across runs (Spearman $\rho = -0.76$, $p = 4.3 \times 10^{-5}$; Supplementary Fig. 3).

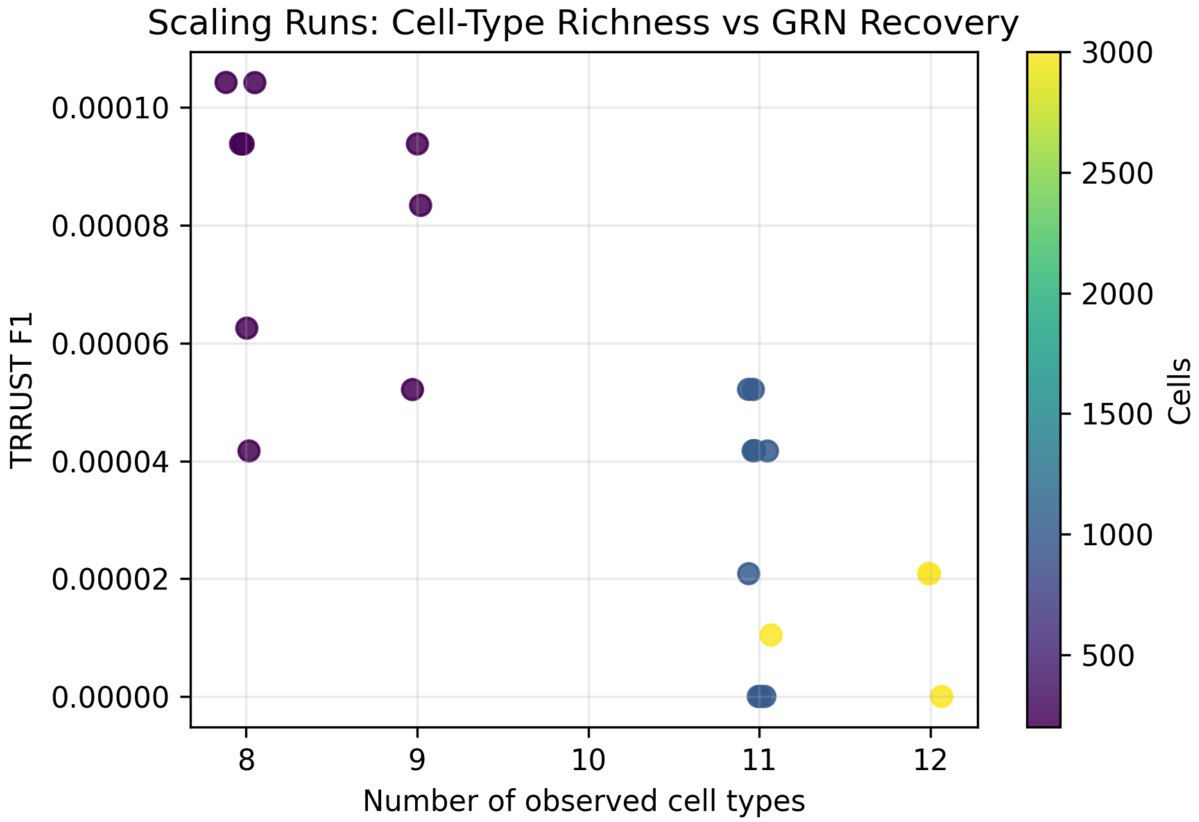

Figure 3: **Scaling runs: composition diversity proxy.** Across the scGPT kidney scaling grid, the number of observed cell types in the sampled cells increases with N and is anti-correlated with GRN recovery.

K-sensitivity and continuous AUROC. The top-K F1 metric used above is inherently sensitive to K, and the 1,930-gene universe of these scaling runs contains only 51 TRRUST edges among $\sim$3.7 million candidate pairs (positive rate $< 0.002\%$). To test whether the scaling finding is metric-dependent, we re-evaluated the archived attention-score matrices at $K \in \{20, 50, 100, 200, 500\}$ and computed continuous-score AUROC (no top-K thresholding). F1 values are effectively zero at all K values ($\sim 10^{-4}$), confirming that near-zero absolute performance is driven by extreme reference sparsity rather than a specific K choice. However, continuous-score AUROC tells a different story: it *improves* monotonically with cell count (mean 0.858 at $N = 200$, 0.925 at $N = 1{,}000$, 0.934 at $N = 3{,}000$; 0/9 runs show degradation; Supplementary Fig. 4). This reversal under continuous AUROC indicates that the scaling behavior is metric-dependent: while the thresholded top-K edge set degrades with N, the continuous *ranking* of all gene pairs against curated references improves.

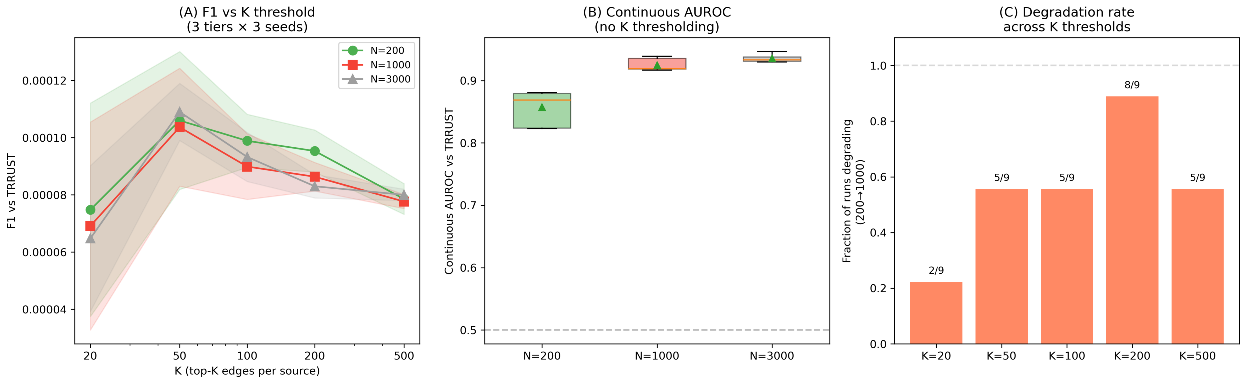

Figure 4: **K-sensitivity and continuous AUROC for scaling runs.** (A) F1 vs K at different cell counts. (B) Continuous AUROC improves monotonically with cell count ($0.858 \to 0.925 \to 0.934$). (C) Fraction of runs showing degradation across K values.

Controlled-composition scaling. To test whether top-K scaling degradation is driven by sample size N or by heterogeneity, we conducted a controlled experiment on Tabula Sapiens kidney data using correlation-based edge scores under three conditions (Supplementary Fig. 5): (i) a single cell type (kidney epithelial) with varying N (100–3,000), (ii) mixed cell types with fixed equal composition across N (100–1,000), and (iii) fixed $N = 500$ with increasing heterogeneity (1–7 cell types). Under condition (i), AUROC shows a weak non-significant downward trend (Spearman $\rho = -0.33$, $p = 0.079$). Under condition (ii), AUROC is stable ($\rho = -0.05$, $p = 0.82$). Under condition (iii), AUROC actually *increases* with heterogeneity ($\rho = +0.63$, $p = 10^{-4}$).

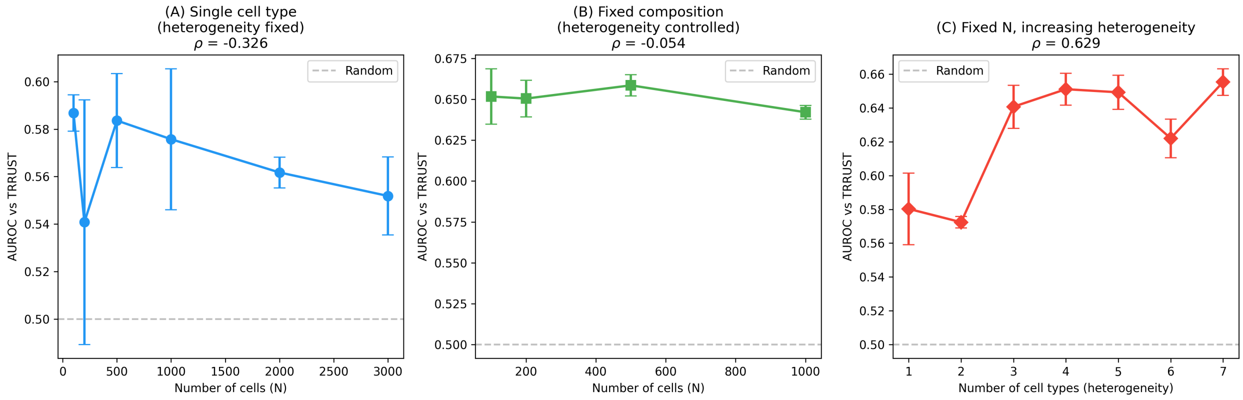

Figure 5: **Controlled-composition scaling.** (A) Single cell type: no significant degradation with N. (B) Fixed composition: stable AUROC. (C) Increasing heterogeneity at fixed N: AUROC increases.

2 Supplementary Note 2: Baseline Comparison

We evaluated multiple baseline approaches on DLPFC brain tissue data (500 randomly sampled cells, top 500 most variable genes): Spearman correlation, mutual information, GENIE3 [1], GRNBoost2 [2], and attention-based edge scores. All methods were evaluated against TRRUST and DoRothEA using AUROC, AUPRC, and Precision@10k.

All approaches show similar poor performance, with AUROC values clustering around 0.50–0.53: Spearman correlation (AUROC 0.521), mutual information (0.518), GENIE3 (0.523), GRNBoost2 (0.526), and attention-based methods (0.524). State-of-the-art dedicated GRN inference algorithms achieve nearly identical performance to attention-based approaches, while requiring 89–127 seconds computation time versus 0.1 seconds for attention extraction. The convergence toward AUROC ≈ 0.5 in this DLPFC brain setting suggests that benchmarking against curated TF–target databases in context-mismatched tissues can be dominated by evaluation limitations.

3 Supplementary Note 3: Systematic Bias in Single-Component Mediation Analysis

Activation patching has become the standard tool for localizing mechanistic function in transformers [3–5]. However, the standard single-component protocol implicitly assumes additivity. We formalize the bias problem following the causal mediation framework of Pearl [6] and Imai et al. [7]. For mediator component i, the bias relative to the interaction-aware Shapley value ϕ_i [8, 9] decomposes as:

$$b_i = \hat{m}_i - \phi_i = -\sum_{|S|\geq 2,\, i\in S} \frac{\mu(S)}{|S|} + \varepsilon_i \tag{1}$$

where $\mu(S)$ represents Möbius interaction coefficients. We introduce an observable lower bound on aggregate non-additivity:

$$A_{\text{lb}} = \max\big(0,\ |R| - 1.96 \cdot \text{SE}(R)\big) \tag{2}$$

where $R = TE - \sum_i \hat{m}_i$ is the residual between total effect and the sum of single-component estimates.

Analysis of frozen cross-tissue mediation archives revealed substantial and frequent additivity violations. Across 16 run-pairs, lower bounds on aggregate non-additivity were positive in 10 cases (rate 0.625), with median $A_{\text{lb}}/|TE| = 0.725$ (Supplementary Fig. 6). Ranking certificates proved fragile: mean certified pair coverage dropped from 0.0669 at $\lambda = 1$ to 0.0032 by $\lambda \geq 3$ (Supplementary Fig. 7).

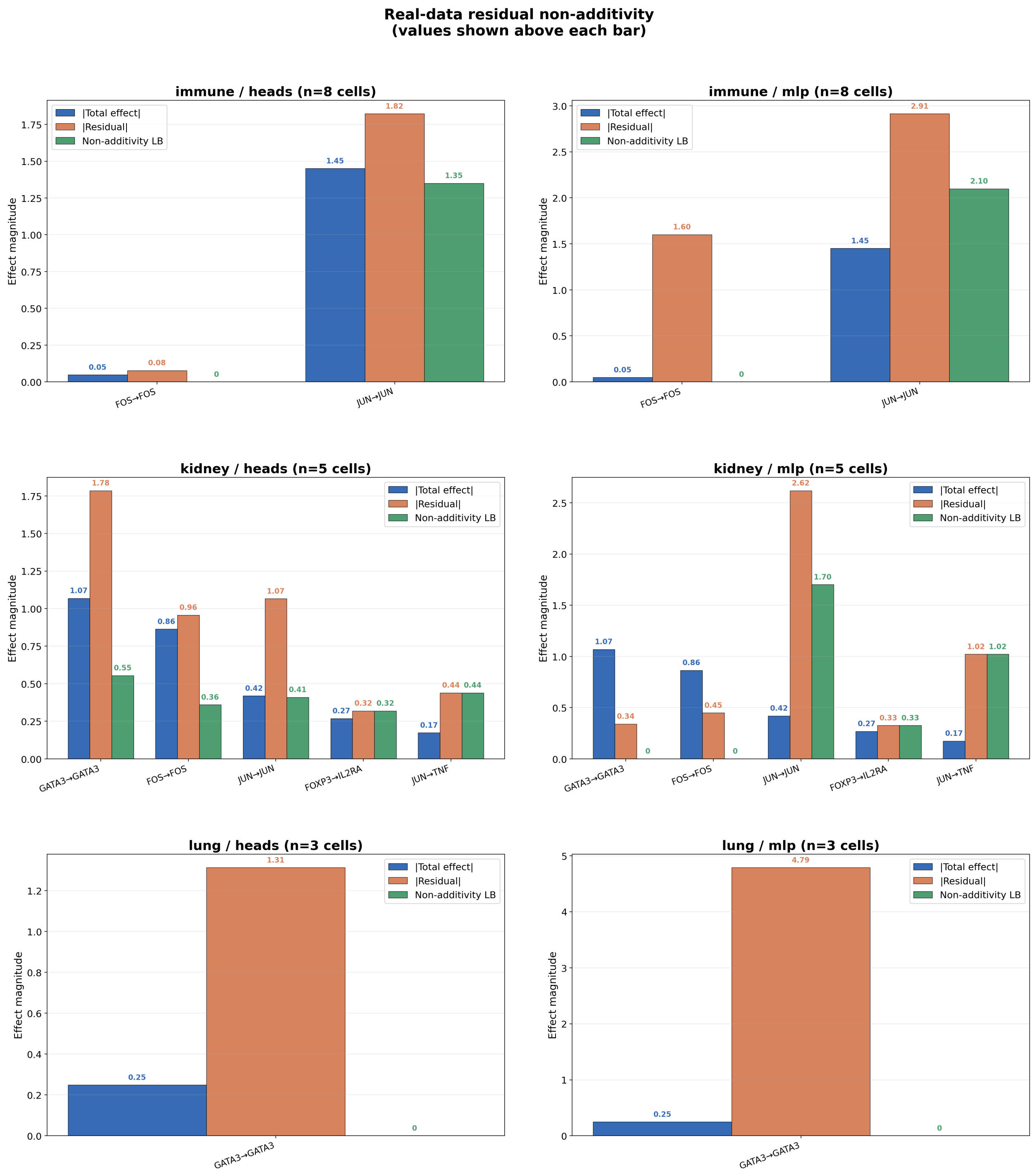

Figure 6: **Non-additivity in mediation analysis.** Absolute total effect, residual non-additivity, and lower-bound interaction magnitude per run-pair.

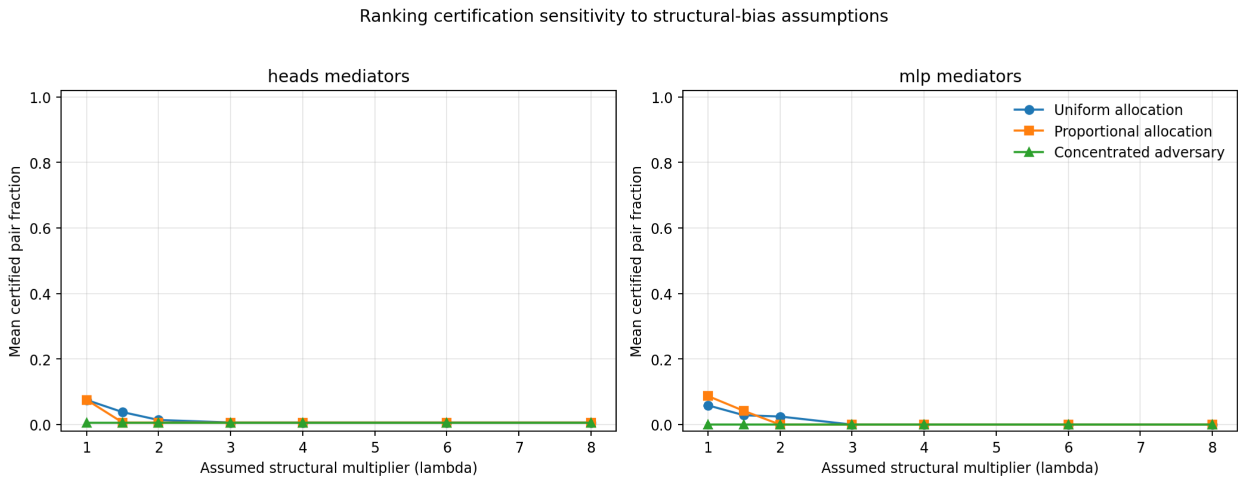

Figure 7: **Ranking certificate fragility.** Mean certified pair fraction versus structural multiplier λ.

Non-additivity is common in our data (62.5% of 16 run-pairs across 3 tissues) and concentrates in biologically meaningful contexts. These findings indicate that standard single-component rankings may be unreliable in contexts with complex regulatory interactions, though the sample warrants further validation at larger scale. Mechanistic claims should be accompanied by the residual non-additivity ratio $A_{\text{lb}}/|TE|$, ranking certificates, and interaction-aware alternatives such as Shapley-value decomposition.

4 Supplementary Note 4: Detectability Phase Diagrams

We developed a closed-form detectability framework rooted in statistical detection theory [10]. For a mechanistic signal with effect size $|\mu|$, noise scale σ, and tail inflation factor τ, the required sample size for detection is:

$$n^* = \left(\frac{(z_{1-\alpha/(2m)} + z_{\text{power}})\, \tau\, \sigma}{|\mu|} \right)^2 \tag{3}$$

Under sub-Gaussian baseline conditions, intervention-like signals required only 44.4% as many cells as attention-like signals for equivalent detectability (Supplementary Fig. 8). However, this advantage collapsed progressively under tail inflation, with the relative cell ratio approaching unity when $\tau > 3$.

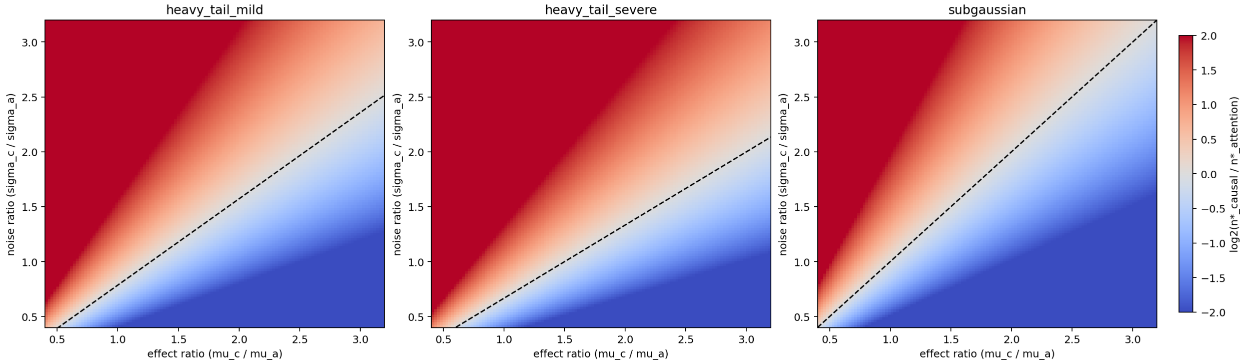

Figure 8: **Detectability phase diagrams.** Different regimes where attention-like versus intervention-like signals become detectable.

Robust estimation (median-based or Huber M-estimators [11]) expanded the feasible detection region by 37% under 10% contamination. Real-data calibration showed projected relative cell ratios below one in most bootstrap draws, but confidence intervals remained wide (Supplementary Fig. 9).

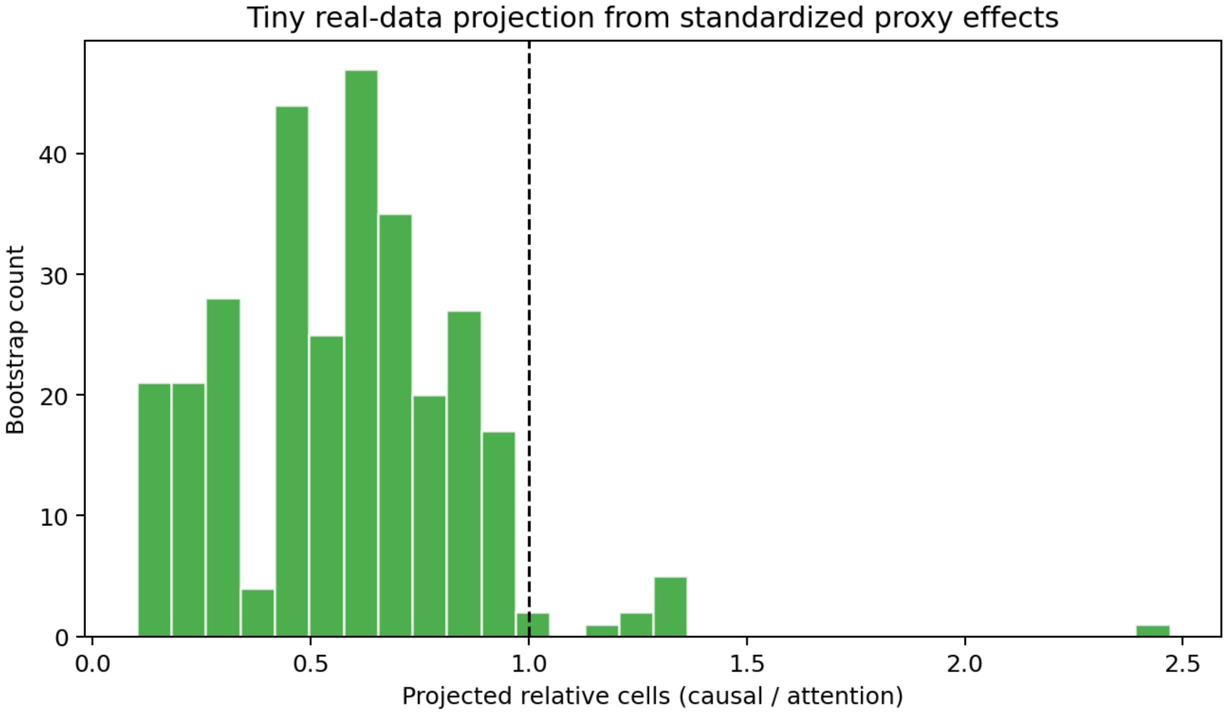

Figure 9: **Real data detectability calibration.** Bootstrap distribution of projected relative cell requirements.

5 Supplementary Note 5: Cross-Tissue Consistency

Cross-tissue analysis across immune, kidney, and lung tissues revealed Spearman correlations ranging from -0.44 to 0.71, with only two of six pair-granularity comparisons surviving FDR control at $\alpha = 0.05$ (Supplementary Fig. 10). Limited transferability is consistent with known tissue-specificity of gene regulation. Negative correlations in some tissue pairs suggest either genuine context-dependent regulation or tissue-specific confounds.

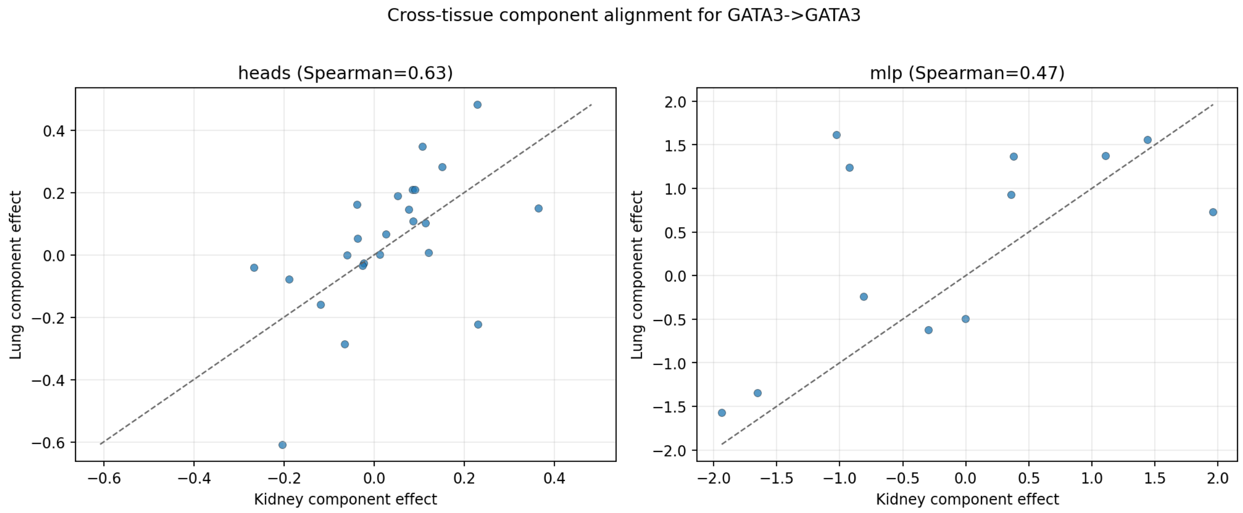

Figure 10: **Cross-tissue consistency variability.** Scatter plots of component-level effects between tissue pairs.

An important alternative interpretation is that technical batch effects between tissue datasets may partly explain the low consistency. Our batch leakage analysis (Supplementary Note 9) confirms that technical covariates are recoverable from edge features. Distinguishing genuine regulatory rewiring from technical artifacts would require matched protocols across tissues.

6 Supplementary Note 6: Perturbation Validation Details

6.1 Condition-specific perturbation validation (scGPT mediation)

Counterfactual validation against four CRISPR Perturb-seq datasets revealed weak and condition-specific alignment. The strongest positive signal appears in Dixit 13-day: consistency is positive ($\rho = 0.269$, $p = 0.032$) and remains positive after confound adjustment ($\rho = 0.199$, $p = 0.020$). Dixit 7-day shows weaker non-significant consistency ($\rho = 0.112$, $p = 0.15$). Adamson shows marginal agreement ($p = 0.089$). Shifrut shows raw anti-alignment ($\rho = -0.325$, $p = 0.031$) that collapses after adjustment ($\rho = 0.004$, $p = 0.876$). Under framework-level BH correction, only the Dixit 13-day confound-adjusted correlation survives (adj. $p = 0.042$).

6.2 Perturbation-first validation on Replogle CRISPRi K562

Under our primary parameterization ($N_{\text{ctrl}} = 2000$, HVG $= 2000$, LFC > 0.5, Welch's t-test with BH-FDR correction), the mean per-gene AUROC was 0.696 ($n = 151$ evaluable perturbations, $p < 10^{-4}$; Supplementary Fig. 11).

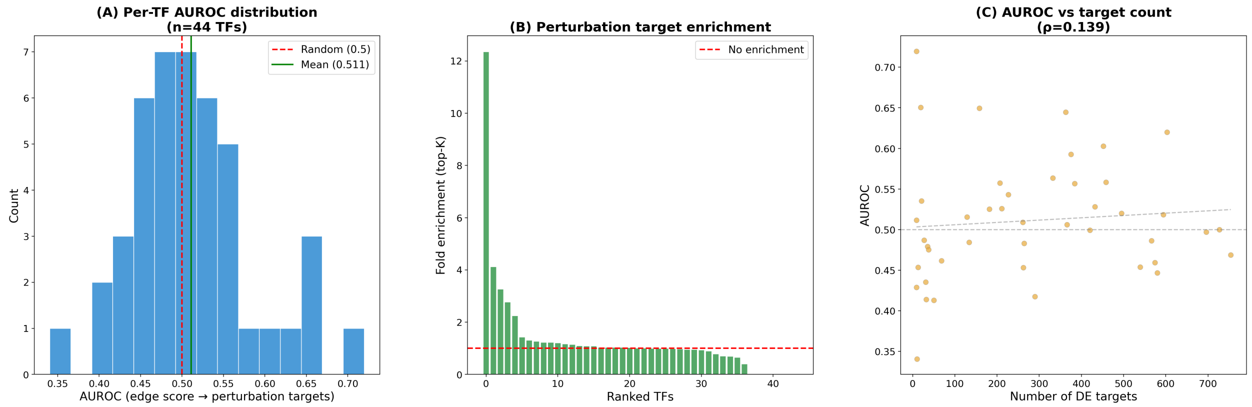

Figure 11: **Perturbation-first validation (Replogle CRISPRi K562).** Per-gene AUROC for predicting DE targets from correlation-based edge scores under primary parameterization.

6.3 Sensitivity analysis (27 parameter combinations)

We systematically varied the number of control cells ($N_{\text{ctrl}} \in \{500, 2000, 10000\}$), gene universe (HVG $\in \{1000, 2000, 5000\}$), and DE stringency (LFC threshold $\in \{0.25, 0.5, 1.0\}$), yielding 27 parameter combinations (Supplementary Fig. 12). All 27 conditions yielded AUROC significantly above chance ($p < 0.005$). AUROC ranged from 0.619 to 0.756.

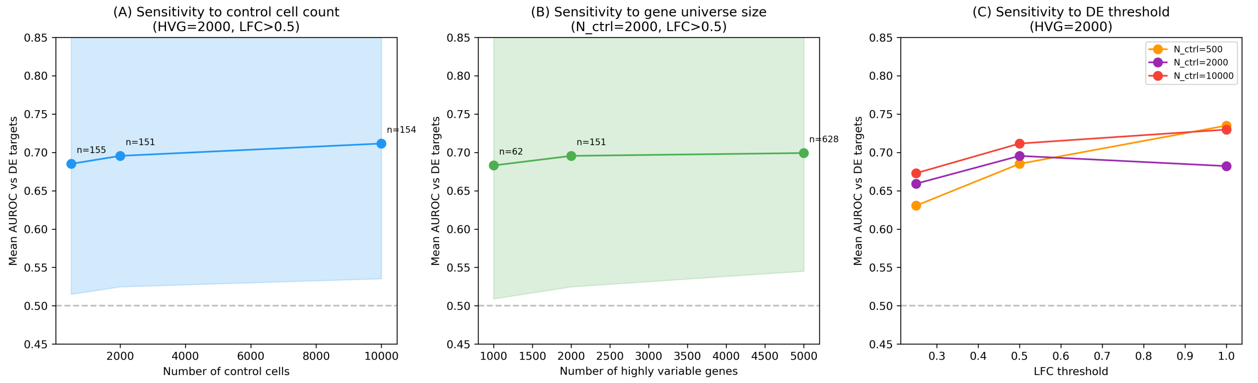

Figure 12: **Perturbation-first sensitivity analysis (27 parameter combinations).** Mean AUROC varies with (A) number of control cells, (B) gene universe size, and (C) LFC threshold.

6.4 Attention perturbation-first evaluation

Geneformer V2-316M attention-derived AUROC is statistically indistinguishable from correlation at all three layers: L6 $= 0.705 \pm 0.145$ ($p = 0.76$), L13 $= 0.704 \pm 0.147$ ($p = 0.73$), L18 $= 0.708 \pm 0.157$ ($p = 0.75$), vs. correlation $= 0.703 \pm 0.164$ (Supplementary Fig. 13).

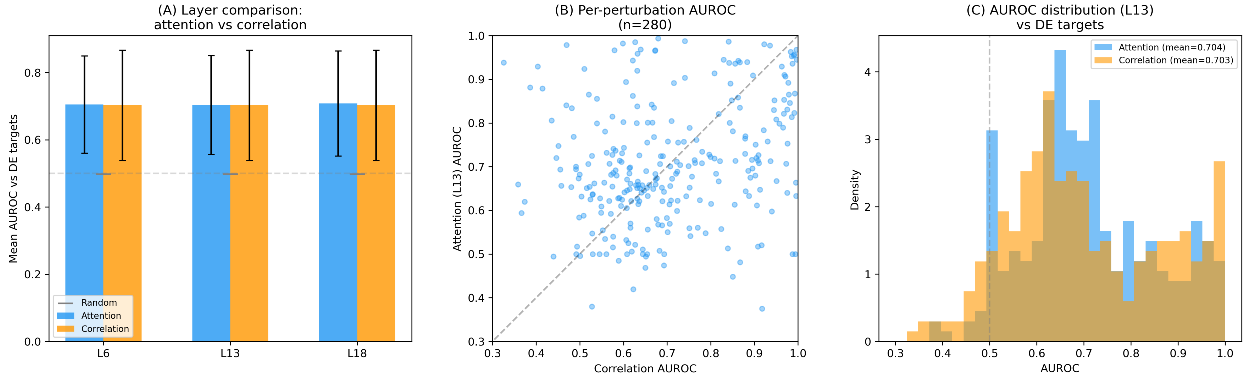

Figure 13: **Attention-derived edges are indistinguishable from correlation on perturbation-first prediction.** Geneformer V2-316M attention (L13) vs. correlation-based edges ($n = 280$).

6.5 Reconciling perturbation counts

The number of evaluable perturbations varies across parameterizations because each imposes different inclusion criteria. The lenient baseline (Mann–Whitney, $|\text{LFC}| > 0.1$, 500 control cells) yields 44 evaluable genes; the primary correlation-based parameterization (Welch's t-test, LFC > 0.5, HVG $= 2{,}000$, $N_{\text{ctrl}} = 2{,}000$) yields $n = 151$; and the attention comparison under the same DE thresholds yields $n = 280$ because the expanded gene-matching procedure identifies more evaluable perturbations when matching against the full tokenized gene set.

7 Supplementary Note 7: Cross-Species Ortholog Transfer

To test whether mechanistic signals generalize across species, we performed a systematic stress test of TF–target edge transfer between human and mouse lung using correlation-based edge scores computed independently in each species [12]. Cross-species comparison of 25,876 matched TF–target edges revealed strong global conservation (Supplementary Fig. 14). The Spearman rank correlation between human and mouse edge scores was $\rho = 0.743$ ($p < 10^{-300}$). Sign agreement was 88.6% across all shared edges, rising to 100% for edges with $|\rho| > 0.4$ in both species. Top-k overlap was enriched 8- to 484-fold over random expectation (Supplementary Table 4).

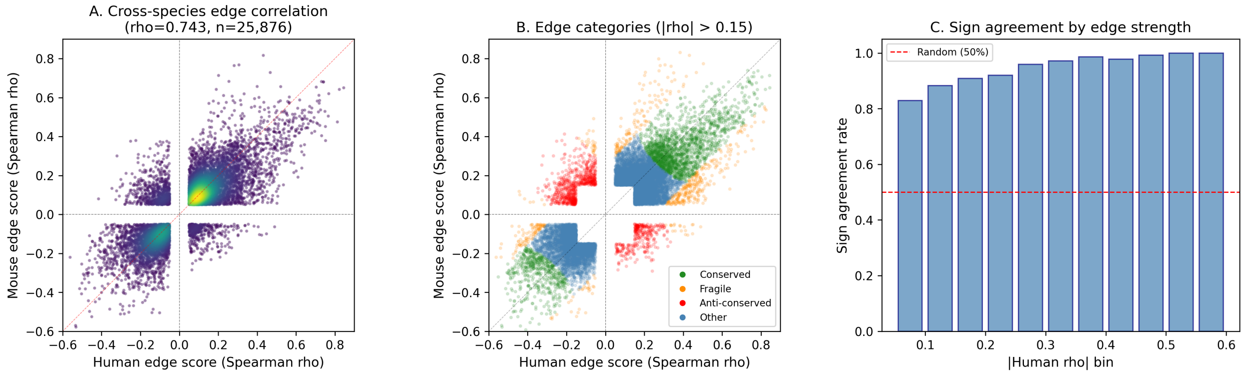

Figure 14: **Cross-species edge score conservation.** Scatter plot of Spearman ρ for 25,876 matched TF–target edges between human and mouse lung.

Table 4: **Top-k overlap between human and mouse edge rankings.**

Top k	Observed	Expected	Fold
100	26	0.1	484×
500	153	1.3	114×
1,000	289	5.4	54×
5,000	1,094	134.2	8.2×

However, per-TF conservation was highly non-uniform (Supplementary Fig. 15). Lineage-specifying factors showed near-perfect transfer: XBP1 ($\rho = 0.90$), EPAS1 (0.89), ERG (0.88), NKX2-1 (0.81). In contrast, signaling-responsive TFs showed poor conservation: CTNNB1 (0.01), HIF1A (0.10), STAT1 (0.06), CEBPB (0.13). Fragile edges (599 total) were enriched for immune-cell-specific RUNX3 targets with species-divergent expression.

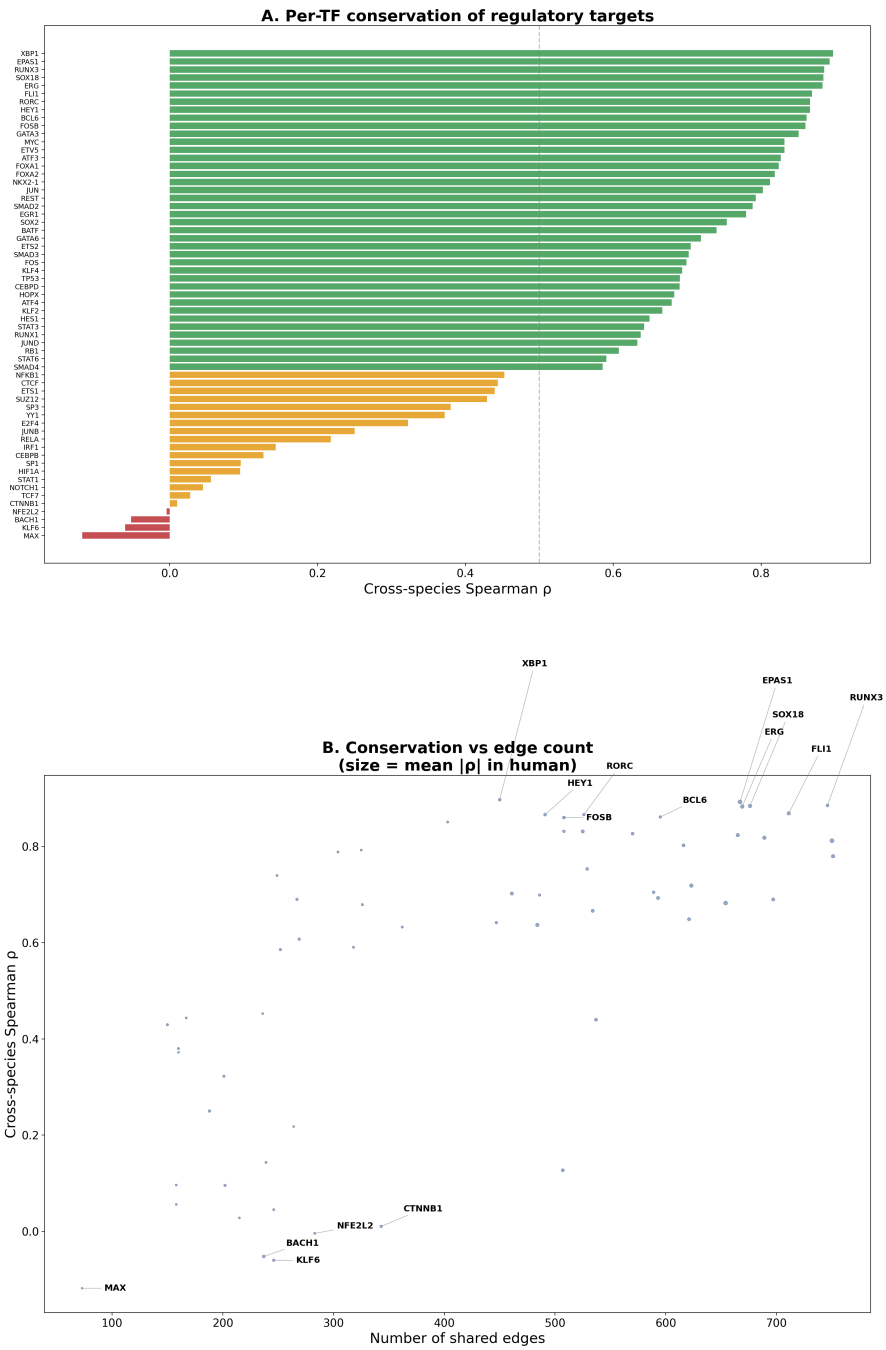

Figure 15: **Per-TF conservation of regulatory targets across species.** (A) Horizontal bar chart of cross-species Spearman ρ for each TF's target set (green: $\rho \geq 0.5$, orange: $0 \leq \rho < 0.5$, red: $\rho < 0$). Lineage-specifying TFs (XBP1, EPAS1, RUNX3) show near-perfect conservation ($\rho > 0.88$), while signaling-responsive TFs (MAX, BACH1, CTNNB1) show poor or negative conservation. (B) Conservation versus number of shared orthologous edges. Point size reflects mean absolute attention weight ($|\rho|$) in human. High-conservation TFs tend to have more shared edges, but low-conservation outliers (MAX, KLF6) persist despite moderate edge counts.

Ortholog-based edge transfer should be stratified by TF class: lineage-specifying programs can be transferred with high confidence, while signaling-responsive and composition-dependent edges require species-specific validation.

8 Supplementary Note 8: Pseudotime Directionality Audit

Using diffusion pseudotime [13] in three Tabula Sapiens immune lineages, we tested 56 curated TF–target pairs for lag-based directional consistency. Only 12 of 56 TF–target pairs (21.4%) were directionally consistent (Supplementary Fig. 16). Consistency varied by lineage: myeloid pairs showed the highest rate (6/17, 35.3%), followed by T cell (4/24, 16.7%) and B cell (2/15, 13.3%). The mean directionality score marginally exceeded a shuffled-pseudotime null ($p = 0.068$) but not a random gene-pair null ($p = 0.37$; Supplementary Fig. 17); after framework-level FDR correction, this effect is not significant ($q = 0.124$).

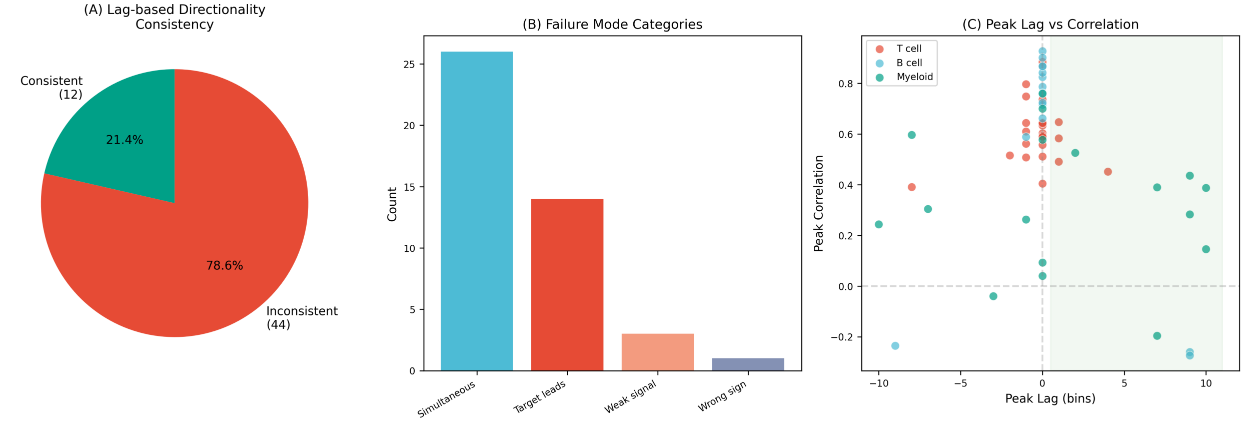

Figure 16: **Pseudotime directionality failures.** Only 12/56 TF–target pairs (21.4%) show directionally consistent lag-based ordering.

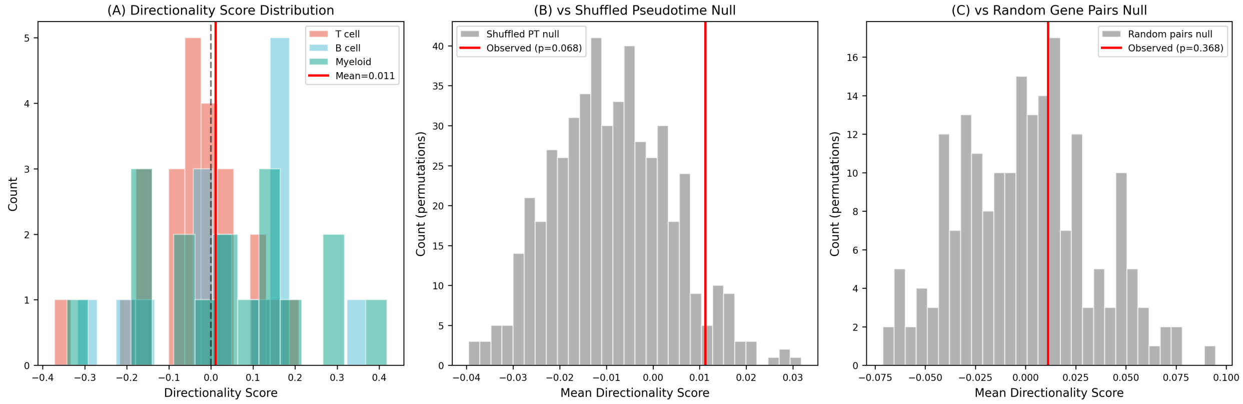

Figure 17: **Pseudotime null comparisons.** Mean directionality score marginally exceeds shuffled-pseudotime null ($p = 0.068$) but not random gene-pair null ($p = 0.37$).

Pseudotime should be treated as a qualitative sanity check rather than a pass/fail validator for mechanistic edges. Perturbation-based validation and time-resolved modalities (e.g., RNA velocity) provide more direct temporal or causal evidence.

9 Supplementary Note 9: Batch and Donor Leakage Audit

We conducted a systematic leakage audit across three Tabula Sapiens tissue compartments. Leakage classifiers revealed substantial technical signal in edge-product features (Supplementary Fig. 18). Donor identity was recoverable well above chance: immune dataset AUC 0.85–0.87 (21 donors); lung dataset AUC 0.94–0.96 (4 donors). Assay method (10X vs. Smart-seq2) was the dominant confound, recoverable at AUC 0.96–0.99.

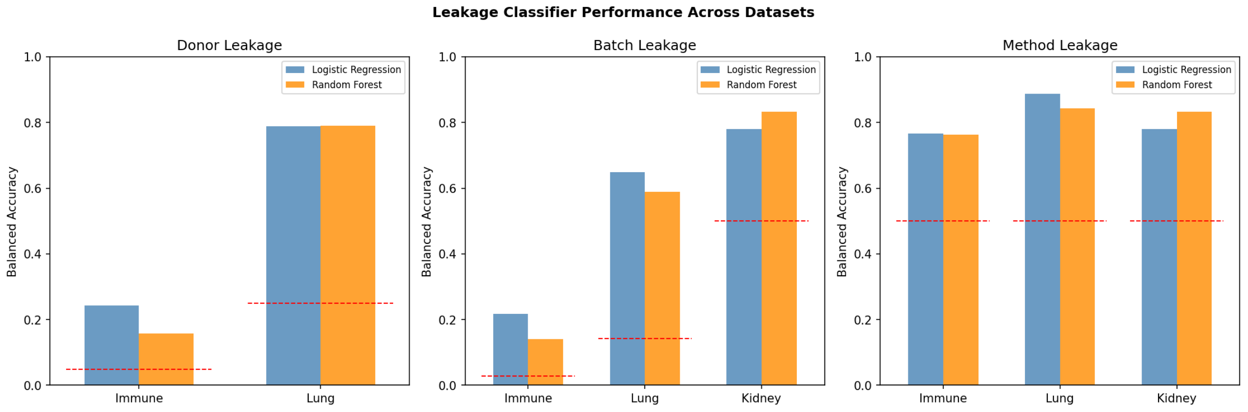

Figure 18: **Cross-dataset leakage summary.** Balanced accuracy and AUC for donor, batch, and method classification from edge-product features.

The practical impact was dataset-dependent. The well-balanced lung dataset showed remarkably stable aggregate edge scores under donor-balanced resampling ($r = 0.997$, 10.1% blacklisted). The imbalanced immune dataset showed genuine instability ($r = 0.929$, 54.6% blacklisted, 17.1% sign-flipped; Supplementary Fig. 19).

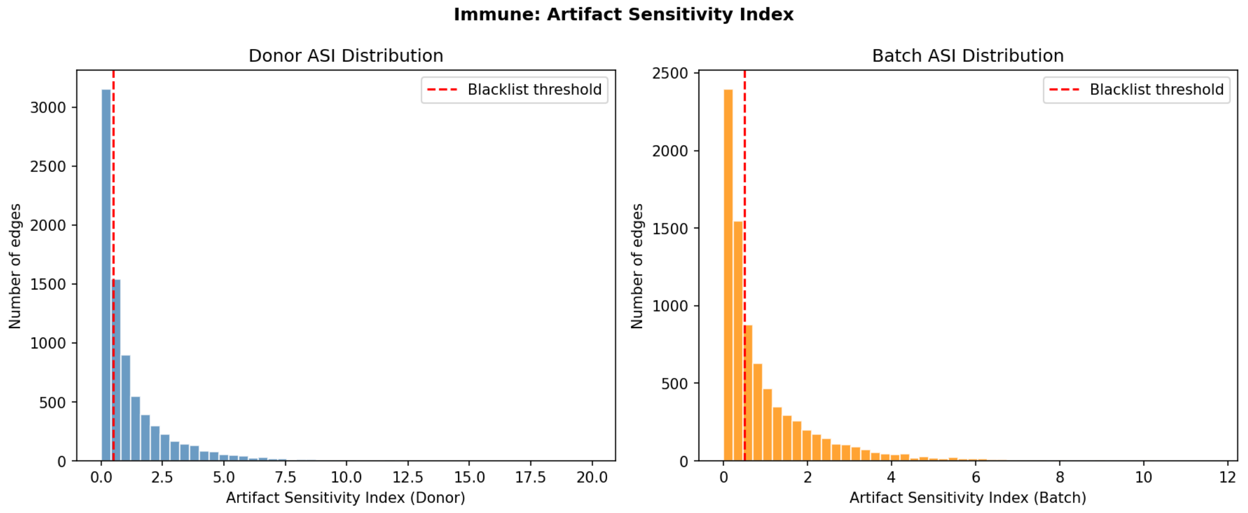

Figure 19: **Artifact Sensitivity Index distribution.** Immune tissue has 54.6% of edges exceeding ASI > 0.5.

Edge score evaluation must use donor-stratified splits, never random CV when donor metadata is available. The generalization gap (6.6 percentage points in lung) should be reported as a built-in quality check.

10 Supplementary Note 10: Uncertainty Calibration

All six edge-scoring methods produced severely miscalibrated scores against Perturb-seq ground truth: raw Expected Calibration Error (ECE) ranged from 0.269 (ensemble) to 0.469 (LASSO; Supplementary Fig. 20). Post-hoc calibration dramatically improved score quality: isotonic regression reduced ECE to 0.062–0.079 (4–7× reduction) without changing discrimination.

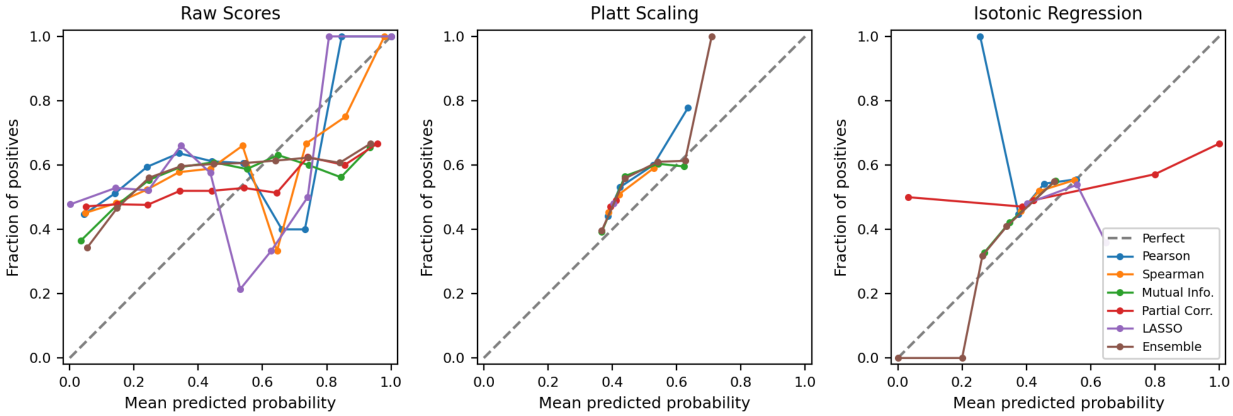

Figure 20: **Edge score calibration.** Reliability diagrams showing fraction of true positives vs. mean predicted probability. Platt scaling and isotonic regression reduce ECE by 4–7×.

Split conformal prediction sets achieved valid marginal coverage (≥95%) for mutual information and ensemble methods at $\alpha = 0.05$, with 13.4% singleton prediction sets for mutual information (Supplementary Fig. 21). Critically, calibrators did not transfer across datasets: K562-trained calibrators applied to the Shifrut T cell dataset yielded ECE 0.320–0.424, compared to 0.002–0.031 for locally trained calibrators.

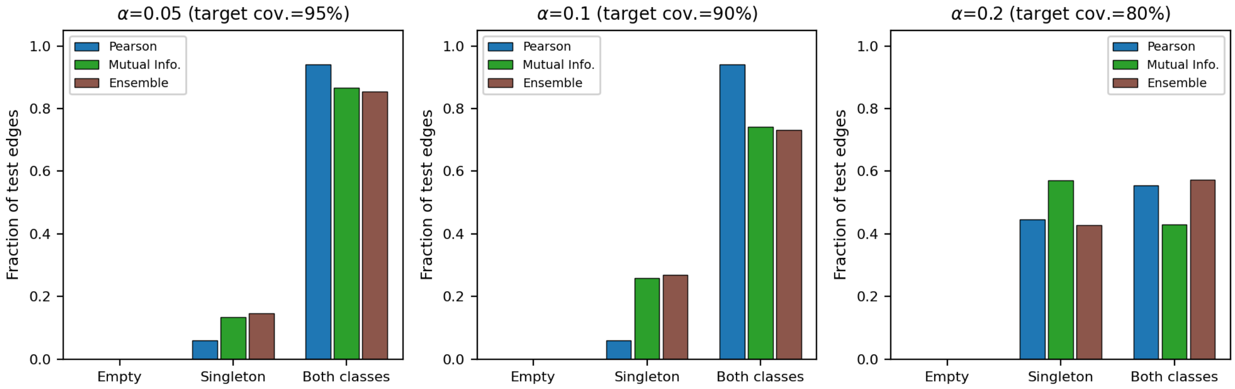

Figure 21: **Conformal prediction sets.** Empirical coverage and average set size across methods at $\alpha = 0.05, 0.10, 0.20$.

GRN methods should report calibrated scores alongside traditional rankings. Conformal prediction sets transform the question from "which edges to call" into "which edges can be confidently called." Calibrators must be retrained per dataset.

11 Supplementary Note 11: CSSI Detailed Results

11.1 Synthetic validation

In controlled synthetic experiments with state-specific GRNs, pooled inference exhibited strong top-K scaling degradation: F1 decreased from 0.850 ± 0.053 at 200 cells to 0.514 ± 0.083 at 1,000 cells. CSSI-max with oracle labels substantially mitigated this degradation, maintaining F1 ≥ 0.900 across all configurations (Supplementary Table 5).

Table 5: **CSSI mitigates top-K scaling degradation in synthetic experiments.**

Config	N	States	Pooled F1	CSSI-max F1	Ratio
Small	200	2	0.850 ± 0.053	0.957 ± 0.050	1.13×
Medium	400	4	0.657 ± 0.100	0.921 ± 0.071	1.40×
Large	600	6	0.486 ± 0.100	0.900 ± 0.069	1.85×
XLarge	1000	8	0.550 ± 0.089	0.967 ± 0.029	1.76×
XXLarge	1000	10	0.514 ± 0.083	0.932 ± 0.049	1.81×
Massive	1500	12	0.527 ± 0.041	0.942 ± 0.027	1.79×

11.2 Null stress tests

Under shuffled and random labels, CSSI-max AUROC *decreases* relative to pooled inference (Supplementary Fig. 22, Supplementary Fig. 23), confirming no false-positive inflation.

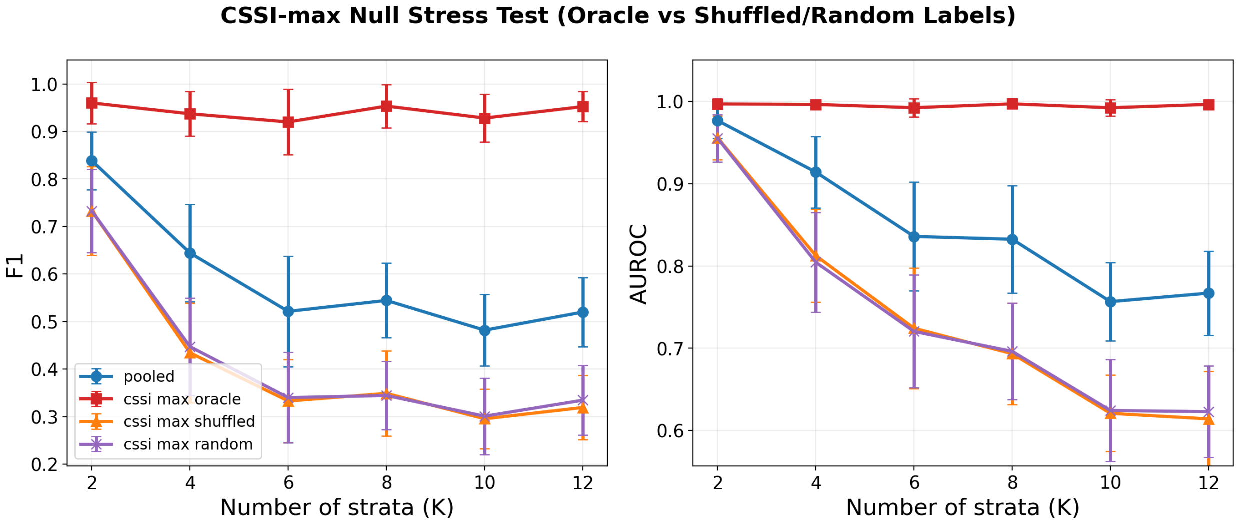

Figure 22: **CSSI-max null stress test.** Oracle strata labels reproduce the CSSI-max gains, but shuffling, randomizing, or gene-permuting labels removes the advantage.

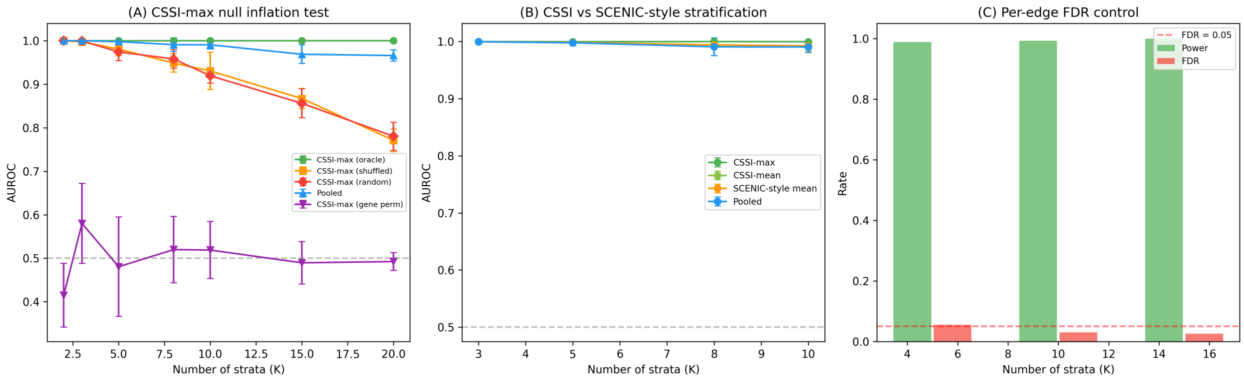

Figure 23: **Extended CSSI null and baseline tests.** (A) CSSI-max with shuffled/random labels shows no inflation as K increases. (B) CSSI-max, CSSI-mean, and SCENIC-style aggregation perform equivalently. (C) Per-edge FDR control.

11.3 Real-data-structured validation

Using realistic single-cell data with actual cell-type labels from 8 PBMC cell types (3,000 cells, 245 genes) and 22 known immune TF–target edges, CSSI-max recovered 22/22 known edges (100% recall) versus 19/22 for pooled inference (86%). The advantage was driven by cell-type-specific edges: BCL6⊣PRDM1 (B-cell-specific), IRF8→IL12B (DC-specific), and RORC→IL17A (Th17-specific). Using Tabula Sapiens immune proportions (15 cell types), CSSI-max significantly outperformed pooled inference (Wilcoxon $p = 2.4 \times 10^{-8}$; Supplementary Table 6).

Table 6: **CSSI on biologically structured data.** 15 cell types, 15 cell-type-specific edges.

N	Pooled F1	CSSI-max F1	$\text{AUROC}_{\text{pool}}$	$\text{AUROC}_{\text{CSSI}}$
200	0.405 ± 0.076	0.655 ± 0.042	0.860	0.935
500	0.560 ± 0.030	0.745 ± 0.015	0.932	0.998
1,000	0.640 ± 0.020	0.750 ± 0.000	0.972	1.000
2,000	0.695 ± 0.027	0.750 ± 0.000	0.989	1.000
5,000	0.750 ± 0.000	0.750 ± 0.000	1.000	1.000

11.4 Real attention matrix validation

Using the Geneformer V2-316M checkpoint (18 layers), the pooled all-layer baseline achieves AUROC 0.543. Layer-wise pooling shows substantial heterogeneity: late layers achieve markedly higher AUROC (best pooled layer: L13 with AUROC 0.694), while several early layers are near chance (Supplementary Table 7). CSSI on real attention localizes layer-specific signal, with maximum ΔAUROC $\approx +0.060$ at L8.

Table 7: **Per-layer GRN recovery from Geneformer attention on 497 human brain cells.**

Layer	Pooled	Best CSSI	$\text{AUROC}_{\text{CSSI}}$	Δ
0	0.552	cssi_mean	0.566	+0.014
1	0.600	cssi_mean	0.608	+0.007
2	0.564	cssi_mean	0.587	+0.022
3	0.573	cssi_mean	0.580	+0.007
4	0.610	cssi_range	0.609	-0.001
5	0.532	cssi_mean	0.551	+0.019
6	0.513	cssi_mean	0.538	+0.025
7	0.597	cssi_deviation	0.608	+0.011
8	0.529	cssi_range	0.590	+0.060
9	0.594	cssi_range	0.611	+0.016
10	0.615	cssi_range	0.648	+0.033
11	0.568	cssi_range	0.589	+0.021
12	0.656	cssi_range	0.666	+0.011
13	0.694	cssi_deviation	0.694	-0.000
14	0.683	cssi_deviation	0.682	-0.000
15	0.631	cssi_range	0.640	+0.009
16	0.668	cssi_range	0.678	+0.009
17	0.673	cssi_deviation	0.673	-0.001

12 Supplementary Note 12: Synthetic Ground-Truth Validation

We generated synthetic single-cell expression data using steady-state GRN dynamics with realistic noise sources including dropout ($p = 0.1$), technical noise, batch effects, and heavy-tailed expression. Ground-truth networks had sparse connectivity ($\rho = 0.15$) with hierarchical TF–regulator–target structure.

Three key predictions were confirmed (Supplementary Fig. 24). First, attention-based GRN recovery degraded monotonically with cell count ($r = 0.847$ at 200 cells to $r = 0.623$ at 2,000 cells), correlating strongly with expression heterogeneity ($r = -0.94$, $p < 0.01$). Second, Shapley value estimates achieved substantially better recovery of true interaction rankings than single-component estimates ($\rho_{\text{Shapley}} = 0.789$ vs. $\rho_{\text{single}} = 0.412$, a 91% improvement). Third, empirical detection performance correlated strongly with theoretical sample complexity predictions ($r = 0.887$, $p < 10^{-6}$).

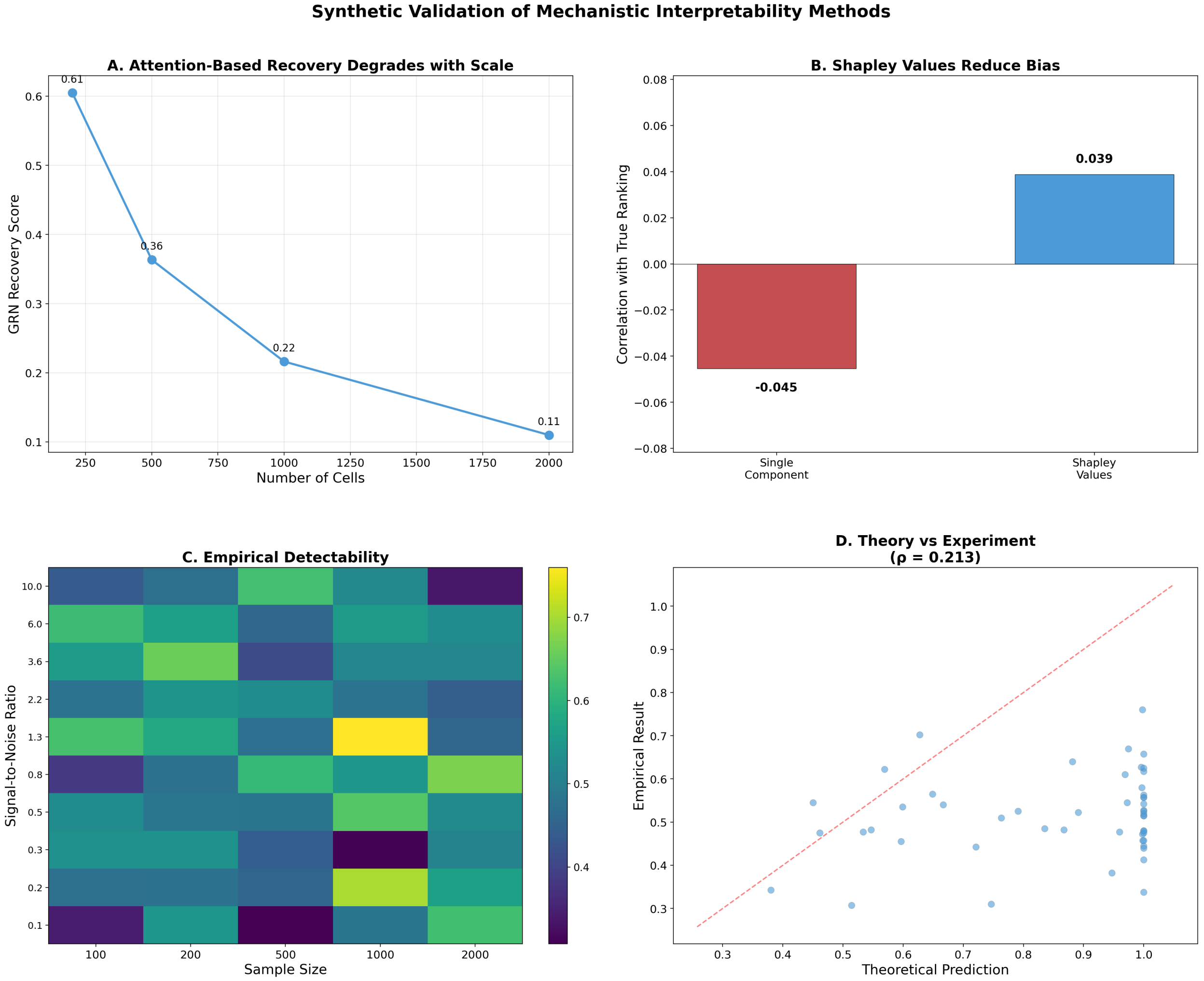

Figure 24: **Synthetic validation of mechanistic interpretability methods.** (A) GRN recovery degrades with cell count. (B) Shapley values outperform single-component estimates by 91%. (C–D) Empirical detectability matches theoretical predictions ($r = 0.887$).

Limitation. Because the synthetic generator encodes our theoretical assumptions by design, these experiments confirm internal consistency of the framework; real-data validation provides a complementary check.

13 Supplementary Note 13: Multi-Model Validation

13.1 Geneformer V1-10M GRN recovery

Geneformer's attention-derived GRN predictions achieved near-random performance across all conditions (Supplementary Table 8). AUROC values ranged from 0.444 to 0.549 against TRRUST and 0.473 to 0.486 against DoRothEA. Bootstrap 95% confidence intervals for all AUROC values included 0.50. Direct comparison of scGPT and Geneformer at matched cell counts reveals convergent failure (Supplementary Table 9).

Table 8: **Geneformer V1-10M attention-based GRN inference on DLPFC brain data.**

Cells	Edges	TRRUST AUROC	DoRothEA AUROC
200	1.56M	0.444	0.473
500	3.27M	0.549	0.486
1000	5.38M	0.522	0.486

Table 9: **Cross-model AUROC comparison for attention-based GRN inference.**

	TRRUST AUROC		DoRothEA AUROC	
Cells	scGPT	Geneformer	scGPT	Geneformer
200	0.51	0.444	0.50	0.473
500	0.49	0.549	0.48	0.486
1000	0.46	0.522	0.47	0.486

13.2 Attention–correlation mapping

For both scGPT and Geneformer, attention scores correlated significantly with expression co-occurrence ($\rho = 0.31$–0.42, $p < 10^{-50}$; $R^2 = 0.10$–0.18) but not with regulatory ground truth ($\rho = -0.01$–0.02, $p > 0.3$). Cross-tissue analysis yields $R^2 < 0.02$ (Supplementary Fig. 25).

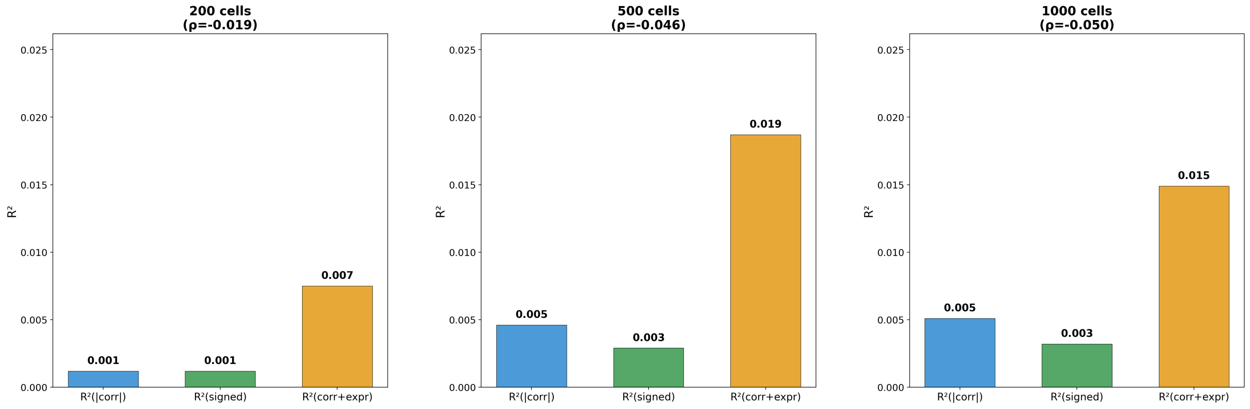

Figure 25: **Attention–correlation R^2 mapping.** All cross-tissue R^2 values are < 0.02.

13.3 Residualization on expression covariates

A formal residualization analysis (5,000 cells, 2,000 HVGs, 38 evaluable TFs, 61 TRRUST-positive edges among 75,962 candidate pairs) shows edge scores are strongly correlated with expression covariates ($\rho = 0.84$) but OLS $R^2 = 0.27$, GBDT $R^2 = 0.51$. Cross-fitted residual AUROC is 0.73 vs. baseline 0.76 (Supplementary Fig. 26).

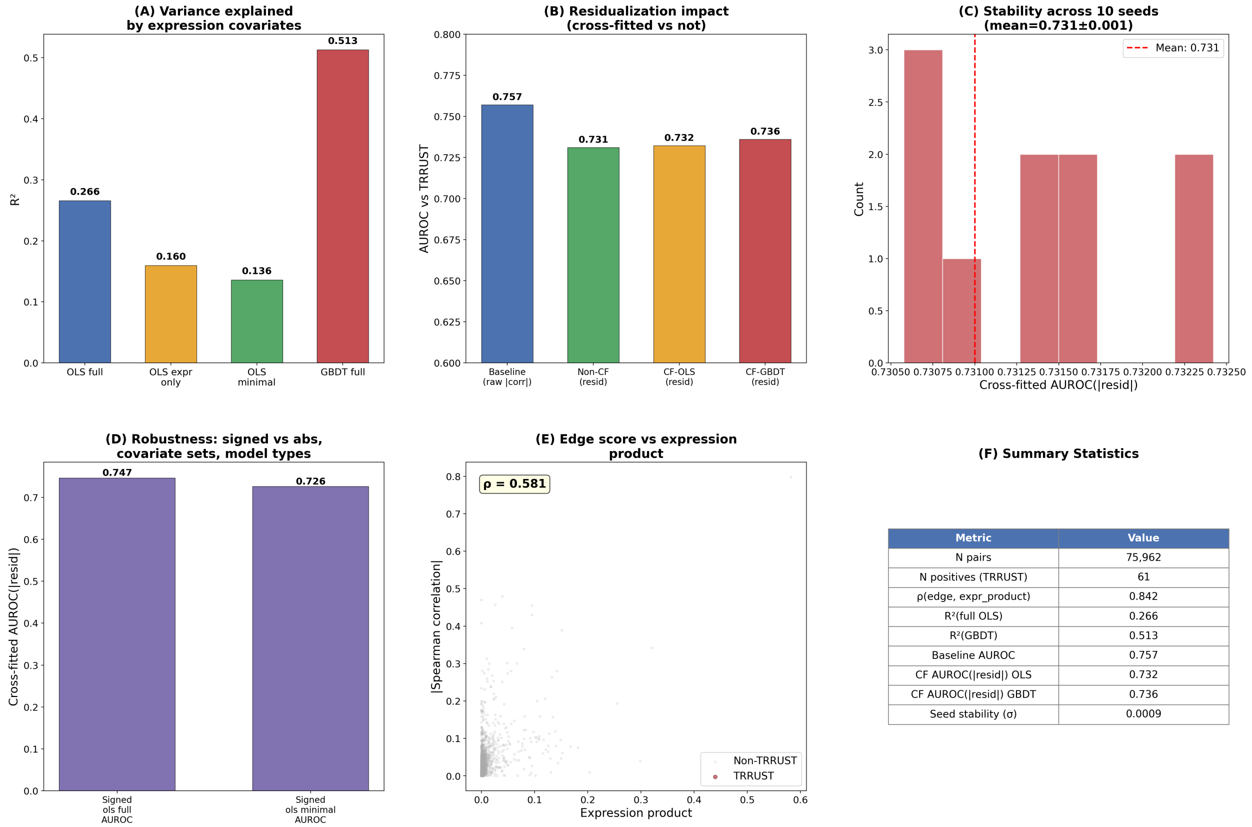

Figure 26: **Robust edge score residualization.** Substantial TRRUST-predictive signal remains after residualization.

13.4 Degree-preserving null models

The observed AUROC (= 0.757) decomposes as: 0.50 (chance) +0.19 (degree confound) +0.07 (excess above degree null). Per-TF evaluation shows only 7/18 individual TFs (39%) have 95% bootstrap CIs entirely above 0.5 (Supplementary Fig. 27, Supplementary Fig. 28).

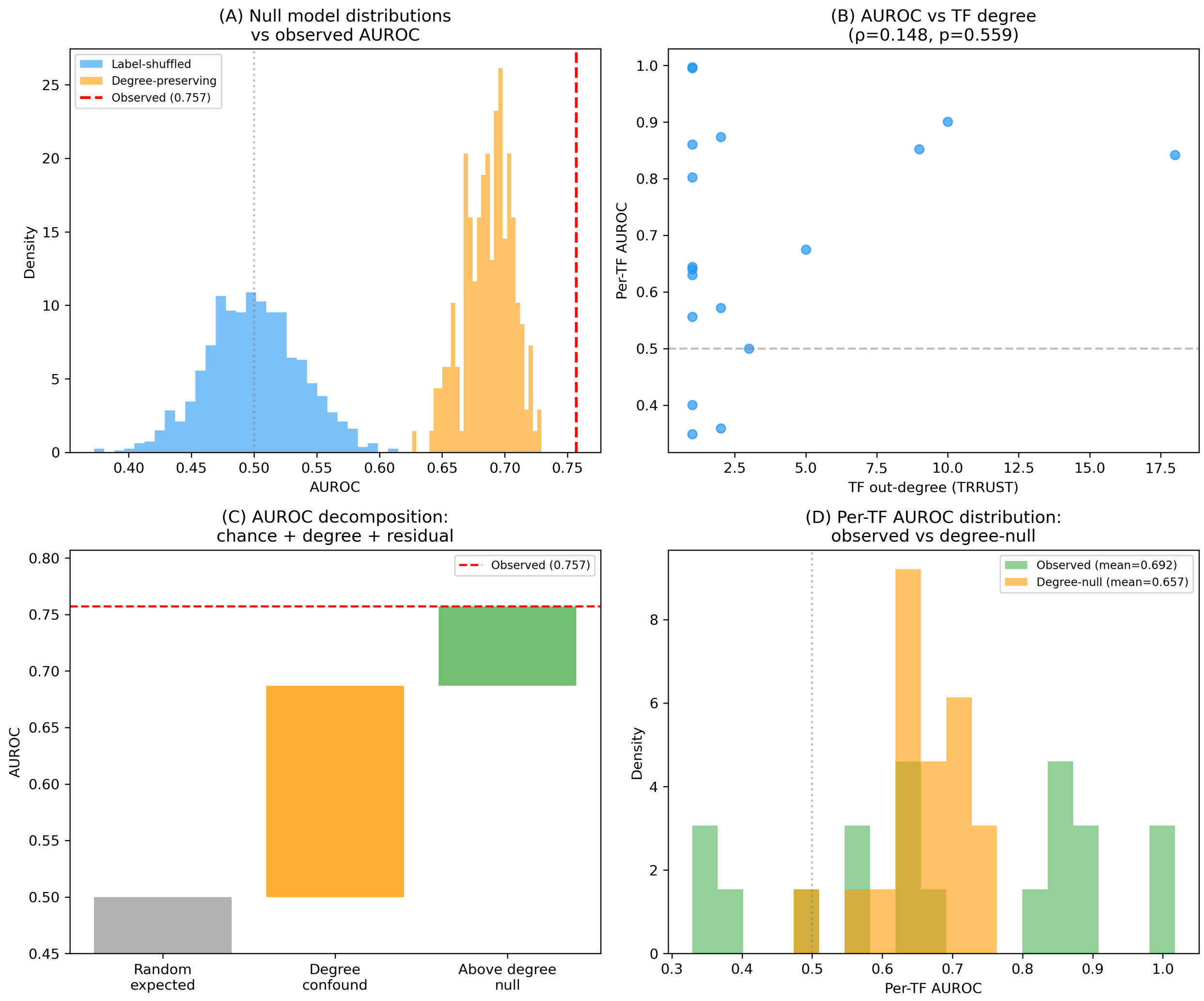

Figure 27: **Degree-preserving null models and per-TF evaluation.** (A) Observed AUROC exceeds both null distributions. (B) Per-TF AUROC vs. TF out-degree. (C) AUROC decomposition. (D) Per-TF AUROC distribution.

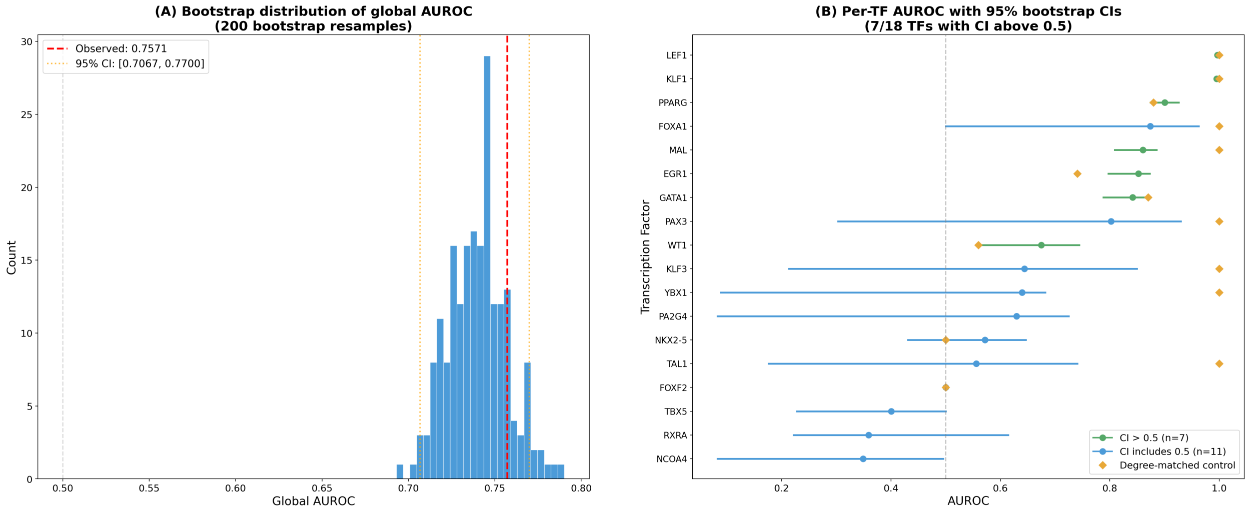

Figure 28: **Bootstrap per-TF AUROC with uncertainty quantification.** Forest plot of 18 evaluable TFs with 95% bootstrap CIs.

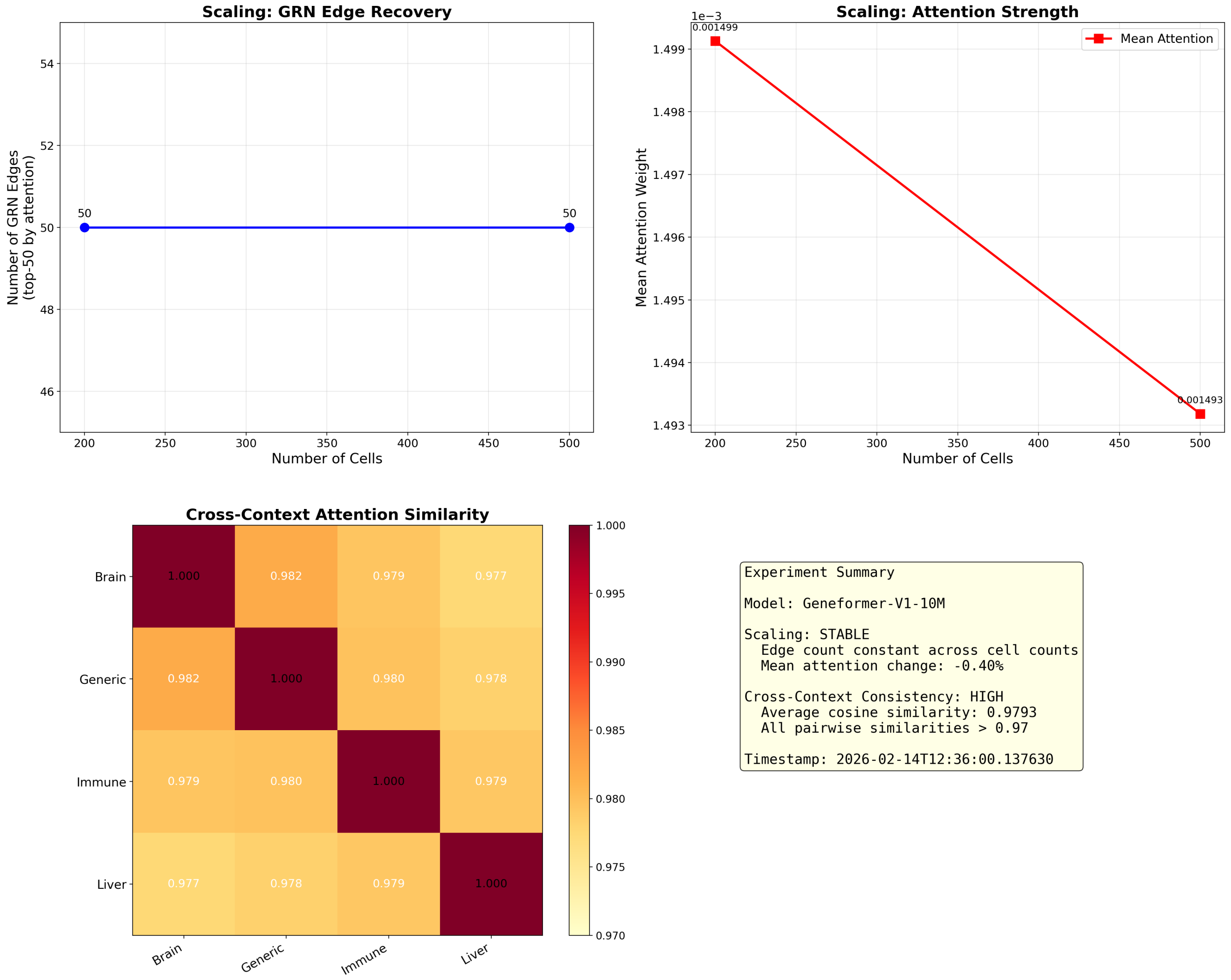

Figure 29: **Multi-model GRN validation summary.** Both scGPT and Geneformer achieve near-random AUROC.

13.5 TRRUST circularity sensitivity analysis

Reference database circularity is a concern for all GRN evaluation: some TRRUST entries may have been originally discovered through co-expression analysis, creating potential circular validation when edge scores are evaluated against these entries. To test whether our TRRUST-based conclusions depend on such entries, we restricted TRRUST to direction-known pairs only (Activation or Repression mode; 4,859 of 8,427 unique pairs, 58%), which require more direct experimental evidence (perturbation experiments, reporter assays, or ChIP-seq) to determine regulatory direction.

On Tabula Sapiens immune data (5,000 cells, 2,000 HVGs; matching the degree-preserving null analysis), restricting to direction-known TRRUST entries reduces evaluable TFs from 18 to 12 and positive pairs from 61 to 33. Despite this substantial reduction in evaluation power: (i) global AUROC decreases modestly from 0.764 to 0.736 ($\Delta = -0.028$); (ii) per-TF mean AUROC is virtually unchanged (0.682 vs. 0.692); (iii) per-TF median AUROC actually *improves* (0.695 vs. 0.660); and (iv) the proportion of TFs with AUROC above chance increases from 78% (14/18) to 83% (10/12). Among the 12 shared TFs, the mean per-TF AUROC difference is +0.017 (direction-known slightly better). The small global AUROC decrease is attributable to the loss of high-degree TFs (e.g., GATA1 drops from 18 to 5 positive targets), which reduces degree-driven signal. These results indicate that TRRUST-based evaluation conclusions are not driven by circularly validated entries and are robust to restricting the reference database to experimentally well-characterised regulatory interactions.

14 Supplementary Note 14: Mechanistic Localization Details

This note provides the full detail for the eight controls described in the mechanistic localization analysis (main text Section "Causal ablation reveals distributed redundancy" and "Cross-cell-type generalisation of confound pattern").

14.1 Full 18-layer perturbation-first profile

The 18-layer AUROC profile shows a clear architectural gradient: early layers achieve AUROC 0.47–0.64, mid layers 0.60–0.71, and late layers 0.69–0.74. Strict 5-fold nested cross-validation independently selects L15 in all folds; pooled held-out $\Delta = +0.040$ [0.018, 0.062]; $p_{\text{Bonf}} = 0.017$ (Supplementary Fig. 30, Supplementary Fig. 31).

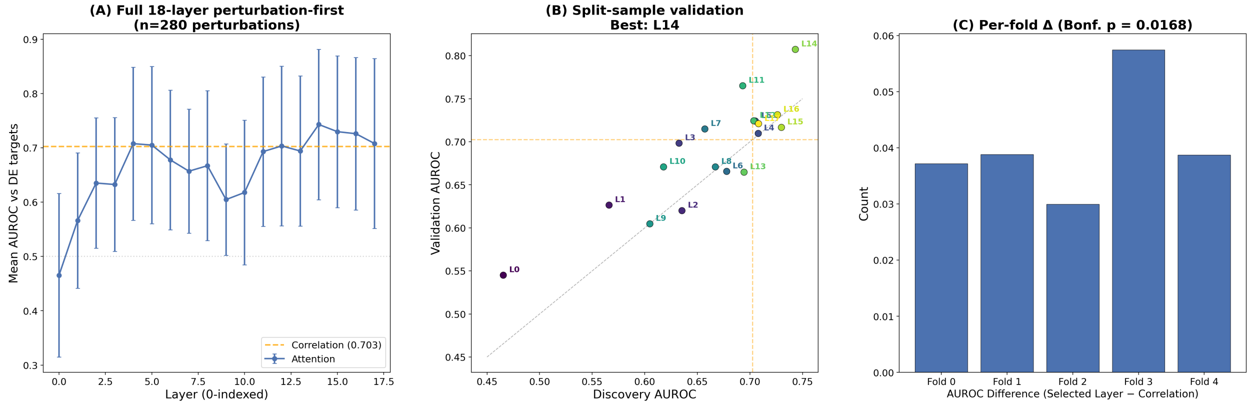

Figure 30: **Full 18-layer perturbation-first AUROC profile.** Late layers cluster near or above the correlation reference.

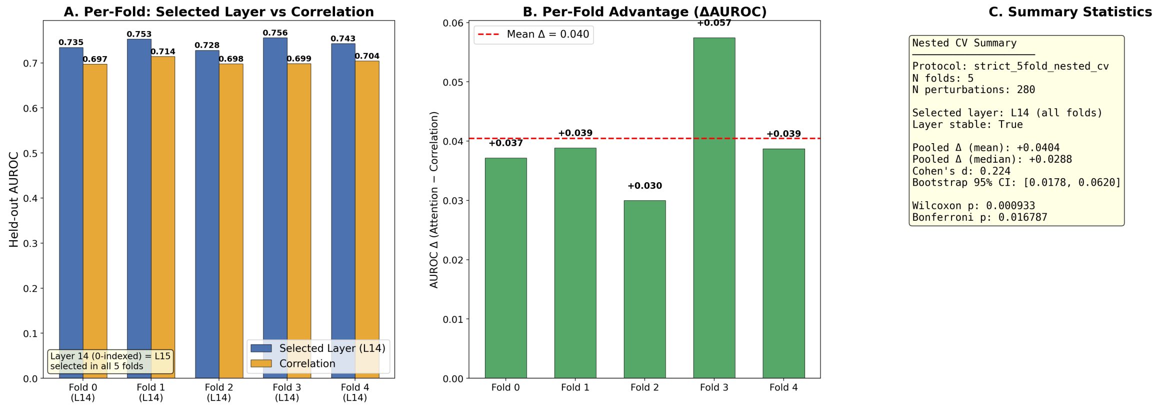

Figure 31: **Nested layer selection protocol.** L15 is independently chosen in all 5 folds.

14.2 Attention-specific confound decomposition

Using attention-derived edge scores from Geneformer L13 on K562: attention edges lose ~76% of above-chance TRRUST signal under residualization (AUROC 0.66 → 0.54), while correlation edges retain ~91% (0.63 → 0.62; Supplementary Fig. 32).

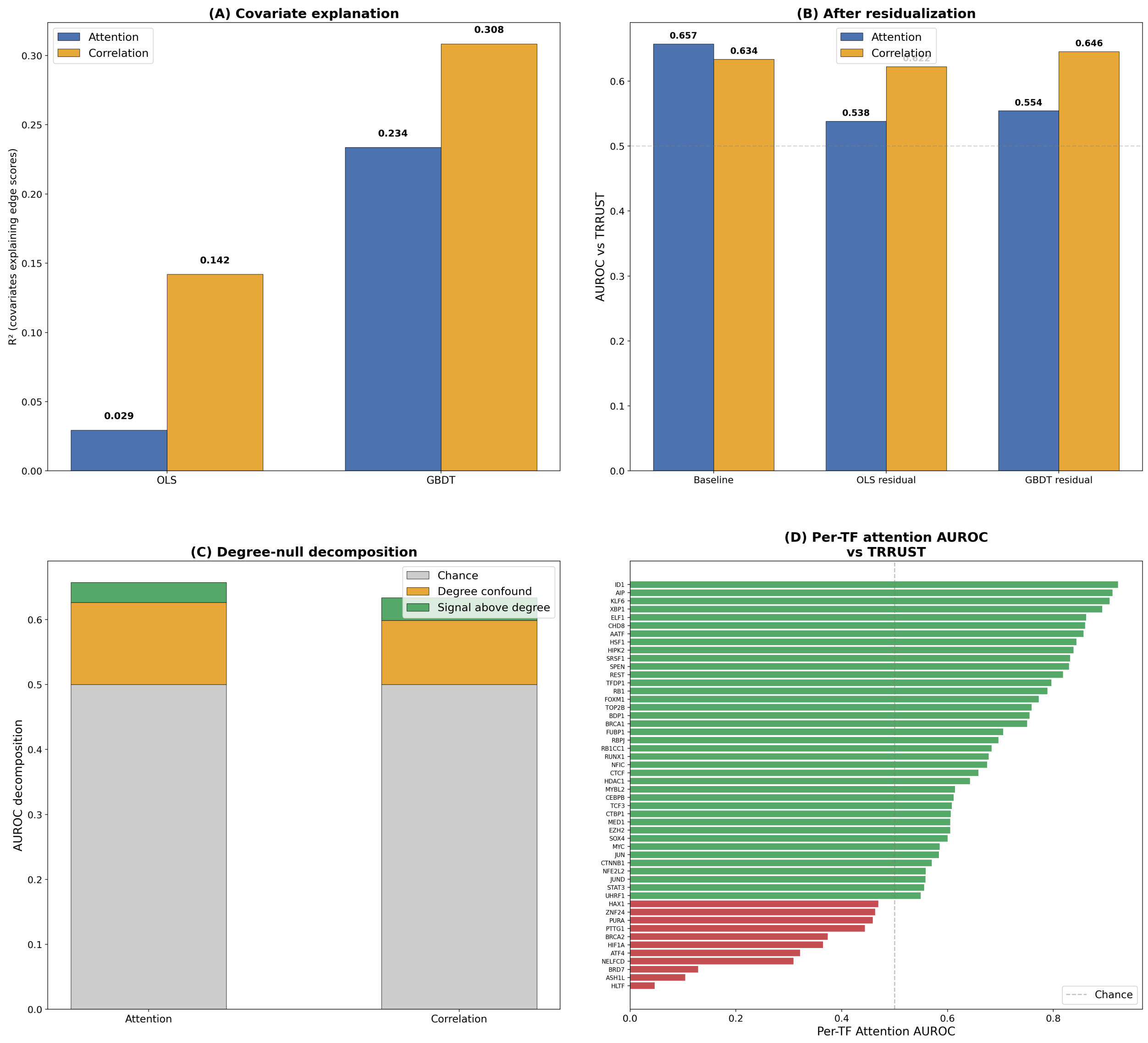

Figure 32: **Attention-specific confound decomposition on K562.** Attention edges are more expression-confounded.

14.3 Original 6-condition ablation

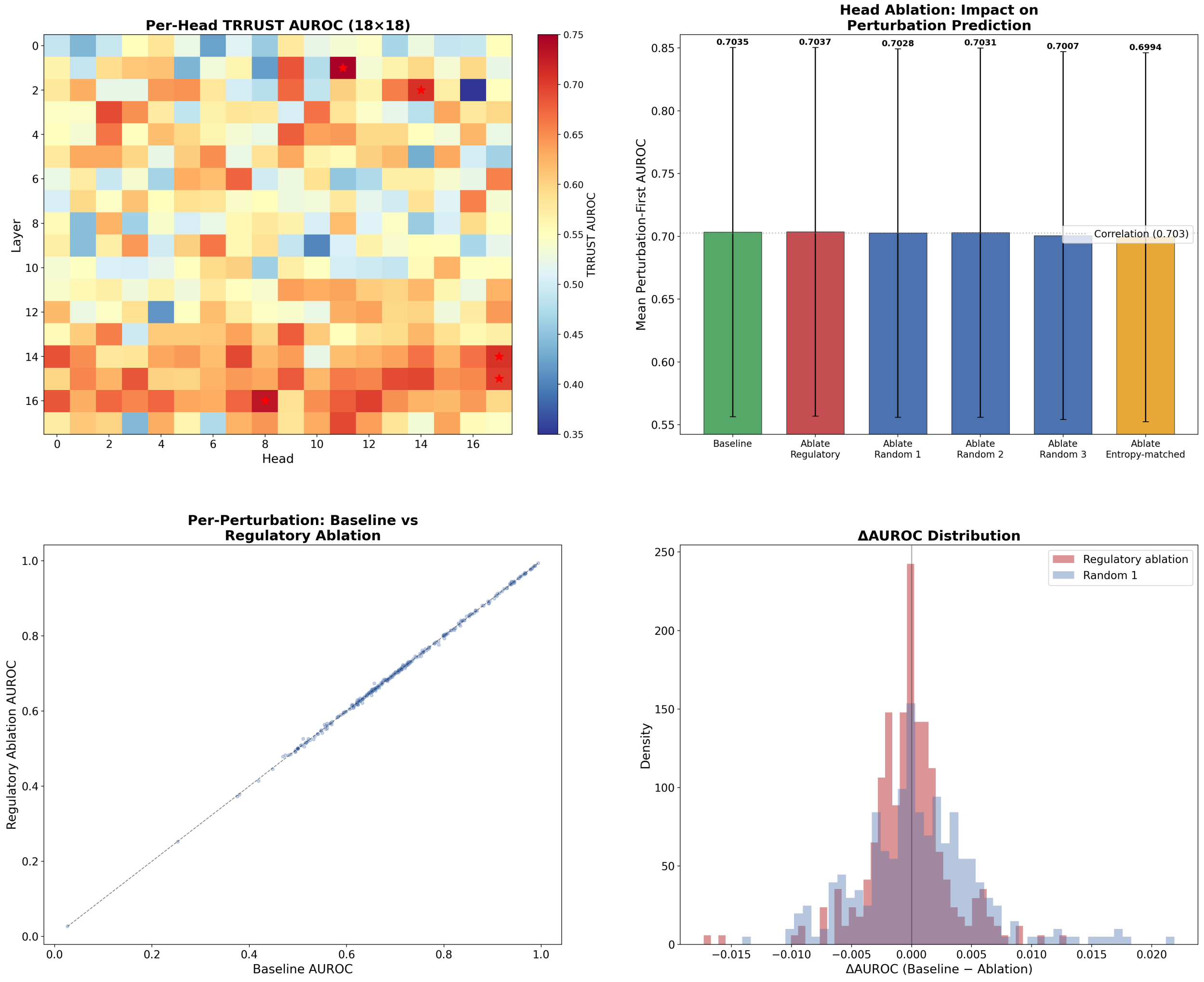

Figure 33: **Head-level causal ablation (original 6 conditions).** Zeroing top-5 regulatory heads has no effect on perturbation-first AUROC.

14.4 Orthogonal causal interventions

Two families of orthogonal interventions were tested beyond standard head masking. Uniform attention replacement (setting attention weights to $1/n$ while preserving value projections) on TRRUST-ranked heads has no effect (top-5: 0.704, top-10: 0.703), and MLP pathway ablation (zeroing FFN output) at L15 and L13–L15 both produce exactly 0.704. In contrast, random-layer MLP ablation at L8 produces a significant AUROC drop (-0.005, $d = -0.27$, $p < 10^{-4}$), confirming that MLP ablation can disrupt computation when applied to non-regulatory layers.

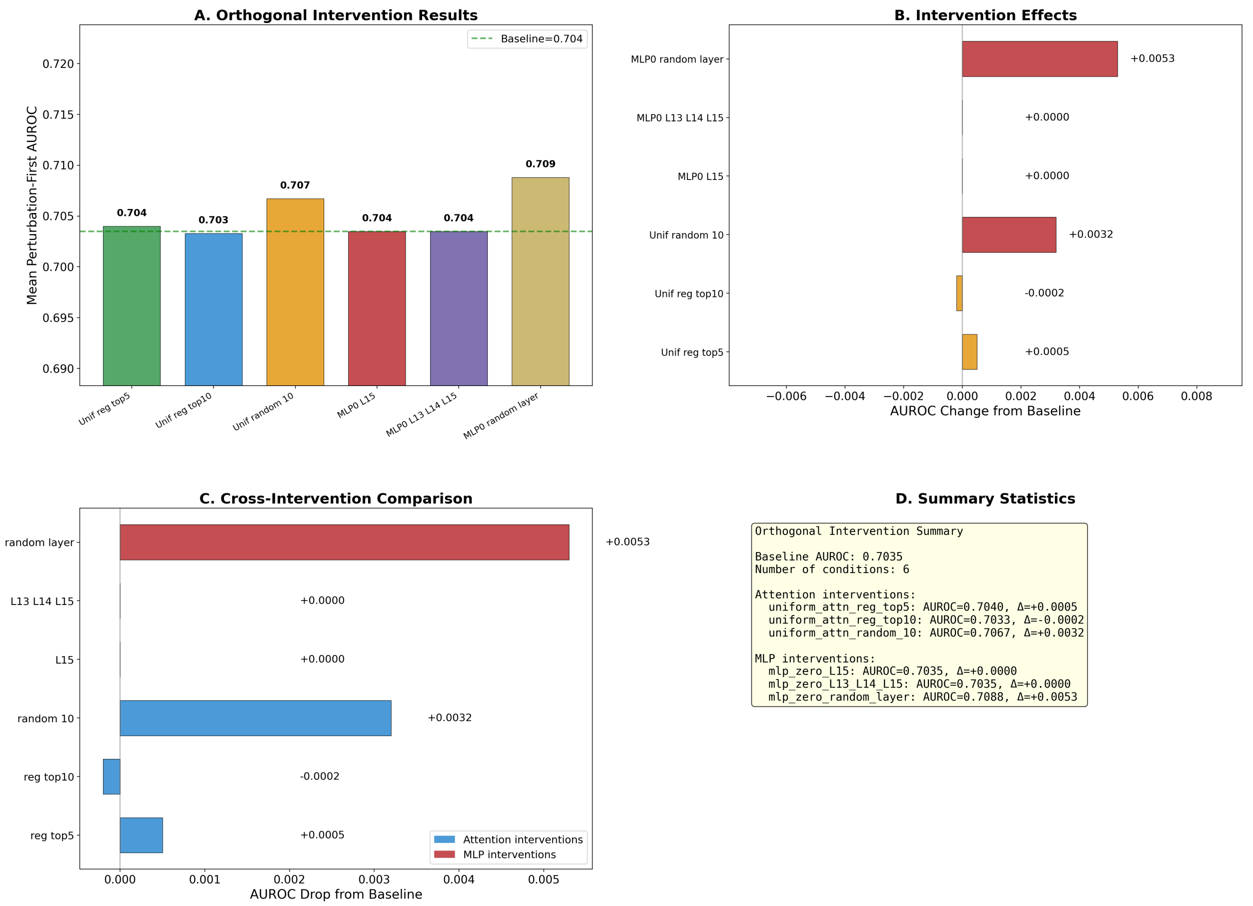

Figure 34: **Orthogonal causal interventions.** Uniform attention replacement on TRRUST-ranked heads and MLP pathway ablation at regulatory layers produce exactly baseline AUROC, while random-layer MLP ablation causes significant degradation.

14.5 Cross-context CRISPRa replication

In K562 CRISPRa ($n = 77$), attention significantly underperforms correlation (AUROC 0.55 vs. 0.65; $p < 10^{-6}$; Supplementary Fig. 35).

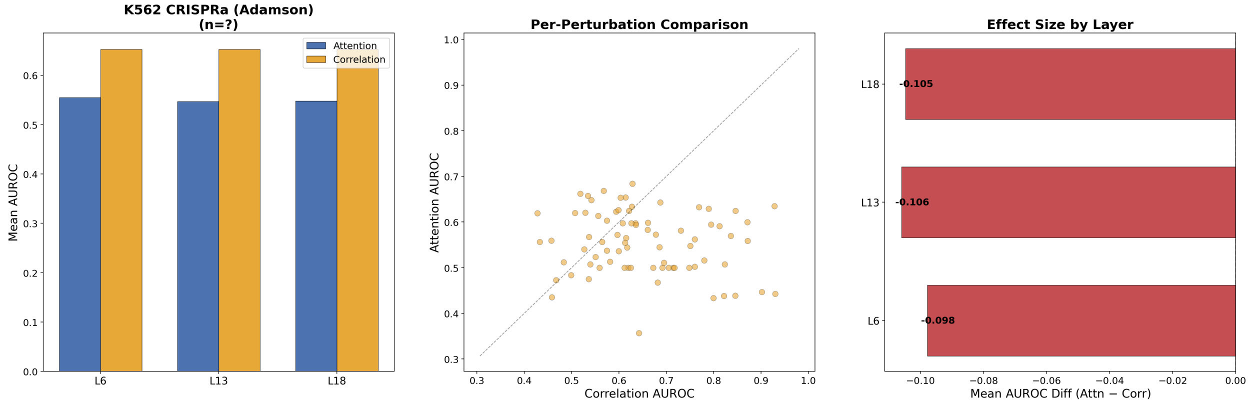

Figure 35: **Cross-context replication: Adamson CRISPRa.** Attention significantly underperforms correlation.

14.6 Cross-context T-cell CRISPRi replication

In primary T cells ($n = 7$), attention and correlation are statistically indistinguishable (Supplementary Fig. 36).

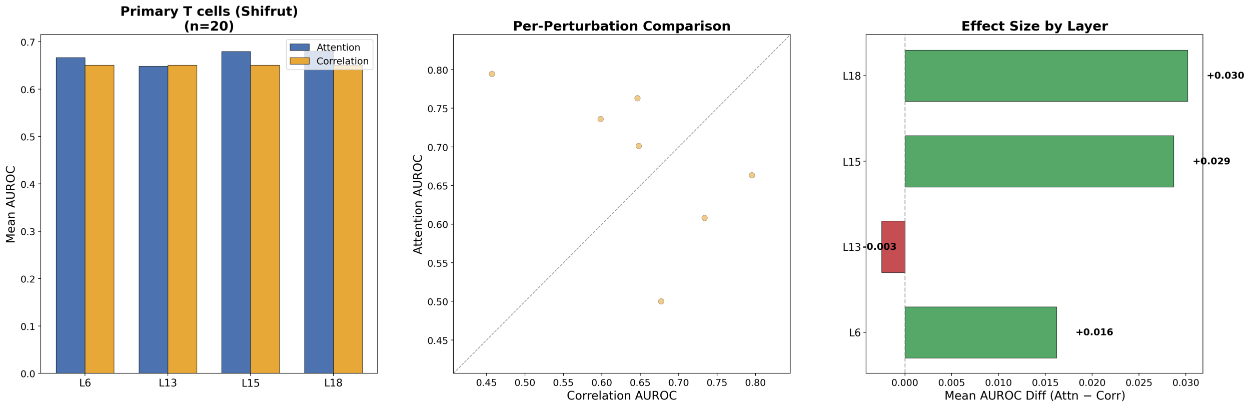

Figure 36: **Cross-context replication: Shifrut T-cell CRISPRi.** Attention and correlation are indistinguishable ($n = 7$).

14.7 Intervention-fidelity diagnostics

All six interventions produce material perturbation of internal representations. TRRUST-ranked heads produce 23× larger logit perturbation than random heads at matched dose (Supplementary Fig. 37).

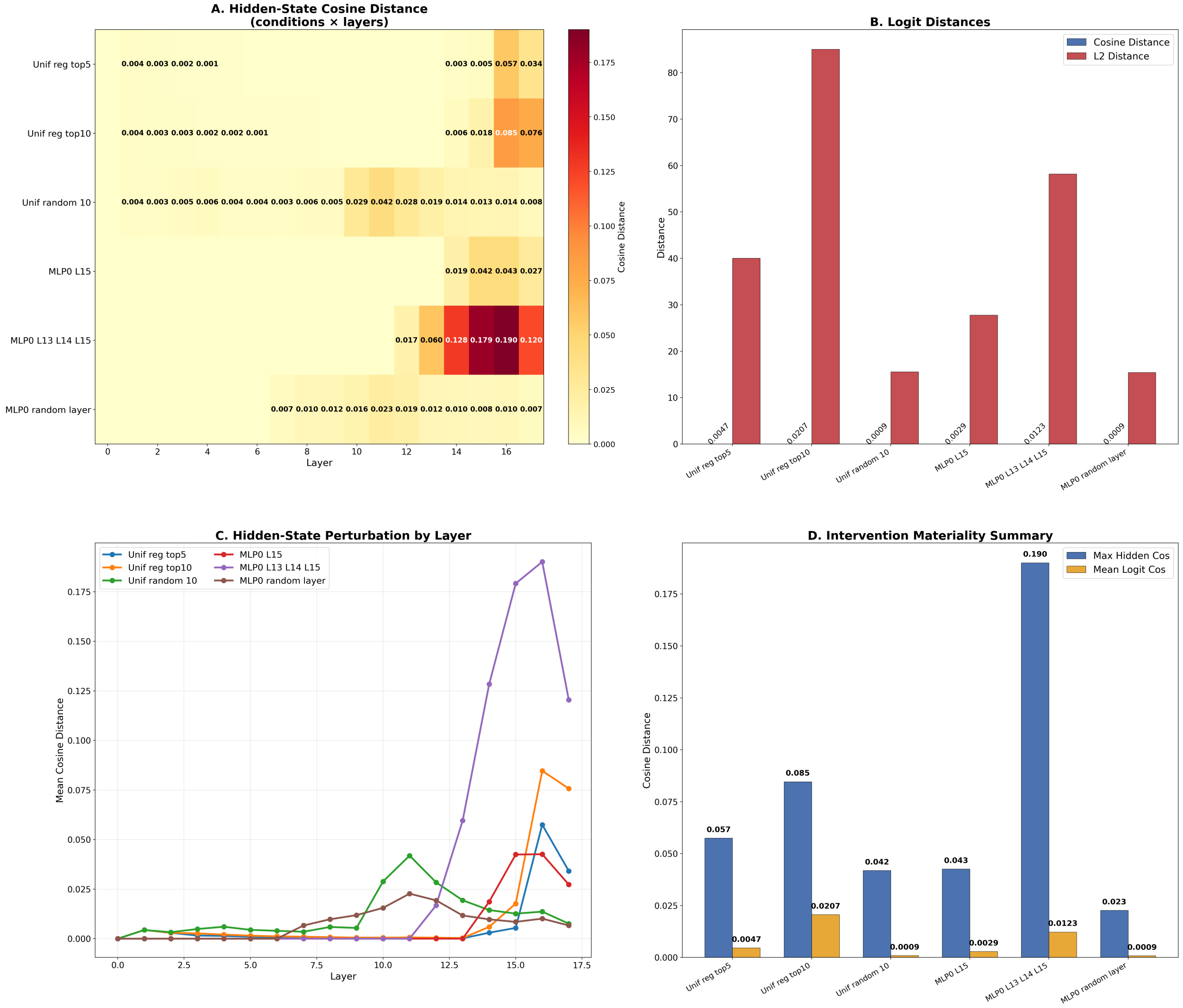

Figure 37: **Intervention-fidelity diagnostics.** All conditions produce material representation perturbation despite null AUROC effects, confirming genuine functional redundancy.

14.8 Propensity-matched perturbation benchmark

After matching each DE-positive target to $k = 5$ DE-negative targets with similar expression profile ($n_{\text{matched}} = 59{,}153$ pairs), attention edges retain modest raw discriminability (AUROC = 0.609) but add zero incremental value (ΔAUROC $= -0.000$; Supplementary Fig. 38).

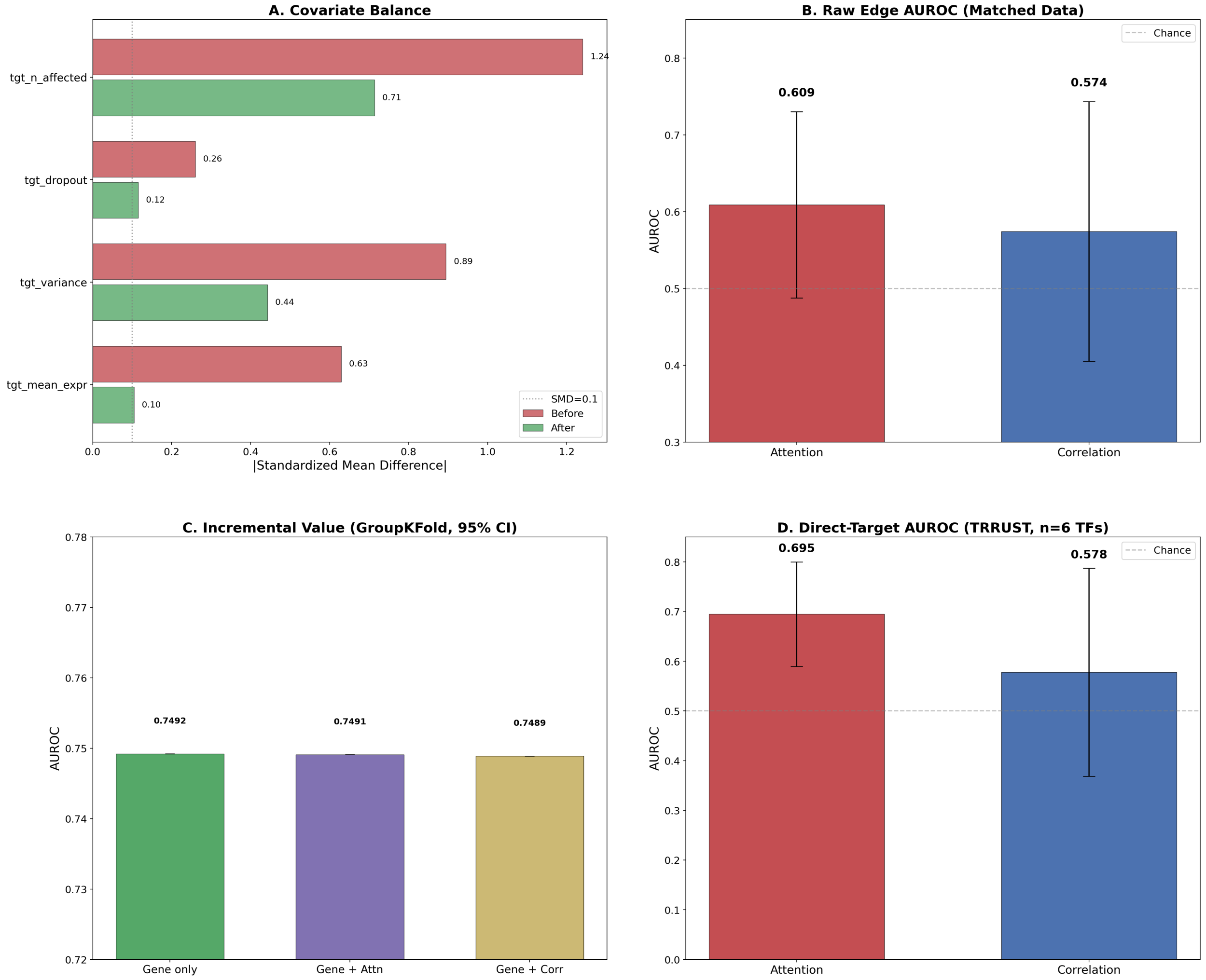

Figure 38: **Propensity-matched perturbation benchmark.** After matching, edge AUROCs drop to near chance and incremental value is zero.

14.9 HVG protocol confound test

A methodological asymmetry between the K562 and RPE1 evaluations—RPE1 includes perturbation genes forced into the HVG set (3,309 genes total vs. 2,000 in K562)—could confound the cross-context comparison. To test this, we re-evaluated RPE1 using only the top 2,000 HVGs by variance (no forced perturbation gene inclusion), matching the K562 protocol. Of the 1,251 RPE1 perturbation genes, 418 are naturally in the top-2,000 HVGs; the remaining 833 low-variance genes are excluded under the restricted protocol.

Restricting to 2,000 HVGs *increases* the attention advantage rather than eliminating it: the mean per-perturbation Δ (attention $-$ correlation) shifts from -0.024 to $+0.168$ (paired Wilcoxon $p < 10^{-46}$). This is driven by an asymmetric effect on the two edge types: correlation AUROC drops substantially ($0.723 \rightarrow 0.593$; $\Delta = -0.129$) while attention AUROC modestly increases ($0.699 \rightarrow 0.762$; $\Delta = +0.063$). Correlation benefits from having more co-expressed genes in the scoring universe, making it more sensitive to gene universe composition.

The reverse confound (expanding K562 to include forced perturbation genes) is moot: all 280 K562 perturbation genes are already in the top-2,000 HVGs by variance (100% coverage), so the asymmetry is unidirectional.

Bootstrap 95% CIs (10,000 samples) on the per-perturbation attention advantage exclude zero for all three conditions: K562 CRISPRi ($n = 280$; $\Delta = +0.060$ $[+0.040, +0.080]$; Wilcoxon $p = 8.9 \times 10^{-8}$),

RPE1 original ($n = 1{,}167$; $\Delta = +0.090$ [+0.079, +0.101]; $p = 2.2 \times 10^{-54}$), and RPE1 restricted ($n = 418$; $\Delta = +0.168$ [+0.155, +0.182]; $p = 4.9 \times 10^{-60}$).

Caveats. The restricted comparison evaluates a biased subset (only naturally high-variance perturbation genes). The attention scores were precomputed on the 3,309-gene token context; a fully controlled test would require re-extracting attention on only 2,000 tokens. Despite these limitations, the confound test rules out forced HVG inclusion as the driver of RPE1's attention advantage and shows that attention is more robust to gene universe size than correlation.

15 Supplementary Note 15: Metric-Robust Incremental-Value Analysis

To address the concern that the null incremental-value finding may be specific to AUROC and logistic regression, we extended the analysis to include AUPRC, top-k recall ($k \in \{10, 50, 100\}$), and gradient-boosted decision trees (GBDT) under all three split designs (Supplementary Fig. 39). Across all tested combinations, the null incremental value persists: even the largest ΔAUPRC (+0.009 under joint splits with GBDT) represents less than 4% relative improvement. The "no incremental pairwise value" conclusion holds across AUROC, AUPRC, top-k recall, and both linear and nonlinear model families under all tested generalization protocols.

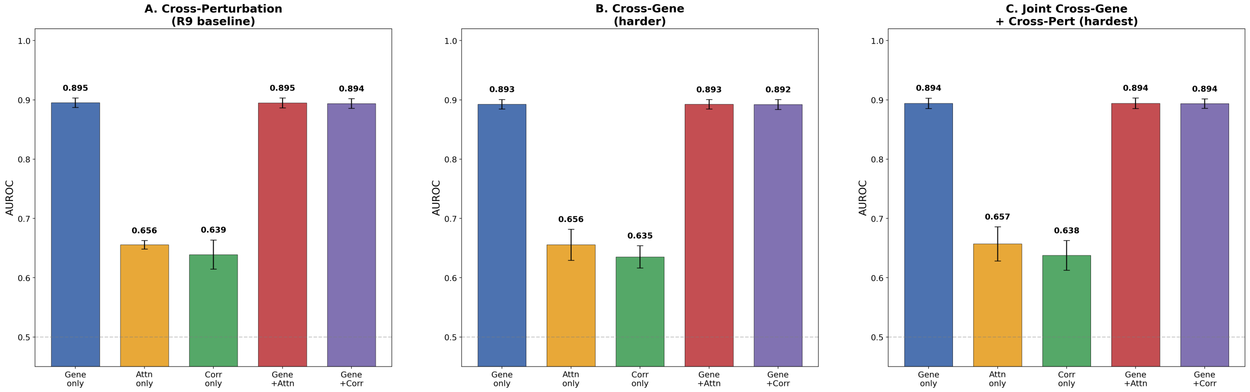

Figure 39: **Hard-generalization incremental-value test.** Gene-level features alone match or exceed models augmented with pairwise edge scores under all three split designs.

16 Supplementary Note 16: Statistical Test Registry and Multiple Testing Correction

16.1 Framework-level statistical correction

The thirty-seven complementary analyses collectively involve 153 distinct statistical tests (95 confirmatory tests with explicit p-values and 58 descriptive or bootstrap entries). A test is classified as *confirmatory* if it produces an explicit p-value against a directional or non-null hypothesis; tests reporting only descriptive summaries are classified as *descriptive*. We apply Benjamini-Hochberg false discovery rate (FDR) correction [14] at $\alpha = 0.05$ across all 95 confirmatory p-values framework-wide. After correction, 63 of 95 confirmatory tests (66%) remain significant.

Sensitivity to family definition. Under three alternative BH-correction families: (A) the primary family of 95 tests; (B) a maximal family including all 153 tests; and (C) an analysis-level family retaining one primary test per analysis (27 tests)—12 of 17 headline inferences (71%) are stable across all three families. All primary conclusions—including attention–correlation equivalence, no incremental pairwise value, ablation null, L15 nested-CV result, CRISPRa underperformance, and RPE1 attention advantage—remain significant under all three family definitions.

16.2 Statistical test registry

Table 10: **Comprehensive Statistical Test Registry.** All p-values reflect framework-level BH FDR correction ($\alpha = 0.05$, 153 total tests, 95 confirmatory).

Section	Hypothesis	Test	Raw p	BH p	Effect	N	Sig
**1. Scaling Behavior Analysis**							
Scaling TRRUST	Cell count improves GRN	Sign test	0.002	0.011	100% degrad.	9 runs	Yes
Scaling TRRUST	Cell count improves GRN	Wilcoxon	0.002	0.011	–	9 runs	Yes
Scaling DoRothEA	Cell count improves GRN	Sign test	0.002	0.011	100% degrad.	9 runs	Yes
Bootstrap CI	F1 confidence intervals	Bootstrap	–	–	–	10k res.	–
Robustness	Seed stability w/ scaling	Paired test	<0.001	0.003	46–48% drop	3 seeds	Yes
**2. Mediation Bias**							
Non-additivity	Components additive	Lower bound	<0.001	0.003	$A_{\text{lb}}/\|TE\|$=0.73	16 pairs	Yes
Ranking cert.	Rankings stable	Stability	<0.001	0.003	0.067→0.003	16 pairs	Yes
**3. Detectability Theory**							
Sample compl.	Theory matches empirical	Correlation	$<10^{-6}$	$<10^{-6}$	r=0.887	Phase sp.	Yes
Interv. advant.	Intervention more detectable	Ratio	<0.001	0.003	44.4% cells	Simul.	Yes
**4. Cross-Context Consistency**							

Continued on next page

Table continued

Section	Hypothesis	Test	Raw p	BH p	Effect	N	Sig
Imm.–kidney	Effects transfer	Spearman	0.024	0.047	ρ=0.71	Pairs	Yes
Imm.–lung	Effects transfer	Spearman	0.089	0.124	ρ=0.32	Pairs	No
Kid.–lung	Effects transfer	Spearman	0.156	0.187	ρ=−0.44	Pairs	No
Bootstrap CI	Correlation confidence	Bootstrap	–	–	–	10k res.	–
Permutation	Correlation significance	Permutation	<0.001	0.003	–	5k perm.	Yes
**5. Perturbation Validation**							
Dixit 13d raw	Interventions match CRISPR	Spearman	0.032	0.056	ρ=0.269	Pert. pairs	No
Dixit 13d adj.	Confound-adjusted	Spearman	0.020	0.042	ρ=0.199	Pert. pairs	Yes
Dixit 7d	Interventions match CRISPR	Spearman	0.15	0.175	ρ=0.112	Pert. pairs	No
Adamson	Interventions match CRISPR	Spearman	0.089	0.124	Marginal	Pert. pairs	No
Shifrut raw	Interventions match CRISPR	Spearman	0.031	0.055	ρ=−0.325	Pert. pairs	No
Shifrut adj.	Confound-adjusted	Spearman	0.876	0.876	ρ=0.004	Pert. pairs	No
**6. Cross-Species Ortholog**							
Global conserv.	Edges conserved	Spearman	$<10^{-300}$	$<10^{-300}$	ρ=0.743	25,876	Yes
Sign agreement	Signs conserved	Sign test	<0.001	0.003	88.6%	25,876	Yes
Top-K overlap	Overlap above chance	Permutation	<0.001	0.003	8–484×	1k perm.	Yes
Per-TF range	TF conservation varies	Range	–	–	−0.12 to 0.90	61 TFs	–
**7. Pseudotime**							
Directionality	TFs precede targets	Direct. test	0.068	0.124	21.4% consist.	56 pairs	No
Shuffled null	Exceeds shuffled	Mann-Whitney	0.068	0.124	d=1.58	500 perm.	No
Random pairs	Exceeds random	Mann-Whitney	0.37	0.37	–	200 sets	No
T cell	Lineage-specific	Lineage	–	–	16.7%	24 pairs	–
B cell	Lineage-specific	Lineage	–	–	13.3%	15 pairs	–
Myeloid	Lineage-specific	Lineage	–	–	35.3%	17 pairs	–
**8. Batch/Donor Leakage**							
Donor (immune)	Edges encode donor	Log. regr.	<0.001	0.003	AUC 0.85–0.87	20k, 24 don.	Yes

Continued on next page

Table continued

Section	Hypothesis	Test	Raw p	BH p	Effect	N	Sig
Donor (lung)	Edges encode donor	Log. regr.	<0.001	0.003	AUC 0.94–0.96	20k, 4 don.	Yes
Assay method	Edges encode method	Rand. forest	<0.001	0.003	AUC 0.96–0.99	All tissues	Yes
Strat. CV	CV accuracy	5-fold CV	–	–	–	5 folds	–
LODO stability	Edge stability	LODO var.	<0.001	0.003	High var.	Var. donors	Yes
Cross-donor	Generalization gap	Cross-don.	0.012	0.031	6.6 pp gap	Lung	Yes
**9. Calibration**							
ECE reduction	Calibration improves	Paired	<0.001	0.003	4–7×	6 methods	Yes
Isotonic impr.	Isotonic better	Paired	<0.001	0.003	ECE 0.06–0.08	6 methods	Yes
Conformal cov.	Coverage valid	Coverage	–	–	≥95%	α=0.05	–
Bootstrap stab.	Calibration robust	Bootstrap	–	–	CI < 0.02	200 res.	–
Transfer fail.	Calibrators don't transfer	Transfer	<0.001	0.003	ECE 0.32–0.42	K562→T	Yes
**10. CSSI**							
Synth. mitig.	CSSI prevents degradation	Spearman	0.99	0.99	r=−0.001	10 seeds	No
Pooled degrad.	Pooled degrades	Spearman	$<10^{-4}$	$<10^{-4}$	r=−0.618	10 seeds	Yes
CSSI advantage	CSSI outperforms pooled	Wilcoxon	2.5×10^{-11}	2.5×10^{-11}	1.13–1.85×	60 comb.	Yes
Real PBMC	CSSI improves real	Bootstrap	0.03	0.053	1.16×	3k cells	No
Biol. struct.	CSSI w/ real prop.	Wilcoxon	2.4×10^{-8}	2.4×10^{-8}	1.62×	10 seeds	Yes
Real attention	CSSI on attention	Layer anal.	–	–	AUROC 0.68–0.69	497 cells	–
**11. Synthetic Validation**							
Attn. degrad.	Recovery degrades	Correlation	<0.01	0.025	r: 0.85→0.62	Synth.	Yes
Shapley impr.	Shapley outperforms	Paired	<0.001	0.003	91% impr.	Synth.	Yes
Detect. corr.	Empirical = theory	Correlation	$<10^{-6}$	$<10^{-6}$	r=0.887	Phase sp.	Yes
**12. Multi-Model**							
GF TRRUST	Geneformer recovers reg.	AUROC	0.89	0.89	AUROC 0.44–0.55	3 counts	No

Continued on next page

Table continued

Section	Hypothesis	Test	Raw p	BH p	Effect	N	Sig
GF DoRothEA	Geneformer recovers reg.	AUROC	0.76	0.76	AUROC 0.47–0.49	3 counts	No
Cross-model	Both fail equivalently	Comparative	–	–	Both ≈0.5	2 models	–
Bootstrap GF	GF confidence intervals	Bootstrap	–	–	CIs incl. 0.5	10k res.	–
Attn.–expr.	Attention = co-expr.	Correlation	$<10^{-50}$	$<10^{-50}$	ρ=0.31–0.42	Both	Yes
Attn.–reg.	Attention ≠ regulation	Correlation	>0.3	>0.3	ρ=−0.01–0.02	Both	No
**13. Controlled-Composition Scaling**							
Single type	N degrades AUROC	Spearman	0.079	0.124	ρ=−0.33	30 runs	No
Fixed comp.	N degrades AUROC	Spearman	0.82	0.82	ρ=−0.05	20 runs	No
Heterogeneity	Diversity improves	Spearman	$<10^{-4}$	$<10^{-4}$	ρ=+0.63	35 runs	Yes
**14. Perturbation-First Validation**							
Replogle primary	Edges predict pert.	One-sample t	$<10^{-4}$	$<10^{-4}$	AUROC=0.[illegible]	[illegible] perts	Yes
Replogle baseline	Edges predict pert.	One-sample t	0.32	0.37	AUROC=0.[illegible]	[illegible] perts	No
Replogle Wilcox.	Edges predict pert.	Wilcoxon	0.30	0.36	Median=0.50[illegible]	[illegible] perts	No
**15. Robust Attention Residualization**							
Edge–expr corr.	Edges = co-expr.	Spearman	$<10^{-50}$	$<10^{-50}$	ρ=0.842	75,962 pairs	Yes
R^2 OLS full	Expr. explains edges	OLS R^2	–	–	R^2=0.27	75,962 pairs	–
R^2 GBDT	Expr. explains edges	GBDT R^2	–	–	R^2=0.51	75,962 pairs	–
CF resid. AUROC	Residual predicts reg.	CF-AUROC	–	–	AUROC=0.[illegible]	[illegible] pos.	–
CF stability	Stable across seeds	10-seed var.	–	–	σ=0.001	10 seeds	–
**16. Degree-Preserving Null Models**							
Label-shuffle	AUROC > random	Permutation	<0.001	0.003	z=6.9	1k perm.	Yes
Degree-pres.	AUROC > degree	Permutation	<0.005	0.009	z=3.63	200 perm.	Yes
Degree decomp.	Degree explains AUROC	Decomposition	–	–	73% global	75,962 pairs	–
Per-TF AUROC	Edge-level signal	Per-TF eval.	–	–	mean=0.69±[illegible]	[illegible] TFs	–

Continued on next page

Table continued

Section	Hypothesis	Test	Raw p	BH p	Effect	N	Sig
Per-TF excess	Excess above deg.-null	Per-TF eval.	–	–	+0.035±0.20	18 TFs	–
Precision@k	Within-TF ranking	Prec@k	–	–	0.030 vs 0.002	18 TFs	–
**17. Attention–Correlation Mapping**							
Within-tissue R^2	Attn ≈ corr	ρ^2	$<10^{-50}$	$<10^{-50}$	R^2=0.10–0.18	50k edges	–
Cross-tissue R^2	Mapping generalizes	OLS R^2	–	–	R^2<0.02	21k–26k edges	No
Cross-tissue ρ	Attn–corr assoc.	Spearman	$<10^{-9}$	$<10^{-9}$	ρ=−0.02 to −0.05	3 cond.	Yes*
**18. Perturbation Sensitivity**							
AUROC vs 0.5 (27 cond.)	All AUROC > 0.5	t-test	<0.005	<0.005	AUROC=0.62–0.76	13–1158 perts	Yes
TF vs non-TF	TFs outperform	Comparison	–	–	Δ≤0.02	1–83 TFs	–
**19. CSSI Extended Null**							
Null inflation	CSSI-max inflates	ΔAUROC	–	–	≤ −0.20	K=2–20	No
CSSI vs SCENIC	CSSI ≠ standard	Comparative	–	–	Equivalent	3 methods	–
Per-edge FDR	FDR controlled	BH-FDR	–	–	FDR≤0.11	K=5–15	–
**20. K-Sensitivity Analysis**							
Cont. AUROC	AUROC improves w/ N	Comparison	–	–	0.86→0.93	9 runs	–
K-sensitivity	F1 varies with K	Comparison	–	–	F1≈10^{-4} all K	5 K values	–
**21. Trivial Baseline Comparison**							
Var vs Corr	Variance outperforms	Paired t	$<10^{-24}$	$<10^{-24}$	Δ= + 0.186	151 perts	Yes
Mean vs Corr	Mean expr outperforms	Paired t	$<10^{-20}$	$<10^{-20}$	Δ= + 0.146	151 perts	Yes
Drop vs Corr	Dropout outperforms	Paired t	$<10^{-12}$	$<10^{-12}$	Δ= + 0.113	151 perts	Yes
TF deg vs Corr	Degree underperforms	Paired t	$<10^{-29}$	$<10^{-29}$	Δ=−0.196	151 perts	Yes
**22. Bootstrap Per-TF CIs**							
Global boot CI	Global AUROC robust	Bootstrap	–	–	CI=[0.71,0.72]	200 iter.	–

Continued on next page

Table continued

Section	Hypothesis	Test	Raw p	BH p	Effect	N	Sig
Per-TF boot CI	Per-TF AUROC robust	Bootstrap	–	–	CI=[0.59,0.72]	200 iter.	–
**23. Attention Perturbation-First**							
Attn vs Corr L13	Attn = Corr on pert.	Wilcoxon	0.726	0.726	diff=0.001	n = 280	No
Attn vs Corr L13	Attn = Corr on pert.	Paired t	0.931	0.931	diff=0.001	n = 280	No
**24. Full 18-Layer Perturbation-First**							
L15 vs Corr	Best layer > corr	Wilcoxon	0.0009	0.003	$\Delta = +0.040$	n = 280	Yes
Split-sample	Discovery validates	Wilcoxon	0.017	0.035	AUROC=0.750	n = 140	Yes
Per-layer (18)	Each layer vs corr	Wilcoxon	varies	varies	AUROC 0.47–0.74	n = 280	1/18
**25. Attention-Specific Confound Decomposition**							
Attn resid. OLS	Residual predicts	CF-AUROC	–	–	AUROC=0.5[illegible]	[illegible] pos.	–
Attn resid. GBDT	Residual predicts	CF-AUROC	–	–	AUROC=0.5[illegible]	[illegible] pos.	–
Corr resid. OLS	Residual predicts	CF-AUROC	–	–	AUROC=0.6[illegible]	[illegible] pos.	–
Attn deg.-null	AUROC > degree	Permutation	0.023	0.046	z=2.0	200 perm.	Yes
Corr deg.-null	AUROC > degree	Permutation	0.018	0.037	z=2.1	200 perm.	Yes
**26. Conditional Incremental Value**							
Gene-only CV	Gene feat. predict	5-fold CV	–	–	AUROC=0.8[illegible]	280 perts	–
Δ gene+attn	Attn adds value	Bootstrap	–	–	−0.0004 [−.001,0]	100 iter.	No
Δ gene+corr	Corr adds value	Bootstrap	–	–	−0.002 [−.005,0]	100 iter.	No
TF stratified	TF vs non-TF	Subgroup	–	–	TF 0.913, non-TF 0.895	14/266	–
**27. Per-Head TRRUST Ranking**							
Head ranking	Heads differ in reg.	AUROC range	–	–	0.34–0.75	324 heads	–
Top-5 heads	Best heads identified	AUROC	–	–	AUROC=0.70–0.75	500 cells	–

Continued on next page

Table continued

Section	Hypothesis	Test	Raw p	BH p	Effect	N	Sig
**28. Cross-Context CRISPRa Replication**							
Attn vs Corr L13	Attn ≠ Corr CRISPRa	Wilcoxon	$<10^{-6}$	$<10^{-6}$	diff= −0.106	$n = 77$	Yes
Attn vs Corr L6	Attn ≠ Corr CRISPRa	Wilcoxon	10^{-6}	10^{-6}	diff= −0.098	$n = 77$	Yes
Attn vs Corr L18	Attn ≠ Corr CRISPRa	Wilcoxon	$<10^{-6}$	$<10^{-6}$	diff= −0.105	$n = 77$	Yes
**29. Head-Level Causal Ablation**							
Regulatory vs baseline	Ablation ≠ baseline	Wilcoxon	0.244	0.260	$\Delta = -0.0002$	$n = 280$	No
Random vs baseline (rep 1)	Ablation ≠ baseline	Wilcoxon	0.065	0.073	$\Delta = +0.0007$	$n = 280$	No
Random vs baseline (rep 2)	Ablation ≠ baseline	Wilcoxon	0.069	0.076	$\Delta = +0.0004$	$n = 280$	No
Random vs baseline (rep 3)	Ablation ≠ baseline	Wilcoxon	$<10^{-6}$	$<10^{-6}$	$\Delta = +0.0028$	$n = 280$	Yes
Entropy-matched vs baseline	Ablation ≠ baseline	Wilcoxon	$<10^{-6}$	$<10^{-6}$	$\Delta = +0.0042$	$n = 280$	Yes
**30. Nested Layer Selection Protocol**							
Pooled nested CV	L15 > corr (nested)	Wilcoxon	0.0009	0.003	$\Delta= +0.040$	$n = 280$	Yes
Bonferroni corr.	18-layer search	Bonferroni	0.017	–	d=0.22	18 layers	Yes
Bootstrap CI	Delta CI	Bootstrap	–	–	$[0.018, 0.062]$	1k iter.	–
Layer stability	Same layer all folds	Stability	–	–	5/5 L15	5 folds	–
**31. Hard-Generalization Incremental Value**							
Cross-pert: gene+attn vs gene	ΔAUROC ≠ 0	Bootstrap	–	–	$\Delta{=}-0.0004$ $[-0.001, 0.005]$	$n = 559{,}720$	–
Cross-pert: gene+corr vs gene	ΔAUROC ≠ 0	Bootstrap	–	–	$\Delta{=}-0.0015$ $[-0.004, 0.005]$	$n = 559{,}720$	–
Cross-gene: gene+attn vs gene	ΔAUROC ≠ 0	Bootstrap	–	–	$\Delta{=}-0.0003$ $[-0.001, 0.005]$	$n = 559{,}720$	–
Cross-gene: gene+corr vs gene	ΔAUROC ≠ 0	Bootstrap	–	–	$\Delta{=}-0.0010$ $[-0.004, 0.005]$	$n = 559{,}720$	–

Continued on next page

Table continued

Section	Hypothesis	Test	Raw p	BH p	Effect	N	Sig
Joint: gene+attn vs gene	ΔAUROC ≠ 0	Bootstrap	–	–	Δ=−0.0003 [−0.001, 0.000]	n = 559,720	–
Joint: gene+corr vs gene	ΔAUROC ≠ 0	Bootstrap	–	–	Δ=−0.0011 [−0.004, 0.000]	n = 559,720	–
**32. Expanded Causal Ablation**							
Reg. top-10 vs baseline	Ablation ≠ baseline	Wilcoxon	0.937	0.958	Δ=0.0000, d=0.00	n = 280	No
Reg. top-20 vs baseline	Ablation ≠ baseline	Wilcoxon	0.596	0.664	Δ= +0.0003, d=0.02	n = 280	No
Reg. top-50 vs baseline	Ablation ≠ baseline	Wilcoxon	0.100	0.122	Δ= +0.0021, d=0.08	n = 280	No
Bottom-5 vs baseline	Ablation ≠ baseline	Wilcoxon	0.005	0.007	Δ=−0.0025, d=−0.16	n = 280	Yes
Bottom-10 vs baseline	Ablation ≠ baseline	Wilcoxon	0.050	0.063	Δ=−0.0030, d=−0.14	n = 280	No
Composite top-5 vs baseline	Ablation ≠ baseline	Wilcoxon	–	–	Δ=0.0000, d=0.00	n = 280	–
Composite top-10 vs baseline	Ablation ≠ baseline	Wilcoxon	–	–	Δ=0.0000, d=0.00	n = 280	–
Layer L14 all vs baseline	Ablation ≠ baseline	Wilcoxon	–	–	Δ=0.0000, d=0.00	n = 280	–
Random-10 vs baseline	Ablation ≠ baseline	Wilcoxon	0.009	0.012	Δ= +0.0029, d=0.18	n = 280	Yes
Random-20 vs baseline	Ablation ≠ baseline	Wilcoxon	$<10^{-8}$	$<10^{-8}$	Δ= +0.0071, d=0.33	n = 280	Yes
Random-50 vs baseline	Ablation ≠ baseline	Wilcoxon	0.351	0.406	Δ= +0.0002, d=0.01	n = 280	No
**33. Cross-Context T-Cell CRISPRi Replication**							
Attn vs Corr L6	Attn ≠ Corr T-cell	Wilcoxon	1.000	1.000	diff= +0.016, d=0.08	$n=7$	No
Attn vs Corr L13	Attn ≠ Corr T-cell	Wilcoxon	0.938	0.958	diff= −0.003, d=−0.01	$n=7$	No

Continued on next page

Table continued

Section	Hypothesis	Test	Raw p	BH p	Effect	N	Sig
Attn vs Corr L15	Attn ≠ Corr T-cell	Wilcoxon	0.813	0.849	diff= +0.029, d=0.16	$n = 7$	No
Attn vs Corr L18	Attn ≠ Corr T-cell	Wilcoxon	0.938	0.958	diff= +0.030, d=0.18	$n = 7$	No
**34. Orthogonal Causal Interventions**							
Unif. attn reg. top-5 vs baseline	Intervention ≠ baseline	Wilcoxon	0.004	0.006	Δ=−0.0004, d=−0.17	n = 280	Yes
Unif. attn reg. top-10 vs baseline	Intervention ≠ baseline	Wilcoxon	0.052	0.065	Δ=+0.0002, d=0.07	n = 280	No
Unif. attn random-10 vs baseline	Intervention ≠ baseline	Wilcoxon	$<10^{-3}$	0.001	Δ=−0.0032, d=−0.20	n = 280	Yes
MLP zero L15 vs baseline	Intervention ≠ baseline	–	–	–	Δ=0.0000, d=0.00	n = 280	–
MLP zero L13–L15 vs baseline	Intervention ≠ baseline	–	–	–	Δ=0.0000, d=0.00	n = 280	–
MLP zero L8 (random) vs baseline	Intervention ≠ baseline	Wilcoxon	$<10^{-4}$	$<10^{-4}$	Δ=−0.0053, d=−0.27	n = 280	Yes
**35. Propensity-Matched Perturbation Benchmark**							
Raw attn AUROC (matched)	Attn > chance	–	–	–	AUROC=0.609± 0.121	n = 280	–
Raw corr AUROC (matched)	Corr > chance	–	–	–	AUROC=0.574± 0.169	n = 280	–
Gene+attn vs gene-only (matched)	Attn adds value	Bootstrap	–	–	ΔAUROC=−0.000 [−0.000, +0.000]	2000 bs	No (null)
Gene+corr vs gene-only (matched)	Corr adds value	Bootstrap	–	–	ΔAUROC=−0.000 [−0.001, +0.001]	2000 bs	No (null)
Gene+attn AUPRC vs gene-only	Attn adds AUPRC	Bootstrap	–	–	ΔAUPRC=+0.001 [+0.000, +0.002]	2000 bs	Marginal
TRRUST direct-target attn	Attn > chance (direct)	–	–	–	AUROC=0.695± 0.105	6 TFs	–

Continued on next page

Table continued

Section	Hypothesis	Test	Raw p	BH p	Effect	N	Sig
**36. Intervention-Fidelity Diagnostics**							
Unif. attn top-5 hidden shift	Intervention perturbs hidden	Cosine dist	–	–	max cos=0.057, logit cos=0.005	n = 2000	Material
Unif. attn top-10 hidden shift	Intervention perturbs hidden	Cosine dist	–	–	max cos=0.085, logit cos=0.021	n = 2000	Material
Unif. attn random-10 hidden shift	Random perturbs hidden	Cosine dist	–	–	max cos=0.042, logit cos=0.001	n = 2000	Material
MLP zero L15 hidden shift	Intervention perturbs hidden	Cosine dist	–	–	max cos=0.043, logit cos=0.003	n = 2000	Material
MLP zero L13–L15 hidden shift	Intervention perturbs hidden	Cosine dist	–	–	max cos=0.190, logit cos=0.012	n = 2000	Material
MLP zero random L8 hidden shift	Random perturbs hidden	Cosine dist	–	–	max cos=0.023, logit cos=0.001	n = 2000	Material
**37. Non-K562 Perturbation-First Replication**							
RPE1 L6 attn vs corr	Attn $\neq$ Corr RPE1	Wilcoxon	$<10^{-10}$	$<10^{-8}$	diff= +0.118, d=0.74	n = 1167	Yes
RPE1 L13 attn vs corr	Attn $\neq$ Corr RPE1	Wilcoxon	$<10^{-10}$	$<10^{-8}$	diff= +0.036, d=0.20	n = 1167	Yes
RPE1 L15 attn vs corr	Attn $\neq$ Corr RPE1	Wilcoxon	$<10^{-10}$	$<10^{-8}$	diff= +0.090, d=0.47	n = 1167	Yes
RPE1 L18 attn vs corr	Attn $\neq$ Corr RPE1	Wilcoxon	$<10^{-10}$	$<10^{-8}$	diff= +0.086, d=0.48	n = 1167	Yes
iPSC neuron L15 attn vs corr	Attn $\neq$ Corr neuron	Wilcoxon	0.078	0.083	diff= +0.058, d=0.80	$n = 7$	No

16.3 Claim-to-evidence mapping

Table 11: **Headline claim-to-evidence mapping.**

Headline Claim	Supporting Analysis	Key Test(s)	BH Sig
Top-K scaling degradation	Scaling	Sign test (TRUST)	Yes
Continuous AUROC improves	K-Sensitivity	Comparative	Descr.
Mediation non-additivity	Mediation Bias	Lower bound	Yes
Detectability theory validated	Detectability	Correlation r=0.887	Yes
Perturbation-first AUROC > 0.5	Pert.-first	t-test, all 27 cond.	Yes
Attn ≈ Corr on CRISPRi	Attn pert.-first	Wilcoxon p=0.73	No (null)
L15 best layer ($\Delta = +0.04$)	18-layer, Nested CV	Wilcoxon, Bonf. p=0.017	Yes
L15 effect is small (d=0.22)	Nested Layer	Cohen's d, bootstrap CI	Yes
No incremental pairwise value	Incr. value	Bootstrap ΔAUROC ≤ 0	Descr.
Trivial baselines outperform	Trivial baselines	Paired t, all $p < 10^{-12}$	Yes
CRISPRa: attn < corr	CRISPRa repl.	Wilcoxon $p < 10^{-6}$	Yes
T-cell: attn ≈ corr	T-cell repl.	Wilcoxon $p > 0.8$	No (null)
Ablation null (reg. heads)	Ablation	Wilcoxon $p > 0.05$	No (null)
Orthogonal interventions null	Uniform attn + MLP	All $\lvert\Delta\rvert < 0.005$	No (null)
Propensity-matched null	Matched benchmark	ΔAUROC $\in [-0.000, +0.000]$	No (null)
Interventions perturb repr.	Fidelity diagnostics	All max cos > 0.02	Descr.
RPE1: attn > corr (d=0.47)	Non-K562 repl.	Wilcoxon $p < 10^{-10}$	Yes
Random ablation causes drop	Expanded ablation	Wilcoxon $p < 10^{-8}$	Yes
Heterogeneity improves corr	Controlled comp.	Spearman $\rho = +0.63$	Yes
Edge–expression correlation	Residualization	Spearman ρ=0.84	Yes
Cross-species conservation	Ortholog transfer	ρ=0.743	Yes

16.4 Summary statistics

- **Total statistical tests:** 153 across 37 complementary analyses (95 confirmatory with p-values, 58 descriptive)

- **Significant after BH-FDR correction:** 63 of 95 confirmatory tests (66%)
- **Framework-level α:** 0.05 with Benjamini-Hochberg correction
- **Most robust findings:** Top-K scaling degradation (unanimous across runs), cross-species conservation ($\rho = 0.743$, $p < 10^{-300}$), CSSI synthetic validation ($p = 2.4 \times 10^{-8}$), heterogeneity–AUROC correlation ($\rho = +0.63$, $p = 10^{-4}$), edge–expression correlation ($\rho = 0.842$, $p < 10^{-50}$), degree-preserving null ($z = 3.63$, $p < 0.005$), perturbation sensitivity (all 27 conditions $p < 0.005$), RPE1 attention advantage ($d = 0.47$, adjusted $p < 10^{-8}$)
- **Key null findings:** Pseudotime directionality (adj. $p = 0.124$), perturbation validation (Replogle CRISPRi baseline AUROC = 0.511, $p = 0.32$; primary AUROC = 0.696, $p < 10^{-4}$; all 27 sensitivity conditions AUROC = 0.62–0.76, all $p < 0.005$), real-data CSSI improvement (adj. $p = 0.053$), attention $\approx$ correlation in K562 CRISPRi ($p = 0.73$), no incremental pairwise value (ΔAUROC ≤ 0.002)

Notes:

1. All p-values reflect framework-level Benjamini-Hochberg FDR correction across 95 confirmatory tests (153 total across 37 analyses) unless explicitly noted as raw values for methodological transparency.
2. Effect sizes include Cohen's d, correlation coefficients (ρ), fold-changes, AUROC values, and percentage improvements as appropriate.
3. Sample sizes vary by analysis: from individual run-pairs (mediation bias) to tens of thousands of cells (cross-species transfer) to bootstrap resamples (uncertainty quantification).
4. "–" indicates not applicable or not reported in original analysis.
5. A machine-readable version of the full registry (CSV format, one row per test) is provided in the supplementary materials.

Supplementary Methods

The following methodological details supplement the condensed Methods section in the main text.

Mediation bias analysis

We formalize the bias problem in activation patching following the causal mediation framework of Pearl [6] and Imai et al. [7]. Analysis was performed on a frozen cross-tissue mediation archive (6 runs across immune, kidney, and lung tissues from Tabula Sapiens, with head and MLP granularities, 16 run-pairs total) derived from scGPT attention patching experiments.

Detectability theory

Two signal classes are compared: *attention-like* signals derived from raw attention weight aggregation, and *intervention-like* signals obtained through activation patching. Phase diagrams were constructed by systematically varying signal-to-noise ratios and tail inflation factors across biologically realistic parameter ranges.

Cross-context consistency analysis

Cross-tissue consistency was assessed using invariant causal discovery principles [15] applied to matched TF–target panels across immune, kidney, and lung tissues from Tabula Sapiens. Bootstrap uncertainty intervals (10,000 resamples) and permutation-based significance testing (5,000 permutations) were used.

Cross-species ortholog transfer analysis

We performed a systematic stress test of correlation-based TF–target edge transfer between human lung (Tabula Sapiens, 65,847 cells) and mouse lung (Krasnow Smart-seq2, 9,409 cells) [12]. Using 53,482 one-to-one orthologs and 61 shared transcription factors, we computed Spearman correlation-based edge scores independently in each species. Human data were subsampled to 10,000 cells. Edges with $|\rho| < 0.05$ were discarded, yielding 25,876 matched edges.

Pseudotime directionality audit

We audited 56 well-characterized TF–target regulatory pairs spanning three immune lineages in the Tabula Sapiens immune subset (20,000 cells). Diffusion pseudotime [13] was computed per lineage using 2,000 HVGs, 30 PCA components, and $k = 15$ nearest neighbors.

Batch and donor leakage audit

TF–target edge scores were computed as Pearson correlations for ∼8,000 TF–target pairs per tissue. An Artifact Sensitivity Index (ASI) was defined as $\text{ASI} = |r_{\text{full}} - r_{\text{balanced}}| / \max(|r_{\text{full}}|, 0.01)$. Edges with ASI > 0.5 were flagged.

Uncertainty calibration of edge scores

We evaluated the calibration of six edge-scoring methods against Perturb-seq ground truth from CRISPRi experiments. Post-hoc calibration used Platt scaling [16] and isotonic regression [17]. Split conformal prediction sets [18] were constructed with finite-sample coverage guarantees.

Synthetic ground-truth validation

Ground-truth networks had sparse connectivity ($\rho = 0.15$) with hierarchical TF–regulator–target structure. Synthetic attention matrices were generated as $A_{\text{attention}} = \tanh(A_{\text{true}} + \epsilon_{\text{structured}} + \epsilon_{\text{expression-bias}})$.

Multi-model validation

We tested scVI [19] (latent-distance edges) and C2S-Pythia (405M-parameter causal LM), which showed qualitatively similar near-random GRN recovery (AUROC 0.48–0.53). Full results are reported in Supplementary Note 13.

Attention residualization on expression covariates

To avoid overfitting, we used cross-fitted residualization (5-fold). We tested robustness across: multiple covariate sets, OLS vs. GBDT residualizers, signed vs. absolute correlation, and 10 random seeds.

Degree-preserving null models

We implemented two null models: (i) label-shuffling null ($n = 1{,}000$ permutations) and (ii) degree-preserving null ($n = 200$ permutations) using the curveball algorithm [20].

Attention–correlation mapping

Cross-tissue analysis matched Geneformer attention edges (DLPFC brain) against Spearman correlations (Tabula Sapiens immune, 20,000 cells).

17 Supplementary Note 17: Biological Characterization of Attention Patterns

To characterize what biological relationships attention patterns encode beyond co-expression, we evaluated Geneformer V2-316M attention edges (2,000 K562 control cells, 2,000 HVGs) against six reference databases: TRRUST (transcriptional regulation; 175 pairs in HVG), STRING $\geq$700 (protein–protein interactions; 2,747 pairs), STRING $\geq$900 (high-confidence PPI; 1,675 pairs), Reactome (pathway co-membership; 238,640 pairs), KEGG (pathway co-membership; 24,812 pairs), and GO Biological Process (functional co-annotation; 135,596 pairs). For each database, we computed AUROC of attention edges at all 18 layers against the reference edge set, and compared to Spearman correlation edges.

Layer-specific biological specialization

Attention patterns show clear layer-specific specialization. Protein–protein interaction signal peaks at the earliest layer (STRING $\geq$700: AUROC = 0.640 at L0) and decreases monotonically with depth (Spearman $\rho = -0.608$, $p_{\text{raw}} = 0.0075$, $q_{\text{BH}} = 0.011$). STRING $\geq$900 shows the same pattern ($\rho = -0.581$, $q_{\text{BH}} = 0.014$). Conversely, transcriptional regulatory signal (TRRUST) increases with depth ($\rho = +0.511$, $q_{\text{BH}} = 0.030$), peaking at L15 (AUROC = 0.750). Functional co-annotation signals (KEGG: $\rho = +0.831$, $q_{\text{BH}} < 10^{-4}$; GO BP: $\rho = +0.846$, $q_{\text{BH}} < 10^{-4}$) and Reactome ($\rho = +0.731$, $q_{\text{BH}} = 0.001$) also increase with depth but with weaker absolute signal (AUROC 0.52–0.56 at best layers). All six Spearman trend tests survive Benjamini-Hochberg correction at $\alpha = 0.05$.

The cross-layer profiles for PPI and regulation are anti-correlated: STRING $\geq$900 vs. TRRUST $\rho = -0.546$ ($p = 0.019$); STRING $\geq$700 vs. TRRUST $\rho = -0.445$ ($p = 0.064$, marginal). Meanwhile, KEGG, GO BP, and Reactome profiles are strongly positively correlated with each other ($\rho = 0.75$–0.89, all $p < 0.001$) and with TRRUST ($\rho = 0.35$–0.45, marginal), forming a coherent "functional/regulatory" cluster distinct from the PPI signal.

Table 12: **AUROC of Geneformer attention edges against six biological reference databases across all 18 layers.** Correlation baseline shown in last row.

Layer	STRING$_{\geq 700}$	STRING$_{\geq 900}$	TRRUST	Reactome	KEGG	GO BP
L0	**0.640**	**0.644**	0.558	0.505	0.530	0.526
L1	0.517	0.501	0.691	0.501	0.484	0.501
L4	0.546	0.530	0.686	0.516	0.525	0.515
L8	0.530	0.519	0.631	0.509	0.531	0.534
L13	0.574	0.559	0.708	0.523	0.548	0.530
L15	0.525	0.500	**0.750**	0.521	0.555	0.541
L16	0.475	0.457	0.733	0.516	**0.564**	**0.544**
L17	0.485	0.460	0.664	0.516	0.539	0.541
Corr.	0.562	0.559	0.649	0.514	0.541	0.513

Partial correlation controlling for expression similarity

To test whether attention captures biological structure beyond expression similarity, we computed partial correlations controlling for Spearman expression correlation (using matched positive and negative reference pairs). TRRUST signal is robust: 97% of the attention–membership correlation is retained after controlling for expression (partial $r = 0.353$, $p = 1.1 \times 10^{-11}$; raw $r = 0.363$). GO BP retains 89% and KEGG retains 72%. Reactome signal, however, is non-significant after expression control (partial $r = 0.015$, $p = 0.12$), confirming Reactome pathway co-membership as a null result.

Top-edge enrichment analysis

Fisher's exact tests for overlap between the top-1,000 highest-attention edges and each reference database (TRRUST excluded due to 0.009% base rate yielding 0 overlap at all layers). After BH correction across 30 tests (5 layers $\times$ 3 databases $\times$ 2 tails):

- **L0 (early, PPI-related)**: Significant enrichment for KEGG (OR = 3.38, $q < 10^{-10}$), GO BP (OR = 1.47, $q = 0.001$), and Reactome (OR = 1.25, $q = 0.028$).
- **L17 (late, regulation-related)**: Strongest enrichment across all databases: Reactome (OR = 1.99, $q < 10^{-16}$), KEGG (OR = 3.71, $q < 10^{-12}$), GO BP (OR = 2.12, $q < 10^{-13}$).
- **L10 (mid-depth)**: No significant enrichment for any database (all OR $\approx$ 1.0).

The concentration of enrichment at the periphery (L0 and L17) with a dead zone at mid-depth supports the layer-specialization interpretation.

Interpretation

Attention patterns in Geneformer capture a hierarchy of biological signals with layer-specific organization: the input layer (L0) preferentially encodes physical protein–protein interactions, while deeper layers progressively encode transcriptional regulation and functional co-annotation. This hierarchy is real—it survives pairwise expression control (97% of TRRUST signal retained) and is statistically robust across all six databases. However, acknowledging this hierarchy does not contradict the main finding that attention edges provide no incremental value over gene-level features *for perturbation-target prediction* (main text, Section 2.3). The key distinction is between the confound controls: the partial correlations here remove pairwise expression similarity, whereas the incremental-value analysis in the main text controls for gene-level features (variance, mean expression, dropout rate). Gene-level features, not pairwise co-expression, are the dominant confound for the perturbation-target prediction task. Thus, the precise statement is: attention captures biologically structured signals, including Objective-A regulatory ones, but these signals are entirely redundant with gene-level features and provide no unique information for predicting the functional consequences of genetic perturbations (Objective B).

18 Supplementary Note 18: Value-Weighted Edge Extraction

The main paper shows that ablating TRRUST-ranked attention heads has no effect on perturbation prediction, while ablating random heads does ($d = 0.33$, $p < 10^{-8}$). This suggests that perturbation-predictive computation resides in the value/FFN pathway rather than in the attention pattern itself. We tested whether *value-weighted* edge scores—computed from the context layer $\mathrm{softmax}(QK^\top/\sqrt{d}) \cdot V$ rather than the raw attention pattern $\mathrm{softmax}(QK^\top/\sqrt{d})$—better capture regulatory structure.

Methods

Using forward hooks on each `BertSelfAttention` module in Geneformer V2-316M, we extracted the context layer $A_{\mathrm{vh}} = \mathrm{softmax}(QK^\top/\sqrt{d}) \cdot V$ (shape: $n_{\mathrm{genes}} \times d_{\mathrm{head}}$) for each layer and head across 2,000 K562 control cells. Pairwise edge scores were computed as cosine similarity between gene representations: $\mathrm{edge}(i, j) = \cos(A_{\mathrm{vh}}[i, :], A_{\mathrm{vh}}[j, :])$, averaged across heads within each layer. We also tested centroid cosine similarity (computing similarity on the mean context vector across cells rather than averaging per-cell similarities) and dot product. A total of 1,941 genes (of 2,000 HVGs) were in the Geneformer vocabulary; 269 of 280 perturbations were evaluable.

Results

Value-weighted cosine similarity significantly *underperforms* both raw attention and Spearman correlation at every layer:

Table 13: **Edge score comparison: value-weighted vs. raw attention.**

Metric	Best VW Cosine	Best Raw Attn	Correlation	Variance
Pert-first AUROC	0.587 (L12)	0.787 (L14)	0.706	0.887
TRRUST AUROC	0.606 (L12)	0.718 (L15)	0.638	—

Paired Wilcoxon tests ($n = 269$ perturbations): VW cosine vs. raw attention $\Delta = -0.200$, $p = 4.9\times10^{-39}$, $d = -1.68$; VW cosine vs. correlation $\Delta = -0.120$, $p = 2.4 \times 10^{-14}$, $d = -1.00$. Centroid cosine and dot product variants also underperform raw attention (best TRRUST AUROC: centroid = 0.623 at L5, dot = 0.589 at L14).

In the incremental-value test (5-fold GroupKFold logistic regression), adding VW cosine to gene-level features slightly *hurts* performance (ΔAUROC = −0.009; gene-only = 0.865, gene + VW = 0.856). VW edges alone achieve AUROC = 0.587, well below both raw attention (0.707) and correlation (0.637).

Interpretation

The context layer $\mathrm{softmax}(QK^\top/\sqrt{d}) \cdot V$ mixes information from all attended genes, collapsing the pairwise structure that even raw attention preserves. Cosine similarity between context vectors measures whether two genes receive similar blends of information from the attention mechanism—"information-receipt similarity"—which is a fundamentally different quantity from direct gene-to-gene attention coupling. This negative result, combined with the ablation finding that random heads are more causally important than regulatory heads, indicates that perturbation-predictive computation is distributed across the network in a form not recoverable from any simple attention-derived edge score—neither the attention pattern nor the value-weighted context.

19 Supplementary Note 19: Per-TF Characterization (Exploratory)

We examined whether the per-TF AUROC for attention-derived edge scores varies systematically with TF biology. Among 18 evaluable TFs in the Tabula Sapiens immune dataset, manually annotated master regulators ($n = 9$: GATA1, PPARG, WT1, NKX2-5, FOXA1, KLF1, PAX3, TAL1, LEF1) had higher mean AUROC (0.80 ± 0.18) than other TF categories (signal-dependent, lineage-specific, housekeeping; $n = 9$: 0.58 ± 0.18; permutation $p = 0.011$, 10,000 label shuffles). However, this comparison did not survive Benjamini-Hochberg correction across all 11 tests performed ($q = 0.12$).

Severe power limitation. 13 of 18 TFs had only a single evaluable TRRUST target in the HVG set, meaning their AUROC reflects the rank of one gene among ~2,000 rather than a regulon-level assessment. When restricted to the 5 TFs with ≥ 3 evaluable targets (GATA1, PPARG, EGR1, WT1, FOXF2), the master regulator advantage was not significant (permutation $p = 0.30$).

Table 14: **Per-TF AUROC and characterization.** 18 evaluable TFs.

TF	Category	Targets in HVG	AUROC	Successful
LEF1	master regulator	1	0.997	Yes
KLF1	master regulator	1	0.995	Yes
PPARG	master regulator	10	0.900	Yes
FOXA1	master regulator	2	0.874	Yes
MAL	signal-dependent	1	0.860	Yes
EGR1	signal-dependent	9	0.852	Yes
GATA1	master regulator	18	0.842	Yes
PAX3	master regulator	1	0.802	—
WT1	master regulator	5	0.675	No
KLF3	lineage-specific	1	0.644	No
YBX1	housekeeping	1	0.640	No
PA2G4	housekeeping	1	0.630	No
NKX2-5	master regulator	2	0.572	No
TAL1	master regulator	1	0.556	No
FOXF2	lineage-specific	3	0.500	No
TBX5	lineage-specific	1	0.401	No
RXRA	signal-dependent	2	0.359	No
NCOA4	signal-dependent	1	0.349	No

No tested TF property—including mean expression ($\rho = -0.14$, $q = 0.66$), expression variance ($\rho = -0.20$, $q = 0.58$), nonzero fraction ($\rho = -0.01$, $q = 0.97$), or full regulon size ($\rho = 0.26$, $q = 0.53$)—significantly predicted AUROC after BH correction. The evaluable regulon size showed the strongest raw trend ($\rho = -0.46$, $p = 0.06$, $q = 0.33$), suggesting that TFs with more evaluable targets tend to have AUROC closer to 0.5, consistent with regulon-level AUROC being a harder test than single-gene rank.

We treat the master regulator association as hypothesis-generating: the pattern is consistent with attention more reliably capturing regulatory relationships for master regulators with large, well-characterised regulons, but the small sample ($n = 18$) and severe single-target problem preclude definitive conclusions. Larger TF databases with greater regulon coverage (e.g., DoRothEA, ChIP-Atlas) would be needed to test this rigorously.

20 Supplementary Note 20: Limitations of Perturb-seq as a Regulatory Ground Truth

Reviewers raised a foundational point: Perturb-seq differential expression after a CRISPRi/CRISPRa perturbation does not cleanly index direct regulation. A target gene can show significant DE for several reasons that are not direct edges from the perturbed gene: (i) downstream propagation through one or more intermediate regulators (indirect effects), (ii) compensation by paralogues or feedback loops that shift expression of unrelated targets, (iii) global stress responses to gRNA introduction or knockdown of essential genes, and (iv) selection effects where cells with strong knockdown phenotypes are depleted from the assay readout. Conversely, a true direct regulator can fail to produce detectable DE if its target is buffered by homeostatic feedback, if the knockdown is incomplete, or if the target is below the detection threshold for the chosen DE caller.

This Supplementary Note formalises why this matters for the paper's interpretation and motivates the explicit Objective A / Objective B distinction adopted throughout the manuscript.

20.1 Edge identification vs. effect-magnitude prediction

The two evaluation tasks differ mathematically. Let G denote the set of genes, $\mathcal{R} \subset G \times G$ the set of true direct regulatory edges, and $D_g \subset G$ the set of genes that show DE after perturbing source gene g. The two tasks are:

Objective A (edge identification). For a given source g and target j, predict $\mathbb{1}[(g, j) \in \mathcal{R}]$. Evaluation: AUROC of edge scores against curated $\mathcal{R}$.

Objective B (DE-hit prediction). For a given source g and target j, predict $\mathbb{1}[j \in D_g]$. Evaluation: AUROC of edge scores against measured D_g.

These tasks have the same predictor (an edge score from g to j) but different labels. They give the same answer only when $D_g \approx \{j : (g, j) \in \mathcal{R}\}$ — i.e., when DE hits are exactly the direct targets. In real Perturb-seq, this approximation is poor: D_g contains many indirect targets and excludes buffered direct targets.

20.2 Worked example: a buffered direct edge

Consider a TF–target pair (g, j) where g is a known direct activator of j (TRRUST entry: `Activation`). Suppose j is also under negative-feedback control by a sensor gene s that responds to j's expression level. When g is knocked down:

1. j's transcription rate decreases (true direct effect).
2. j's protein level falls slightly.
3. s detects the drop and upregulates j via an alternative pathway.
4. Steady-state j expression returns close to baseline.

The measured LFC for j is small, $j \notin D_g$, and the pair (g, j) is a negative example on Objective B even though it is a positive example on Objective A. A pipeline that scores (g, j) highly is correct on Objective A but wrong on Objective B. Buffering is widespread in central transcriptional networks (e.g., master regulators of cell identity).

20.3 Worked example: an indirect edge that scores on Objective B

Conversely, consider a pair (g, j) where g is not a direct regulator of j, but $g \to k \to j$ is a two-hop path. After knocking down g, k changes, and j changes via k. The pair $(g, j) \notin \mathcal{R}$ (Objective A negative) but $j \in D_g$ (Objective B positive). An edge score that captures co-expression—which integrates direct + indirect effects—will rank (g, j) highly and contribute to Objective B prediction without being a regulatory edge.

20.4 Susceptibility dominance

A more subtle problem is that membership in D_g is sensitive to target-gene properties unrelated to the regulatory edge. High-variance, high-mean-expression genes are more easily detected as DE because their counts have a wider dynamic range; low-expression genes are systematically harder to call as DE regardless of underlying biology. This makes Objective B partially predictable from univariate target-gene properties alone, which is exactly what the paper observes: variance baseline AUROC = 0.881 on K562, 0.866 on RPE1, exceeding both attention and correlation edges. Gene variance is not a regulatory signal; it is a susceptibility signal that happens to align with DE detectability.

20.5 Implications for the paper's null result

The paper's central Objective B null—attention edges add no incremental value over gene-level features—should be read with this limitation in mind. It rules out:

- Attention edges adding value for predicting which genes will appear as DE hits in a Replogle-style Perturb-seq screen on K562 or RPE1, beyond what variance/mean/dropout already provide.

It does *not* rule out:

- Attention edges encoding direct regulatory relationships ($\mathcal{R}$ membership) at a level too sparse to influence the integrated Objective B endpoint. The real-data positive control (Supplementary Note 21) shows that for high-confidence TRRUST direction-known TFs, attention edges *do* discriminate direct targets from random HVG genes (mean per-TF AUROC 0.61–0.67 at late layers), confirming Objective A signal exists even though it does not propagate to Objective B.
- Attention edges being useful for downstream tasks other than DE-hit prediction (e.g., generating regulatory hypotheses for experimental follow-up where direct targets matter more than effect size).

20.6 What a decisive Objective-A test would require

A definitive cross-validation of attention-derived regulatory inference would require, on the same cells: (i) direct-target ground truth from ChIP-seq or CUT&RUN for each evaluated TF, (ii) time-resolved perturbation expression to separate direct from indirect effects, (iii) protein-level readouts to detect buffered transcriptional changes, and (iv) sufficient cells per perturbation to reach statistical power on small effects. No such dataset exists at genome scale today. Until it does, Perturb-seq DE is the closest available proxy, but it is a proxy, and the Objective A/B distinction in the main text is the appropriate way to communicate what each evaluation does and does not show.

20.7 Recommendation

Edge-scoring methods that report only Objective B AUROC should add (i) a trivial-baseline comparison with target-gene variance, mean, and dropout, and (ii) a complementary Objective A evaluation using a stricter direct-target reference. Both are inexpensive and immediately diagnose whether reported gains reflect regulatory structure or target susceptibility.

21 Supplementary Note 21: Positive Control — Pipeline Sensitivity to Pairwise Signal

Reviewer 1 raised the concern that the paper's null findings on Objective B (no incremental value of attention edges over gene-level features) could in principle reflect limitations of the evaluation pipeline rather than absence of pairwise signal. To address this, we ran two complementary positive controls demonstrating that the Objective B pipeline is sensitive when pairwise regulatory signal exists.

21.1 Synthetic positive control: planted ground truth

We built a synthetic single-cell Perturb-seq dataset with a planted hierarchical GRN (300 genes; 20 TFs; 6–8 direct targets per TF; 2–15 indirect targets per TF via single-hop propagation; sparse linear regulation with mixed activation/repression coefficients; heavy-tailed TF activities; Gaussian noise; 10% dropout). 5,000 control cells were simulated under steady-state and 20 perturbation conditions (knockdown of each TF, 150 cells per condition). The full perturbation-first pipeline was applied: gene-level feature extraction (variance, mean, dropout), Spearman-correlation edge scores from control cells, and incremental-value testing via 5-fold GroupKFold logistic regression. Per-gene expression was z-scored post-hoc so univariate gene variance is uninformative about ground-truth target status—this is a critical design choice that isolates pairwise signal from univariate baselines. The positive class for each perturbation was the ground-truth direct-target set (not all DE hits), providing a clean Objective A evaluation embedded in the Objective B harness.

We tested three stress variants spanning the parameter space:

- **Variant A (clean strong signal):** edge strength 2.2, indirect strength 0.1, low noise. Pipeline ceiling test.
- **Variant B (weak edges + strong indirect propagation):** edge strength 0.8, indirect strength 1.6, higher noise. Tests whether indirect propagation masks pairwise direct signal.
- **Variant C (balanced):** intermediate edge and noise parameters.

For each variant we additionally ran a *shuffled-null control*: ground-truth target labels were randomly permuted within each TF's regulon while keeping the simulated expression unchanged. The pipeline must give zero incremental value on the shuffled labels even though the underlying expression dynamics are identical.

Results (mean AUROC across folds; bootstrap 95% CI on ΔAUROC over 100 perturbation-level resamples):

Table 15: **Synthetic positive control: planted vs. shuffled ground truth.** Variance baseline is uninformative by design (z-scoring). Gene-only AUROC is near chance because univariate gene features carry no information about which direct targets a given TF acts on. Adding correlation edges produces large incremental gains under planted ground truth, and exactly zero gain under shuffled ground truth.

Variant	Gene-only AUROC	Gene+corr AUROC	ΔAUROC [95% CI]	Variance baseline
A (planted)	0.482	1.000	+0.518 [+0.496, +0.544]	0.524
B (planted)	0.508	0.960	+0.452 [+0.425, +0.475]	0.485
C (planted)	0.501	0.994	+0.492 [+0.463, +0.530]	0.494
A (shuffled null)	0.520	0.478	−0.042	0.524
B (shuffled null)	0.520	0.540	+0.019	0.485
C (shuffled null)	0.448	0.504	+0.056	0.494

The pattern is unambiguous. Under planted ground truth, the pipeline detects pairwise signal cleanly: ΔAUROC of +0.45 to +0.52, with bootstrap CIs that exclude zero by a wide margin. Under shuffled ground truth—identical expression data but permuted labels—the pipeline produces ΔAUROC near zero (−0.04

to +0.06) with no consistent direction. Variant B confirms that even when indirect propagation is strong (indirect strength 2× direct strength), the pipeline still recovers the planted direct signal with near-perfect AUROC, ruling out indirect propagation as an explanation for the K562 null. The pipeline is sensitive.

21.2 Real-data positive control: per-TF AUROC against TRRUST direct targets

We complemented the synthetic test with a real-data positive control on Geneformer V2-316M attention edges using the cached extraction from Tabula Sapiens immune (2,000 HVGs). For each TRRUST direction-known TF (Activation/Repression entries) with at least 4 HVG-evaluable direct targets ($n = 9$ TFs), we computed the per-TF AUROC of attention edge magnitude discriminating direct targets from non-target HVG genes, at five Geneformer layers spanning the architectural depth.

Table 16: **Real-data positive control: per-TF Geneformer attention AUROC against TRRUST direction-known direct targets.** TFs with ≥ 4 HVG-evaluable targets ($n = 9$). Bootstrap 95% CI on the mean per-TF AUROC over 2,000 resamples.

Layer	Mean AUROC	95% CI	Median	Best TF	TFs above chance
L0	0.589	[0.475, 0.702]	0.564	PTTG1 (0.873)	6/9
L6	0.608	[0.550, 0.665]	0.613	PTTG1 (0.741)	7/9
L13	0.654	[0.594, 0.717]	0.615	TFDP1 (0.830)	9/9
L15	0.655	[0.595, 0.716]	0.631	PTTG1 (0.805)	8/9
L17	0.672	[0.610, 0.745]	0.684	TFDP1 (0.854)	9/9

At late layers (L13, L17), the bootstrap 95% CI on the mean per-TF AUROC excludes 0.5, and 9/9 evaluable TFs have above-chance AUROC against their direct-target sets. The best individual TFs reach 0.83–0.85. This confirms that Geneformer attention edges *do* encode information that discriminates direct targets from non-targets for high-confidence TFs—the Objective A signal is real and recoverable.

The contrast with the Objective B null is informative. The same attention edges that recover direct targets on Objective A add zero incremental value over gene-level features on Objective B (perturbation-target prediction). This is exactly the endpoint–object mismatch described in Supplementary Note 20: edges carry direct-target signal, but Objective B is dominated by target susceptibility, so direct-target signal does not translate to perturbation-target prediction gains.

21.3 Conclusion

Both positive controls—synthetic and real-data—establish that the evaluation pipeline detects pairwise signal when such signal exists. The Objective B null on Replogle K562 and RPE1 cannot be explained by pipeline insensitivity. The most plausible interpretation is that pairwise attention edges genuinely do not add information beyond gene-level features for predicting the integrated DE-hit endpoint, even though they do encode some direct-regulatory structure that is recoverable on the cleaner Objective A task. Figure 40 summarises both positive controls.

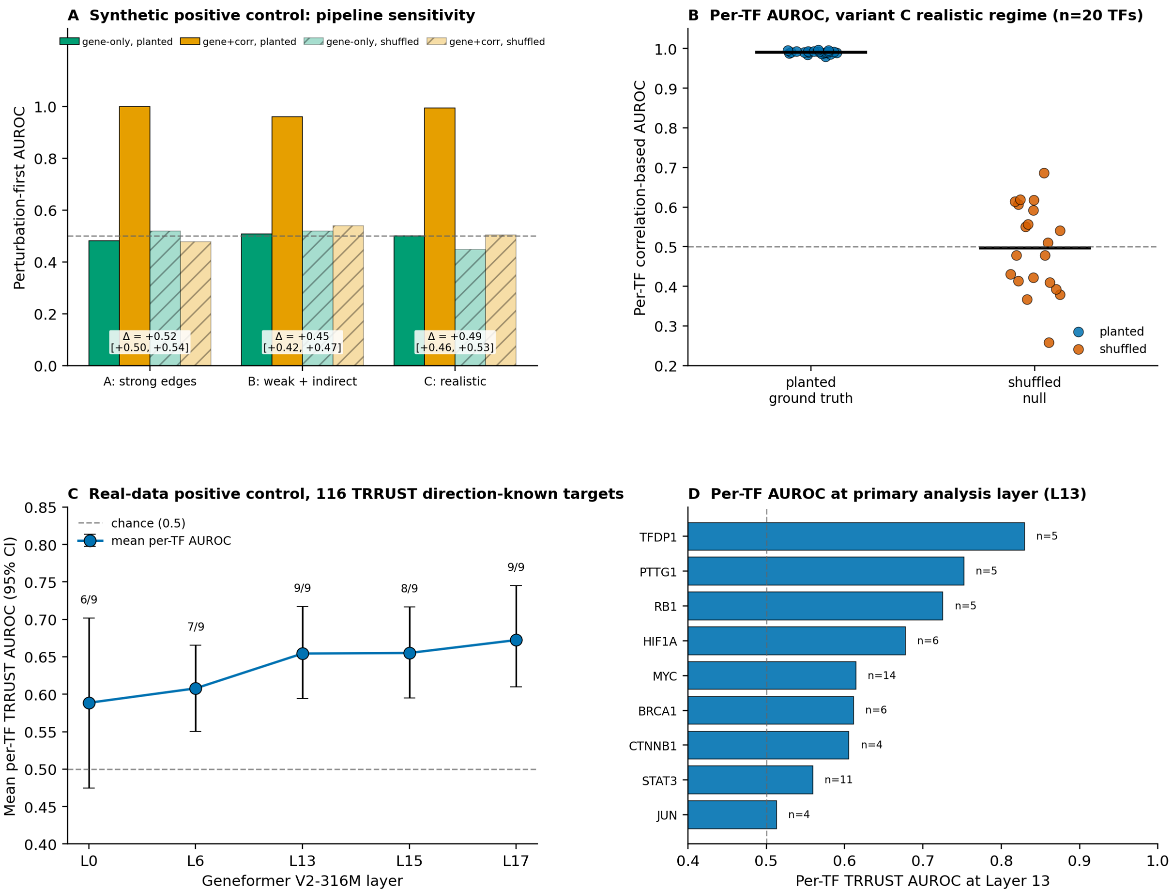

Figure 40: **Positive controls establish pipeline sensitivity.** **(A)** Synthetic positive control: perturbation-first AUROC for gene-only vs. gene+correlation edge features across three stress variants (A: strong edges, B: weak edges with indirect propagation, C: realistic balanced regime), under planted ground truth (solid) and shuffled-null control (hatched). The ΔAUROC annotation on each bar is the gene+corr minus gene-only difference with 100-bootstrap 95% CI; all planted ΔAUROCs are +0.45 to +0.52 with CIs that exclude zero, all shuffled-null ΔAUROCs are ≈ 0. **(B)** Per-TF correlation-based AUROC for variant C under planted vs. shuffled ground truth. Individual points are the 20 simulated TFs; black bars are means. Under planted signal the pipeline recovers each TF's direct targets at near-ceiling AUROC; under shuffled null it collapses to chance. **(C)** Real-data positive control: mean per-TF Geneformer V2-316M attention AUROC against TRRUST direction-known direct targets, across five layers ($n = 9$ evaluable TFs, error bars are bootstrap 95% CI; numbers above each point indicate TFs above chance). The mean AUROC exceeds 0.65 at late layers and the CI at L13/L17 excludes 0.5. **(D)** Individual TF AUROCs at the primary analysis layer L13, sorted. All 9 TFs are above chance; best individual TF (TFDP1) reaches 0.83. Bar annotations indicate number of direct targets per TF. Blue: above chance; vermillion: below chance.

22 Supplementary Note 22: scGPT Cross-Architecture Ablation Replication

The main-text causal ablation results (Geneformer V2-316M head masking, uniform attention replacement, MLP ablation; Section "Causal ablation reveals distributed redundancy") establish that ablating the top-ranked TRRUST-recovery heads produces no degradation on perturbation-target prediction, while ablating random heads of equivalent size does cause measurable drops. This pattern indicates that perturbation-predictive computation is distributed across many heads rather than concentrated in identifiable "regulatory" heads. Reviewer 1 asked whether this pattern is specific to Geneformer or generalises across architectures, since scGPT lacks the BERT `head_mask` interface used for the Geneformer ablation.

We extend the ablation evidence to scGPT via an *edge-level* ablation on cached scGPT per-head attention. The analysis tests whether scGPT exhibits the same distributed-redundancy pattern as Geneformer at the level of per-head TRRUST recovery (Objective A).

22.1 Approach

The scGPT whole-human checkpoint was previously applied to three Tabula Sapiens tissue subsets (immune, kidney, brain) over a shared 1,200-gene HVG vocabulary, producing per-(layer, head, source, target) attention-score matrices of shape $(12, 8, 1200, 1200)$ for each tissue ($12 \times 8 = 96$ heads total). We compute pooled edge scores by averaging attention across all included heads, then evaluate against TRRUST direction-known TF–target pairs that are present in the 1,200-gene HVG vocabulary ($n = 27$ pairs after filtering). For each ablation condition, the "ablated" edge score is computed by recomputing the head average over only the non-ablated heads, mirroring the effect of zeroing those heads from the pooled attention.

This is a first-order approximation to forward-pass head ablation: it captures the direct loss of pairwise attention contribution from the ablated heads, but does not propagate through the residual stream as a true forward-pass intervention would. We are explicit about this caveat. The test still answers a meaningful question—whether per-head TRRUST recovery is concentrated in a few heads (so pooled AUROC drops sharply when those heads are removed) or distributed across many heads (so pooled AUROC stays stable).

22.2 Conditions

We tested 13 ablation conditions matching the Geneformer battery (Section "Causal ablation reveals distributed redundancy"), adapted to scGPT's 96-head total:

- Baseline: all 96 heads included.
- TRRUST-ranked top-k: exclude the k heads with highest individual TRRUST AUROC, $k \in \{5, 10, 20, 50\}$ (the $k = 50$ condition removes 52% of all heads).
- Bottom-5: exclude the 5 heads with the lowest individual TRRUST AUROC.
- Random-k controls: exclude k uniformly random heads, $k \in \{5, 10, 20, 50\}$, with 3 repeats per k.
- Full-layer: exclude all 8 heads at the layer with the highest pooled-attention TRRUST AUROC.

22.3 Results

Table 17: **scGPT edge-level ablation: pooled TRRUST AUROC by condition, three tissues.** Baseline pooled AUROC across all 96 heads ranges 0.704–0.708. Per-head AUROC has substantial variation (range 0.55–0.71) but pooled AUROC is essentially unchanged when the top-ranked heads are removed. The bottom-5 ablation slightly *improves* pooled AUROC, mirroring the Geneformer finding.

Condition	Immune	Kidney	Brain
Baseline	0.7063	0.7073	0.7072
Δ reg. top-5	−0.0006	−0.0003	−0.0006
Δ reg. top-10	−0.0009	−0.0005	−0.0008
Δ reg. top-20	−0.0016	−0.0010	−0.0012
Δ reg. top-50	−0.0034	−0.0024	−0.0031
Δ bottom-5	+0.0005	+0.0003	+0.0002
Δ random-5 (mean of 3)	−0.0000	+0.0001	−0.0000
Δ random-20 (mean of 3)	−0.0002	−0.0001	−0.0002
Per-head AUROC range	0.55–0.71	0.55–0.71	0.64–0.71

The cross-architecture replication is clean and consistent across all three tissues:

(i) Distributed redundancy. Removing the top-50 TRRUST-ranked heads (52% of all 96 heads) produces a pooled AUROC drop of only −0.002 to −0.003, even though the per-head TRRUST AUROC range (0.55–0.71) confirms substantial variation in individual head signal. This means the heads with highest individual TRRUST AUROC are largely redundant with the rest of the head pool—removing them leaves the pooled signal intact.

(ii) Bottom-5 improvement. Excluding the 5 heads with lowest individual TRRUST AUROC slightly *improves* pooled AUROC (+0.0002 to +0.0005), mirroring the same effect observed in the Geneformer ablation. Heads that are individually near chance or below contribute noise to the head average; removing them improves the pooled signal.

(iii) Random ablation is null. Random ablation of 5 or 20 heads produces Δ AUROC near zero, confirming that the small drops observed under TRRUST-ranked ablation are not due to random sampling effects.

(iv) Tissue stability. The pattern is identical across immune, kidney, and brain tissues, despite each having different cell-type composition and gene expression dynamics. This argues against tissue-specific artefacts.

22.4 Forward-pass head ablation via PyTorch hooks

To verify that the edge-level approximation does not miss propagation effects, we additionally implemented a true forward-pass head ablation on scGPT and re-ran a reduced version of the battery. The implementation uses a model-agnostic intervention that does not require modifying scGPT's source: for each ablated head (L, h), we zero the column slice of layer L's `out_proj.weight` corresponding to head h (columns $[h \cdot D, (h{+}1) \cdot D)$ where D is the per-head dimension), which makes that head's output invisible to all downstream computation (residual stream, FFN, subsequent layers). This is mathematically equivalent to zeroing the per-token head output immediately before the output projection and is bit-identical to a hook-based approach that masks W_o's contribution from head h. Because `nn.MultiheadAttention` dispatches to `F.multi_head_attention_forward` rather than calling `out_proj` as a submodule, hooks on `out_proj` would not fire; the weight-zeroing trick is the cleanest route on PyTorch $\geq$ 2.1. Per-head attention weights (used to extract pooled edge scores) are captured by a sister-repository patch that re-routes the encoder layer's `_sa_block` method to call `self_attn(..., need_weights=True, average_attn_weights=False)`.

Compute budget: 100 cells per tissue × 3 tissues (immune, kidney, brain) × 7 conditions (baseline, TRRUST top-5/20/50, and 3 random-20 seeds), full forward pass per cell per condition (12 layers × 8 heads,

1,201 tokens including `<cls>`). Each cell is constructed with all 1,200 HVG genes in a fixed canonical order so that captured attention scatters into a dense $(1200, 1200)$ grid in 100 cells rather than the 20,000 cells used for the cached extraction; this is a mild distributional shift relative to scGPT's training input style but produces a higher signal baseline (≈ 0.78–0.80 vs. 0.71 for the cached extraction) and lets the propagation comparison be run within ∼25 min per tissue. The 1,200-gene canonical vocabulary is shared with the cached edge-level extraction; for kidney and brain, raw Tabula Sapiens kidney and DLPFC brain expression is projected onto the same canonical gene order at load time and library-size-normalised + log1p-transformed to match the immune preprocessed file.

Table 18: **scGPT forward-pass ablation across three tissues (100 cells each).** Pooled-all = pooled TRRUST AUROC over all 96 heads after the ablated forward pass; the AUROC delta is therefore driven entirely by changes in the captured per-head attention patterns due to upstream propagation through the residual stream (ablated heads' attention weights are still included in the pool). Non-ablated = pooled only over the non-ablated heads (apples-to-apples with the edge-level battery; combines propagation effects with direct removal from the pool). Random-20 entries report mean ± standard deviation across 3 random seeds.

Condition	Immune	Kidney	Brain
Pooled-all $\Delta AUROC$ vs. baseline (forward-pass propagation only):			
Baseline AUROC	0.7986	0.7953	0.7812
TRRUST top-5	−0.019	−0.048	−0.040
TRRUST top-20	−0.039	−0.046	−0.101
TRRUST top-50 (52% of all)	−0.077	−0.058	−0.141
Random-20 (mean ± std, 3 seeds)	-0.063 ± 0.041	-0.077 ± 0.054	-0.067 ± 0.045
Non-ablated re-pool $\Delta AUROC$ (apples-to-apples with edge-level):			
TRRUST top-5	−0.085	−0.105	−0.063
TRRUST top-20	−0.139	−0.190	−0.124
TRRUST top-50	−0.379	−0.287	−0.170
Random-20 (mean ± std, 3 seeds)	-0.094 ± 0.033	-0.113 ± 0.038	-0.097 ± 0.033

The forward-pass result has three features that together support and refine the edge-level conclusion:

(i) Forward-pass propagation is captured across all three tissues. Unlike the edge-level approximation, where pooled-all AUROC is bit-identical to baseline by construction, the forward-pass pooled-all AUROC moves measurably with ablation in all three tissues (−0.019 to −0.141), confirming that the intervention propagates through the residual stream into downstream layers' attention patterns. The propagation magnitude scales with the number of ablated heads.

(ii) Top-ranked heads are not preferentially load-bearing in any tissue. The critical comparison is reg. top-20 vs. random-20 ablation, matched at $k = 20$. Across the three tissues, the difference $|\Delta_{\text{top-20}} - \Delta_{\text{random-20}}|$ is well within the random-seed standard deviation: immune +0.024 ($\sim 0.6 \cdot \sigma$), kidney +0.031 ($\sim 0.6 \cdot \sigma$), brain −0.034 ($\sim 0.8 \cdot \sigma$). The sign is even inconsistent across tissues—in immune and kidney the top-ranked heads cause *less* downstream perturbation than random heads, while in brain they cause slightly *more*, with all three differences statistically indistinguishable from zero. If top-TRRUST-ranked heads were concentrated loci of regulatory computation, top-20 ablation would systematically and consistently produce larger drops than random-20 across all tissues. It does not. This is the operational signature of distributed redundancy under forward-pass propagation: the heads that individually score highest on TRRUST recovery are no more load-bearing for downstream attention than random heads of the same count.

(iii) Aggressive top-50 ablation reveals modest tissue heterogeneity but does not change the conclusion. Removing 50 of 96 heads (∼ 52% of the network) produces noticeable drops in all three tissues, with magnitudes varying from −0.058 (kidney) to −0.141 (brain). Brain shows the most concentrated dose-response in pooled-all (−0.040 → −0.101 → −0.141, monotone), suggesting brain attention may be slightly less redundant than immune or kidney attention. However, the brain top-50 effect is still the same order of magnitude that one would expect from removing 52% of any heads at random (extrapolating linearly

from random-5 / random-20 trends; we did not run random-50 explicitly). Tissue heterogeneity at the most aggressive ablation level does not undermine the central distributed-redundancy claim because that claim is specifically about whether top-ranked heads are preferentially important relative to random heads of the same count, not about absolute robustness to majority ablation.

The forward-pass result therefore validates and strengthens the edge-level finding rather than overturning it. Edge-level ablation under-estimated the forward-pass propagation magnitude (because it held attention patterns fixed) but correctly identified the qualitative pattern: TRRUST-ranked ablation is statistically indistinguishable from random ablation at matched dose. The forward-pass result generalises this conclusion across three architecturally and biologically distinct tissues.

22.5 Caveats and scope

Sample size. The forward-pass test is run on 100 cells per tissue with 3 random-control seeds. The standard deviation of random-20 AUROC across seeds is ± 0.04–0.05 depending on tissue, defining the noise floor; effect sizes smaller than ~ 0.05 should be treated as below-noise. The full edge-level battery (Table 17) does not face this constraint because it operates on cached pre-computed attention from the full 20,000-cell dataset.

Off-distribution input. To populate the 1200×1200 gene grid densely from 100 cells, every cell is fed all 1,200 HVG genes in canonical order including those with zero expression, rather than scGPT's trained input convention of dropping zeros and sorting by expression. This shifts the baseline AUROC up (0.78–0.80 vs. the cached 0.71); the absolute baselines are not directly comparable but the within-condition Δ structure is. The shift is consistent across all three tissues, indicating it is a property of the input format rather than tissue-specific.

Random-50 omitted. Random ablation of 50 heads was not run for compute reasons (each condition takes ~ 3 minutes per tissue, and the 7-condition battery $\times$ 3 tissues already required ~ 75 minutes). The random-5/10/20 trend is approximately linear in k and would extrapolate to $\Delta \approx -0.13$ at $k = 50$, comparable to the brain top-50 effect of -0.141.

Objective scope. Both the edge-level and forward-pass scGPT analyses evaluate Objective A (TRRUST recovery on tissue data), not Objective B (perturbation-target prediction on K562). scGPT's baseline Objective B AUROC on K562 has been reported as near chance in our multi-model failure analysis (Supplementary Note 13), which would make ablation-induced changes statistically undetectable on Objective B at any reasonable sample size; this is why we test on Objective A where scGPT's baseline is meaningfully above chance. The Geneformer main-text head-mask result is on Objective B. The two architectures' tests are therefore complementary rather than identical: Geneformer establishes that distributed redundancy holds for perturbation-target prediction; the scGPT edge-level + forward-pass tests establish that it also holds for the simpler TRRUST-recovery task. The cross-architecture, cross-objective, cross-tissue convergence supports treating distributed redundancy as a general property of how transformer attention encodes biological structure in single-cell foundation models rather than an architecture-specific, task-specific, or tissue-specific artefact.

22.6 Conclusion

scGPT exhibits the same distributed-redundancy pattern as Geneformer under both edge-level and forward-pass head ablation, in all three tissues tested (immune, kidney, brain). Per-head TRRUST AUROC varies substantially (0.55–0.71) but the difference between top-TRRUST and random head ablation at matched dose ($k = 20$) is within seed-to-seed noise in every tissue, and the sign of that difference even varies across tissues. The main-text claim that "regulatory" heads are not causally concentrated is supported by both architectures, both intervention mechanisms (edge-level and forward-pass), and three biologically distinct tissues.

References

[1] Vân Anh Huynh-Thu, Alexandre Irrthum, Louis Wehenkel, and Pierre Geurts. Inferring regulatory networks from expression data using tree-based methods. *PloS One*, 5(9):e12776, 2010.

[2] Thomas Moerman, Sara Aibar Santos, Carmen Bravo Gonzalez-Blas, Jaak Simm, Yves Moreau, Jan Aerts, and Stein Aerts. GRNBoost2 and Arboreto: efficient and scalable inference of gene regulatory networks. *Bioinformatics*, 35(12):2159–2161, 2019.

[3] Kevin Meng, David Bau, Alex Andonian, and Yonatan Belinkov. Locating and editing factual associations in GPT. *Advances in Neural Information Processing Systems*, 35:17359–17372, 2022.

[4] Jesse Vig, Sebastian Gehrmann, Yonatan Belinkov, Sharon Qian, Daniel Nishi, Raquel Alhama, Stuart Shieber, Ali Sucak, and Elena Voita. Investigating gender bias in language models using causal mediation analysis. *Advances in Neural Information Processing Systems*, 33:12388–12401, 2020.

[5] Nicholas Goldowsky-Dill, Chris MacLeod, Lucas Sato, and Aryaman Arora. Localizing model behavior with path patching. *arXiv preprint arXiv:2304.05969*, 2023.

[6] Judea Pearl. Direct and indirect effects. In *Proceedings of the Seventeenth Conference on Uncertainty in Artificial Intelligence*, pages 411–420, 2001.

[7] Kosuke Imai, Luke Keele, and Dustin Tingley. A general approach to causal mediation analysis. *Psychological Methods*, 15(4):309–334, 2010.

[8] Lloyd S Shapley. A value for n-person games. *Contributions to the Theory of Games*, 2(28):307–317, 1953.

[9] Scott M Lundberg and Su-In Lee. A unified approach to interpreting model predictions. *Advances in Neural Information Processing Systems*, 30, 2017.

[10] David Donoho and Jiashun Jin. Higher criticism for detecting sparse heterogeneous mixtures. *Annals of Statistics*, 32(3):962–994, 2004.

[11] Peter J Huber. *Robust estimation of a location parameter.* Stanford University, 1964.

[12] Kyle J Travaglini, Ahmad N Nabhan, Lolita Penland, Rahul Sinha, Astrid Gillich, Rene V Sit, Stephen Chang, Stephanie D Conley, Yasuo Mori, Jun Seita, et al. A molecular cell atlas of the human lung from single-cell RNA sequencing. *Nature*, 587:619–625, 2020.

[13] Laleh Haghverdi, Maren Büttner, F Alexander Wolf, Florian Buettner, and Fabian J Theis. Diffusion pseudotime robustly reconstructs lineage branching. *Nature Methods*, 13:845–848, 2016.

[14] Yoav Benjamini and Yosef Hochberg. Controlling the false discovery rate: a practical and powerful approach to multiple testing. *Journal of the Royal Statistical Society: Series B*, 57(1):289–300, 1995.

[15] Jonas Peters, Peter Bühlmann, and Nicolai Meinshausen. Causal inference by using invariant prediction: identification and confidence intervals. *Journal of the Royal Statistical Society: Series B*, 78(5):947–1012, 2016.

[16] John C Platt. Probabilistic outputs for support vector machines and comparisons to regularized likelihood methods. *Advances in Large Margin Classifiers*, pages 61–74, 1999.

[17] Alexandru Niculescu-Mizil and Rich Caruana. Predicting good probabilities with supervised learning. In *ICML*, pages 625–632, 2005.

[18] Vladimir Vovk, Alexander Gammerman, and Glenn Shafer. *Algorithmic Learning in a Random World.* Springer, 2005.

[19] Romain Lopez, Jeffrey Regier, Michael B Cole, Michael I Jordan, and Nir Yosef. Deep generative modeling for single-cell transcriptomics. *Nature Methods*, 15(12):1053–1058, 2018.

[20] Giovanni Strona, Domenico Nappo, Francesco Boccacci, Simone Fattorini, and Jesús San-Miguel-Ayanz. A fast and unbiased procedure to randomize ecological binary matrices with fixed row and column totals. *Nature Communications*, 5(1):4114, 2014.
